# A taxonomic revision of the land snail genus *Acinolaemus* F. G. Thompson & Upatham, 1997 (Gastropoda, Eupulmonata, Hypselostomatidae), with notes on *Clostophis* W. H. Benson, 1860 species from Cambodia, Thailand, and Vietnam

**DOI:** 10.3897/zookeys.1279.186771

**Published:** 2026-05-05

**Authors:** Vukašin Gojšina, András Hunyadi, Chirasak Sutcharit, Piyoros Tongkerd, Kurt Auffenberg, Jozef Grego, Barna Páll-Gergely

**Affiliations:** 1 Department of Morphology, Systematics and Phylogeny of Animals, Faculty of Biology, University of Belgrade, Studentski trg 16, 11000, Belgrade, Serbia Faculty of Science, Chulalongkorn University Bangkok Thailand; 2 Adria sétány 10G 2/5., H-1148 Budapest, Hungary Florida Museum of Natural History, University of Florida Gainesville United States of America; 3 Animal Systematics Research Unit, Department of Biology, Faculty of Science, Chulalongkorn University, Bangkok 10330, Thailand Faculty of Biology, University of Belgrade Belgrade Serbia; 4 Florida Museum of Natural History, University of Florida, 1659 Museum Road, Gainesville, FL, 32611, USA Albert Kázmér Faculty of Mosonmagyaróvár, Széchenyi István University Mosonmagyaróvár Hungary; 5 Horná Mičiná 219, SK-97401 Banská Bystrica, Slovakia Unaffiliated Budapest Hungary; 6 Department of Soil, Water and Natural Sciences, Albert Kázmér Faculty of Mosonmagyaróvár, Széchenyi István University, Vár 2., 9200 Mosonmagyaróvár, Hungary Unaffiliated Banská Bystrica Slovakia

**Keywords:** New species, Pupilloidea, Southeast Asia, taxonomy

## Abstract

In this work, the hypselostomatid land snail genus *Acinolaemus* F.G. Thompson & Upatham, 1997 in Southeast Asia is revised. Characters based on which the genus was originally erected are thoroughly examined and evaluated. The genus is subdivided into six species groups based on similarities in shell surface sculpture and apertural barrier morphology. Four out of five species originally described in *Acinolaemus*, as well as species described from Vietnam and Cambodia are moved to *Clostophis*. Thirteen new species of *Acinolaemus* are described as follows: *A. altus* Gojšina, Hunyadi & Páll-Gergely, **sp. nov**., *A. asper* Gojšina, Tongkerd & Páll-Gergely, **sp. nov**., *A. atypicus* Gojšina, Tongkerd & Páll-Gergely, **sp. nov**., *A. ferox* Gojšina, Tongkerd & Páll-Gergely, **sp. nov**., *A. humilis* Gojšina, Hunyadi & Páll-Gergely, **sp. nov**., *A. microcubus* Gojšina, Hunyadi & Páll-Gergely, **sp. nov**., *A. paucidentatus* Gojšina, Tongkerd & Páll-Gergely, **sp. nov**., *A. profundus* Gojšina, Auffenberg & Páll-Gergely, **sp. nov**., *A. rugolabialis* Gojšina, Hunyadi & Páll-Gergely, **sp. nov**., *A. simplex* Gojšina, Tongkerd & Páll-Gergely, **sp. nov**., *A. singularis* Gojšina, Tongkerd & Páll-Gergely, **sp. nov**., *A. solitus* Gojšina, Hunyadi & Páll-Gergely, **sp. nov**., *A. zed* Gojšina, Hunyadi & Páll-Gergely, **sp. nov**. Thus, the total number of *Acinolaemus* species increases to 19, all inhabiting Thailand and Myanmar.

## Introduction

*Acinolaemus* F. G. Thompson & Upatham, 1997 is a genus of terrestrial, exclusively limestone-dwelling hypselostomatid microgastropods (shell diameter less than 5 mm) with a total of 13 species in Thailand (Thompson and Upatham 1997; Changlom et al. 2019; Tongkerd et al. 2025), Myanmar (Tongkerd et al. 2024), Vietnam, and Cambodia (Vermeulen et al. 2007, 2019). The genus is diagnosed by Thompson and Upatham (1997) based on four main characters: i) an enlarged angular lamella, which is the strongest barrier in the aperture, ii) a distinct sinulus formed by the close arrangement of the angular lamella and the upper palatal plica/palatal tubercle, iii) the surface sculpture of the teleoconch in the form of raised spiral striae crossed by equally strong radial growth lines (radial lines not so pronounced in other genera), iv) and the presence of raised spiral striae on the protoconch. Additionally, several other characters are mentioned by Thompson and Upatham (1997) for *Acinolaemus* but they are not specifically emphasized as they are not typical for all species or are shared with other genera such as: presence of the palatal tubercle (shared with *Bensonella*), occasional presence of hooked barriers (shared with *Clostophis*, *Bensonella* and *Hypselostoma*), presence of a constriction behind the aperture (not in all *Acinolaemus* species), small yellowish (or colorless), straight sided shell and last whorl ascending or descending near the aperture (shared with other genera). When the genus was erected, five newly described species from Thailand were assigned to it: *A. colpodon* F. G. Thompson & Upatham, 1997, *A. ptychochilus* F. G. Thompson & Upatham, 1997 (type species), *A. rhamphodon* F. G. Thompson & Upatham, 1997, *A. sphinctinion* F. G. Thompson & Upatham, 1997 and *A. stenopus* F. G. Thompson & Upatham, 1997. In the same work, *Hypselostoma dayanum* Stoliczka, 1871, *H. laidlawi* (Collinge, 1902) and *Paraboysidia neglecta* van Benthem Jutting, 1961 were also assigned to the genus (Thompson and Upatham 1997). The genus was then reported from Vietnam with two species, *A. carcharodon* Vermeulen, Phung & Truong, 2007 and *A. pyramidalis* (Vermeulen, Phung & Truong, 2007)) (Vermeulen et al. 2007, 2019). Páll-Gergely et al. (2020) assigned *P. neglecta* to the genus *Clostophis*, and the same was done two years later for *H. laidlawi* by Páll-Gergely et al. (2022). The genus *Acinolaemus* was then reported from Cambodia (three species: *A. carcharodon*, *A. pyramidalis*, *A. rectus* Vermeulen, Luu, Theary & Anker, 2019) (Vermeulen et al. 2019) and two more species were described from Thailand (*A. cryptidentatus* Changlom, Chan-ard & Dumrongrojwattana, 2019 and *A. mueangonensis* Changlom, Chan-ard & Dumrongrojwattana, 2019) (Changlom et al. 2019). Tongkerd et al. (2024) followed the concept of Thompson and Upatham (1997) and also treated *Hypselostoma dayanum* Stoliczka, 1871 as a member of *Acinolaemus* and included first records of *A. cryptidentatus* from Myanmar. Lastly, Tongkerd et al. (2025) described two new species from Tak Province in Thailand (*A. corusticorus* Tongkerd & Panha, 2025 and *A. rhamphodontis* Tongkerd & Panha, 2025).

As morphologically similar, *Clostophis* is diagnosed by Páll-Gergely et al. (2020) based on the following traits: small, colorless shell; body whorl rounded or very slightly keeled/shouldered, adnate or detached from the penultimate; strong spiral striation crossed by weaker radial growth lines; aperture with a small (0–5) number of barriers (≤ 2 mentioned in Páll-Gergely et al. (2020) but ≤ 5 mentioned in Páll-Gergely et al. (2022)). The only phylogenetic work that includes members of *Acinolaemus* is that of Tongkerd et al. (2025). However, this work does not include the type species of both *Acinolaemus* and *Clostophis*, so the relationship of the two genera remains unresolved.

Hypselostomatidae are very diverse in terms of shell shape, sculpture, and apertural barrier arrangement. This makes the diagnosis and separation of the genus based on a single character almost impossible (Gojšina et al. 2025). With the increase of the species number in the family, it became clear that the generic boundaries between *Acinolaemus* and *Clostophis* are increasingly blurry since *Acinolaemus* contained several species which shared apertural barrier morphology much more similar to some *Clostophis* species. This is why it became obvious that the taxonomic revision and clarification of the genus *Acinolaemus* is needed. In this work, *Acinolaemus* is revised and 13 new species are described. The morphological distinction between *Acinolaemus* and *Clostophis* is discussed and seven species are moved from the former to the latter (*A. carcharodon*, *A. colpodon*, *A. pyramidalis*, *A. rectus*, *A. rhamphodon*, *A. sphinctinion* and *A. stenopus*) since they do not fully match the diagnosis provided by Thompson and Upatham (1997) or they are very different from the type species (*A. ptychochilus*). The revisionary work of Gojšina et al. (2025) including the genera *Anauchen*, *Bensonella*, and *Hypselostoma* was focused on species grouping based on their similarities in shell surface sculpture and apertural barriers arrangement. The same concept is followed herein and *Acinolaemus* is subdivided into six species groups.

## Materials and methods

**Figure 1.**
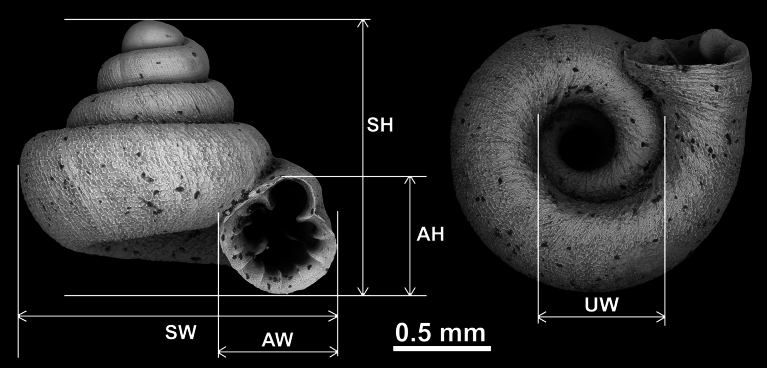
Shell measurements of *Acinolaemus* (example on *A. mueangonensis*). Abbreviations: AH = aperture height; AW = aperture width; SH = shell height; SW = shell width; UW = umbilicus width. Note that width of the umbilicus was measured only for the purpose of comparing its width to the total width of the shell (i.e., measurements of the umbilicus are not presented in millimeters).

Shells were photographed with a Nikon SMZ25 digital microscope with Nikon Nis-Elements software or directly observed without coating under a low vacuum using a Hitachi FlexSEM 1000 II scanning electron microscope in the Plant Protection Institute of the HUN-REN Centre for Agricultural Research (Martonvásár, Hungary). Shells were measured with a Nikon DS-L3 control unit. Shell characters were measured as shown in Fig. 1. Type specimens of all *Acinolaemus* species described by Thompson and Upatham (1997) were borrowed from the Florida Museum of Natural History (UF) and thoroughly examined.

### Examined characters

#### Surface of the protoconch

**Figure 2.**
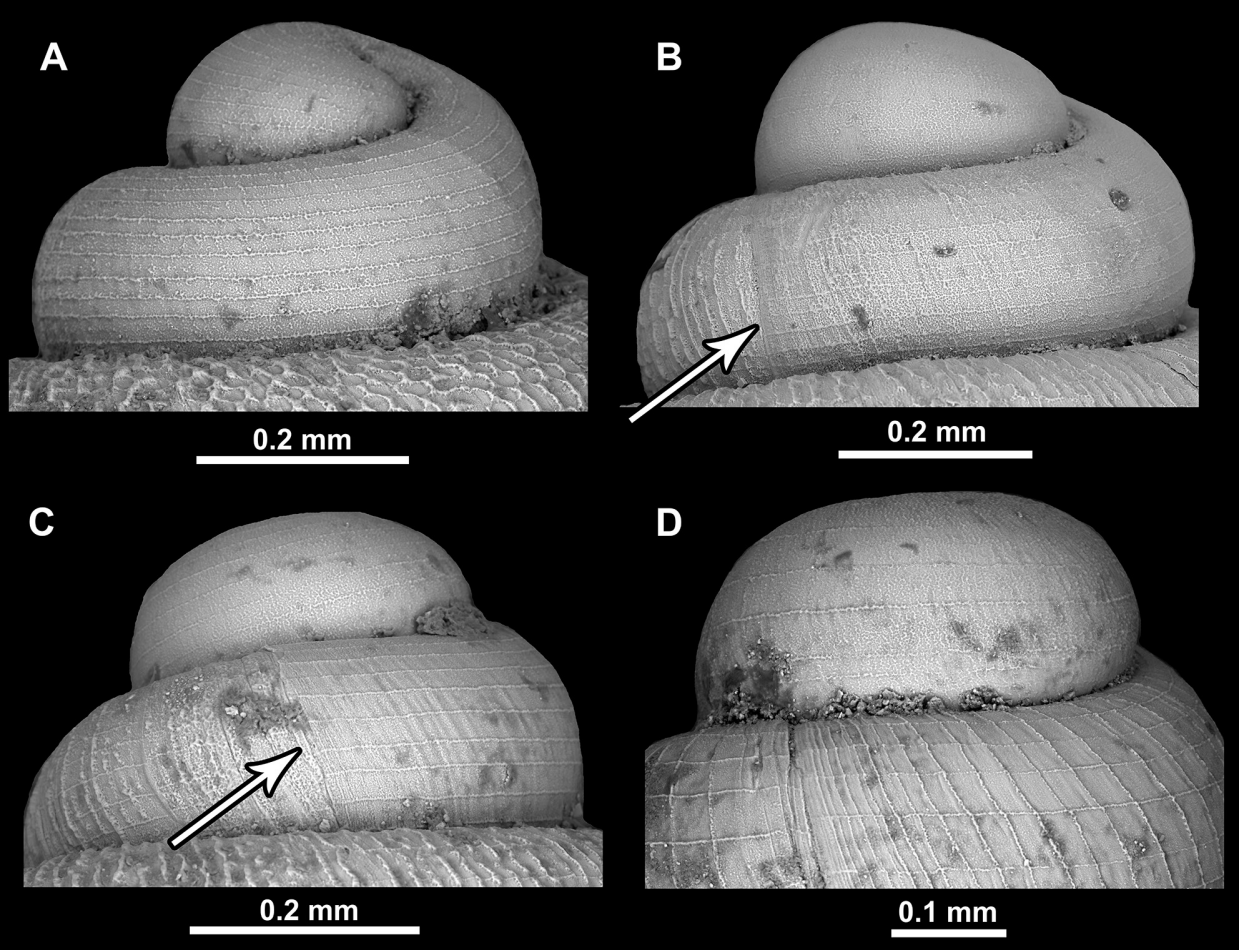
Surface of protoconch in *Acinolaemus*. **A**. Spiral striae approximately equally strong on initial and terminal portions of the protoconch (*A. asper* sp. nov.); **B**. Spiral striae slightly more clear on terminal than on initial protoconch (*A. mueangonensis*); **C**. Spiral striae much more clear on terminal than on the initial protoconch (*A. atypicus* sp. nov.); **D**. Spiral striae strong on initial protoconch whorls (*A. paucidentatus* sp. nov.). Arrows indicate the protoconch/teleoconch boundary (clear boundary present, but not visible on Fig. 2A and Fig. 2D since the images do not include that region).

The protoconch of all *Acinolaemus* species have spiral striae. In some cases, the spiral striae are stronger (clearer) and in some cases more diffuse. They can be equally strong along the entire surface of the protoconch (Fig. 2A) or clearly stronger terminally than initially (Fig. 2B, C). The protoconch/teleoconch boundary is clearly visible in all species due to the abrupt change in surface sculpture (Fig. 2B, C).

#### Surface of the teleoconch

**Figure 3.**
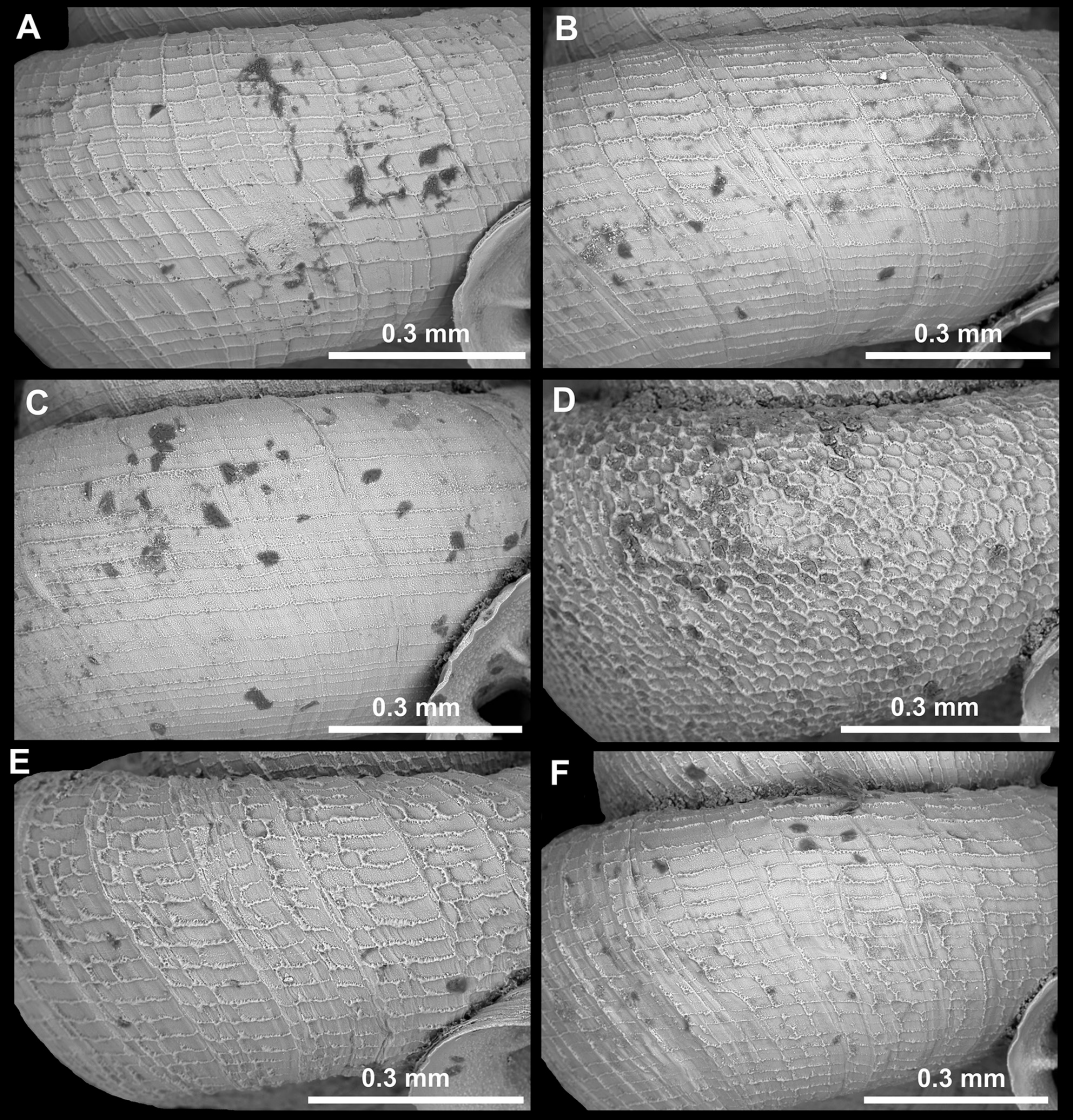
Surface of teleoconch in *Acinolaemus*. **A**. Spiral striae crossed by equally strong radial growth lines, together forming a reticulated pattern (example on *A. ptychochilus*); **B**. Spiral striae much stronger, more numerous and denser than radial growth lines (*A. profundus* sp. nov.); **C**. Spiral striae weak and low (*A. rugolabialis* sp. nov.); **D**. Honeycomb-like surface (*A. asper* sp. nov.); **E, F**. Two types of intermediary forms between honeycomb-like and clearly spirally striated surface (**E**. *A. humilis* sp. nov.; **F**. Atypical surface of *A. mueangonensis*).

Teleoconch surface sculpture is always prominent in *Acinolaemus* and always contains spiral striae. We were able to distinguish the following types of surface sculpture:

1. Spiral striae crossed by equally strong radial growth lines which together form a reticulated pattern on the surface (Fig. 3A).
2. Spiral striae much stronger, more numerous and denser than radial growth lines (Fig. 3B).
3. Spiral striae rather weak and low, crossed by even weaker radial growth lines (Fig. 3C).
4. Shell surface consisting of very numerous, polygonal fields provoking a honeycomb-like surface. This surface probably originated by numerous and irregular crossings of equally strong radial growth lines and spiral striae (Fig. 3D).

In some cases (e.g., *A. humilis* sp. nov.) the surface sculpture showed some intermediary characters between the clear honeycomb-like surface and the one with clear spiral striae crossed by radial growth lines (Fig. 3E, F). Density of sculpture can vary in each type.

#### Position and nomenclature of apertural barriers

**Figure 4.**
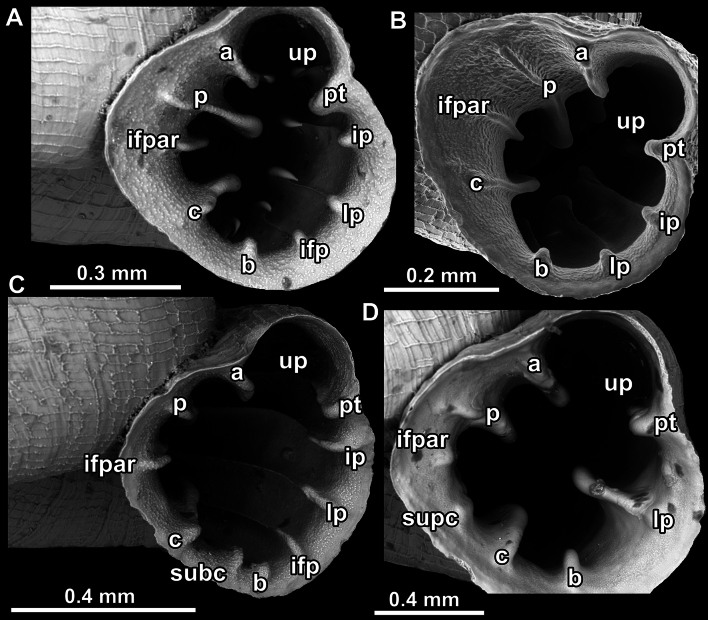
Position and nomenclature of apertural barriers in *Acinolaemus*. **A**. *A. ferox* sp. nov.; **B**. *A. cryptidentatus*; **C**. *A. mueangonensis*; **D**. *A. paucidentatus* sp. nov. Abbreviations: a = angular lamella; b = basal plica; c = columellar lamella; ifp = infrapalatal plica; ifpar = infraparietal lamella; ip = interpalatal plica; lp = lower palatal plica; pt = palatal tubercle; subc = subcolumellar lamella; supc = supracolumellar lamella; up = upper palatal plica.

Nomenclature of apertural barriers (especially those on the palatal side) follows the concept of Gojšina et al. (2025) and is shown in Fig. 4. Upper palatal plica is situated behind the palatal tubercle (sometimes appearing as above it in the standard apertural view); interpalatal plica is the first below the palatal tubercle (there can be one more interpalatal plica below the first one); lower palatal plica is approximately halfway between the basal plica and the palatal tubercle; infrapalatal plica is between the lower palatal and the basal. The main lamella on the columellar side is the columellar lamella. Sometimes, much weaker subcolumellar and supracolumellar below and above the columellar lamella can be present. The parietal side is with two (angular, parietal) or more frequently three barriers (angular, parietal, infraparietal).

#### Surface and morphology of apertural barriers and peristome

**Figure 5.**
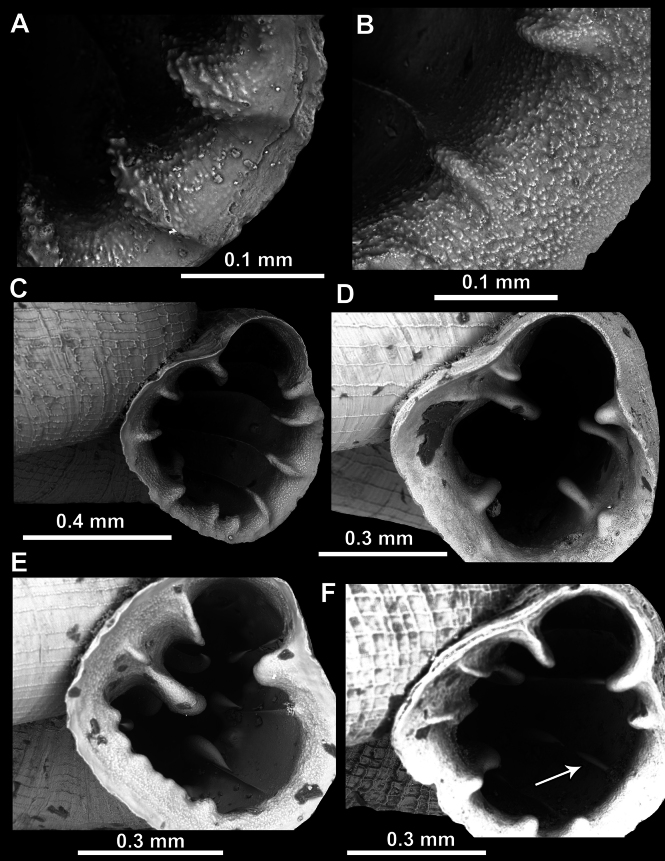
Surface (**A, B**) and morphology (**C–F**) of apertural barriers in *Acinolaemus*. **A**. Rough surface (*A. asper* sp. nov.); **B**. Finely granulated surface (*A. zed* sp. nov.); **C**. Blade-like, slender barriers (*A. mueangonensis*); **D**. Blunt, short barriers (*A. paucidentatus* sp. nov.); **E**. Hooked barriers pointing outside the aperture (*A. rugolabialis* sp. nov.); **F**. Hooked barriers pointing inside the aperture (*A. ptychochilus*, indicated by an arrow).

We were able to distinguish two types of apertural barrier and peristome surfaces:

1. Rough surface in the form of strong and raised tubercles (only *A. asper* sp. nov.) (Fig. 5A).
2. Finely granulated surface (all other species) (Fig. 5B).

Furthermore, apertural barriers (especially on the palatal side) can differ in their morphology and we distinguish the following forms:

1. Slender blade-like barriers (Fig. 5C).
2. Short blunt barriers (Fig. 5D).
3. Hooked barriers (pointing either inside or outside the aperture) (Fig. 5E, F).

#### Appearance of the last whorl

**Figure 6.**
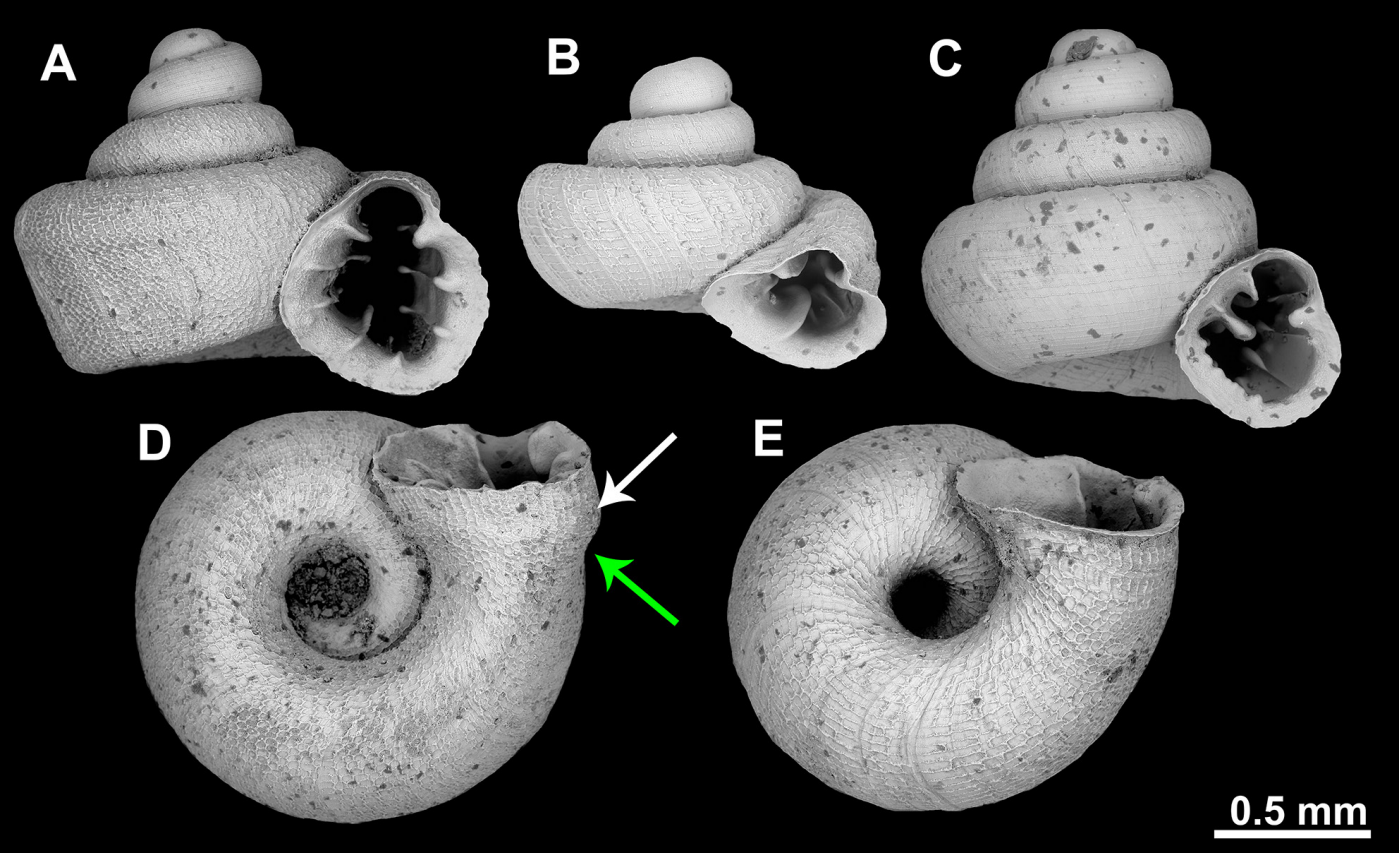
Appearance of the last whorl in *Acinolaemus*. **A, B**. Shouldered (**A**. *A. microcubus* sp. nov.; **B**. *A. humilis* sp. nov.); **C**. Rounded (*A. rugolabialis* sp. nov.); **D**. With cervical swelling (white arrow) and a constriction behind it (green arrow) (*A. mueangonensis*); **E**. Without swellings and constrictions (*A. altus* sp. nov.).

The last whorl in *Acinolaemus* is either shouldered to a different extent (Fig. 6A, B) or rarely regularly rounded (Fig. 6C). Behind the peristome, there can be a cervical swelling followed by a constriction behind it (Fig. 6D) or such swellings and constrictions can be absent (Fig. 6E).

### Abbreviations

**CUMZ** Chulalongkorn University Museum of Zoology (Bangkok, Thailand)

**HA** Collection of András Hunyadi (Budapest, Hungary)

**JG** Collection of Jozef Grego (Banská Bystrica, Slovakia)

**NHMUK** The Natural History Museum (London, UK)

**UF** Florida Museum of Natural History, University of Florida (Gainesville, USA)

## Results

### Family Hypselostomatidae Zilch, 1959

#### Genus Acinolaemus

Taxon classification

Animalia

StylommatophoraHypselostomatidae

F. G. Thompson & Upatham, 1997

5A79D2E0-FCA9-5AEE-A6F8-F89B667038A7

Acinolaemus F. G. Thompson & Upatham, 1997: 223–225.

##### Type species.

*Acinolaemus ptychochilus* F. G. Thompson & Upatham, 1997, by original designation.

##### Remarks.

We have subdivided *Acinolaemus* into six species groups based on similarities in surface sculpture and apertural barrier morphology as follows:

###### I. *Acinolaemus cryptidentatus* group – species with honeycomb-like surface and no peristomal pliculae

**Remarks**. This group is characterized by the honeycomb-like surface in combination with the lack of peristomal pliculae. Six species are included in this group: *A. altus* sp. nov., *A. atypicus* sp. nov., *A. cryptidentatus*, *A. humilis* sp. nov., *A. simplex* sp. nov. and *A. solitus* sp. nov. Of these, *A. humilis* sp. nov. has the least pronounced honeycomb-like surface with even some clearer spiral striae. However, we still include *A. humilis* sp. nov. in this species group due to the isolated presence of honeycomb-like pattern.

#### Acinolaemus altus

Taxon classification

Animalia

StylommatophoraHypselostomatidae

Gojšina, Hunyadi & Páll-Gergely sp. nov.

6ED1EB58-CB1F-597F-85FB-672DF59DAA42

https://zoobank.org/9C8833E6-A885-4FE9-8E7A-1F45C94E7779

Figs 7A, 8, 9, 17

##### Type material.

***Holotype*. Myanmar** • 1 shell; Shan State, ca 6 km east from centre of Hsihseng, right side of the road, 500 m on a side road, limestone rock; 20°8.0021'N, 97°18.0243'E: 1000 m a.s.l.; 07 Oct 2018, A. Hunyadi, K. Okubo, J. U. Otani leg; CUMZ 15424. ***Paratypes*. Myanmar** • 8 shells; same data as for holotype; coll. HA • 2 shells; Shan State, ca 4.5 km east from centre of Hsihseng, left side of the road, ca 1.7 km on a side road, limestone rock; 20°9.3587'N, 97°17.8831'E; 1140 m a.s.l.; 07 Oct 2018; A. Hunyadi, K. Okubo, J. U. Otani leg.; coll. HA • 11 shells; Shan State, Hsihseng centre NNE ca 3.6 km, limestone hill; 20°11.2485'N, 97°16.1135'E; 1040 m a.s.l.; 07 Oct 2018; A. Hunyadi, K. Okubo, J. U. Otani leg.; coll. HA.

**Figure 7.**
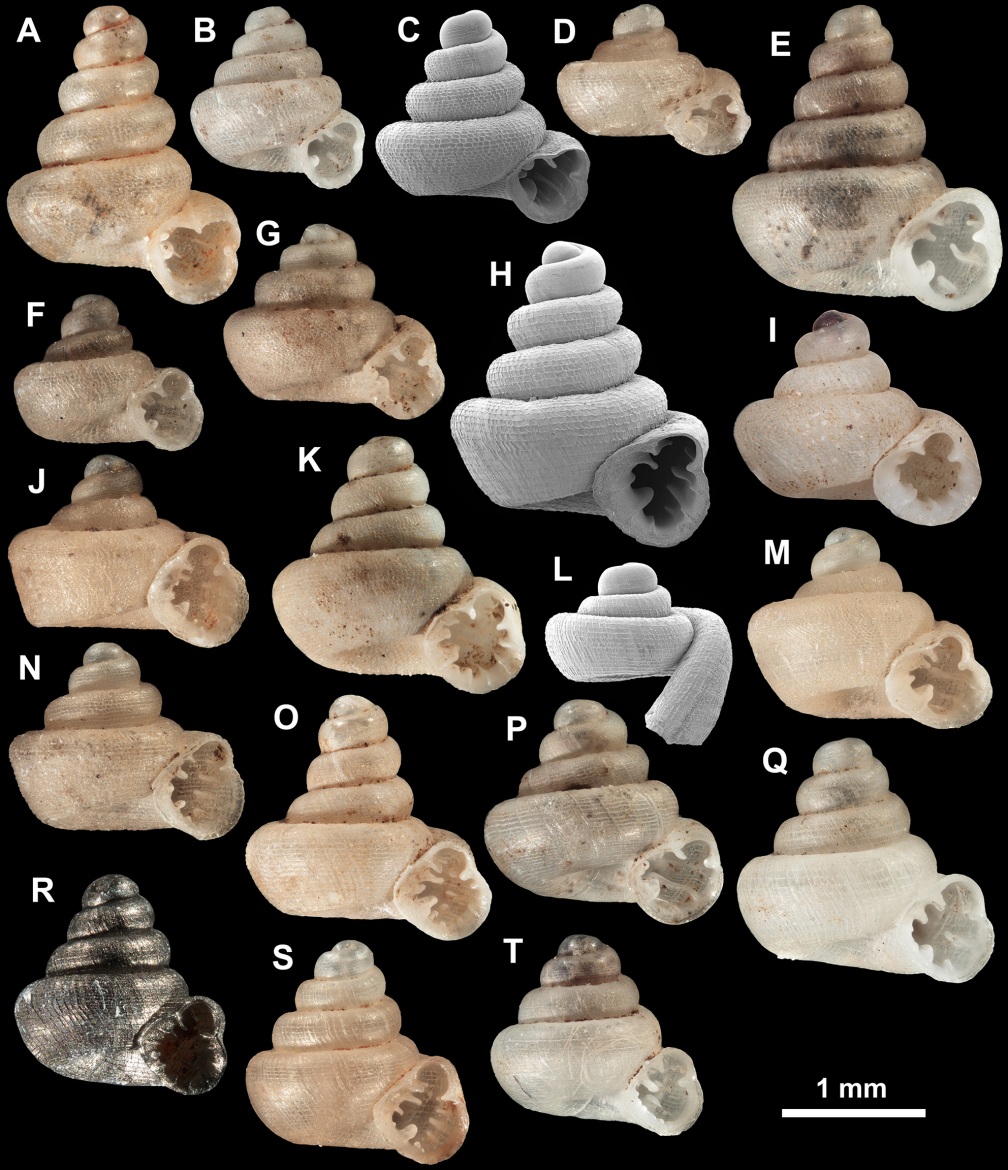
Synoptic view of all species within *Acinolaemus*. **A**. *A. altus* sp. nov., holotype (CUMZ 15424); **B**. *A. atypicus* sp. nov., holotype (UF 591353); **C**. *A. cryptidentatus*, from Tongkerd et al. (2025); **D**. *A. humilis* sp. nov., holotype (CUMZ 15425); **E**. *A. simplex* sp. nov., holotype (UF 591359); **F**. *A. solitus* sp. nov., holotype (UF 347039); **G**. *A. asper* sp. nov., holotype (UF 347324); **H**. *A. corusticorus*, holotype (CUMZ 15363.1, from Tongkerd et al. 2025); **I**. *A. dayanus* (coll. HA, from Tongkerd et al. 2024); **J**. *A. microcubus* sp. nov., holotype (CUMZ 15427); **K**. *A. mueangonensis* (UF 345719); **L**. *A. rhamphodontis*, holotype (CUMZ 14449, from Tongkerd et al. 2025); **M**. *A. zed* sp. nov., holotype (CUMZ 15456); **N**. *Acinolaemus* sp. 1 (UF 347020); **O**. *A. ferox* sp. nov., holotype (UF 591354); **P**. *A. profundus* sp. nov., holotype (UF 345958); **Q**. *A. paucidentatus* sp. nov., holotype (UF 529566); **R**. *A. ptychochilus*, holotype (UF 113502); **S**. *A. singularis* sp. nov., holotype (UF 583728); **T**. *A. rugolabialis* sp. nov., holotype (CUMZ 15457).

**Figure 8.**
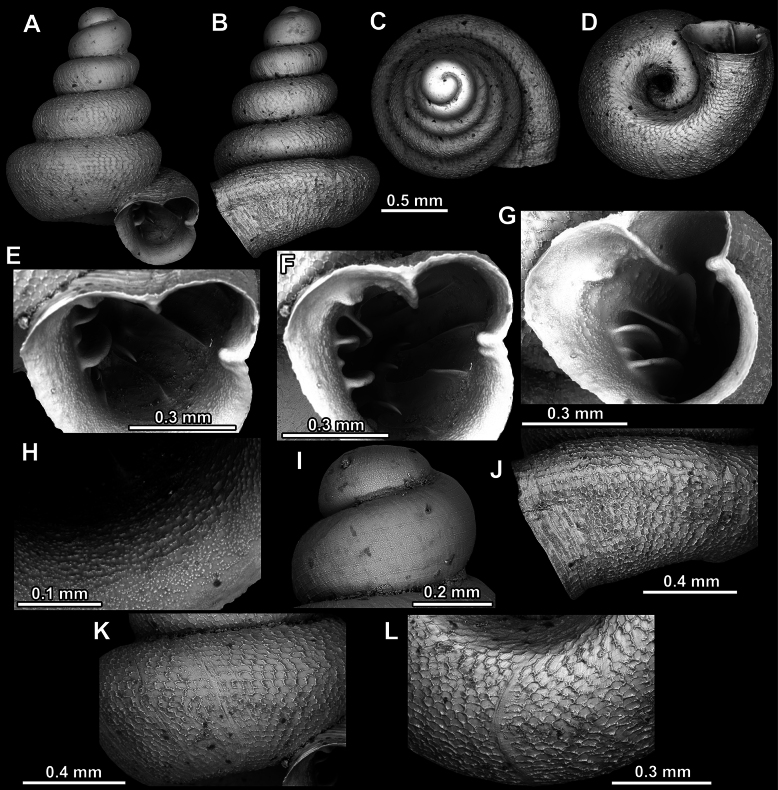
*Acinolaemus altus* sp. nov., holotype (CUMZ 15424). **A–D**. Shell; **E–G**. Enlarged views of the aperture; **H**. Enlarged view of the peristome surface; **I**. Enlarged view of the protoconch; **J**. Enlarged view of the last whorl near the aperture; **K**. Enlarged view of the last whorl surface sculpture; **L**. Enlarged view of the last whorl surface sculpture near the umbilicus.

**Figure 9.**
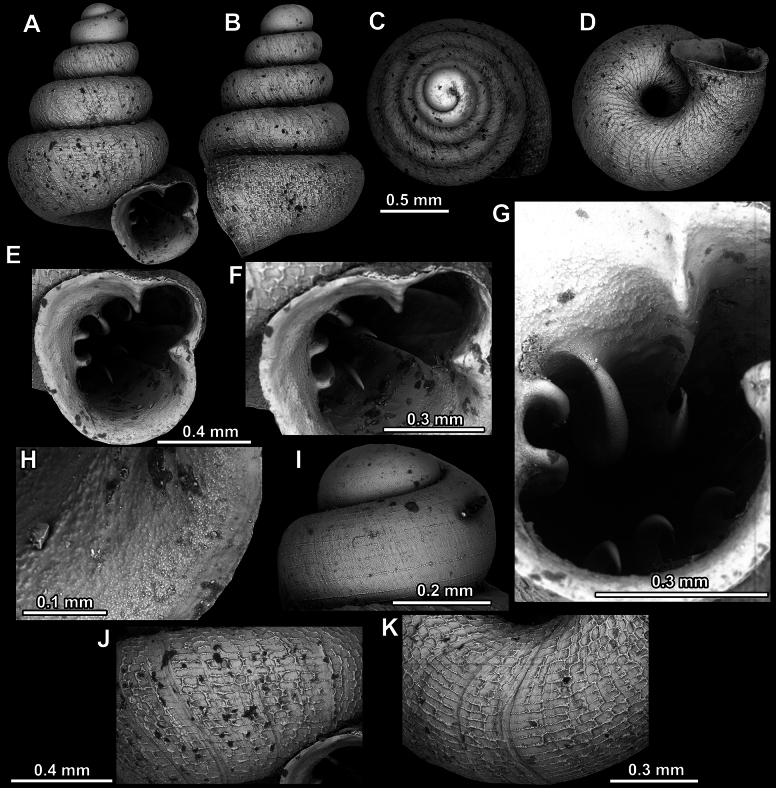
*Acinolaemus altus* sp. nov. from Shan State, Hsihseng centre NNE ca 3.6 km, paratype (coll. HA). **A–D**. Shell; **E–G**. Enlarged views of the aperture; **H**. Enlarged view of the peristome surface; **I**. Enlarged view of the protoconch; **J**. Enlarged view of the last whorl surface sculpture; **K**. Enlarged view of the last whorl surface sculpture near the umbilicus.

##### Additional material examined.

**Myanmar** • 3 shells (upper whorls broken = not paratypes); same data as for holotype; coll. HA • 3 shells (juveniles = not paratypes); Shan State, Hsihseng centre NNE ca 3.6 km, limestone hill; 20°11.2485'N, 97°16.1135'E; 1040 m a.s.l.; 07 Oct 2018; A. Hunyadi, K. Okubo, J. U. Otani leg; coll. HA.

##### Diagnosis.

Shell high concave-conical, colorless to light-yellowish. Surface sculpture honeycomb-like. Palatal plicae, basal plica, as well as the inner part of the angular lamella, are hooked.

##### Description.

Shell high, slightly concave-conical, colorless to light-yellowish, consisting of 4.75–5.25 regularly increasing, convex whorls separated by deep suture; protoconch consisting of ~ 1.5 whorls, very weakly spirally striated (~ 12 spiral striae), striae clearer terminally; boundary between protoconch and teleoconch visible due to rougher surface sculpture of teleoconch whorls; last whorl slightly shouldered, adnate to penultimate (in some specimens only peristome weakly detached) and slightly descending near aperture; surface of shell honeycomb-like, surface sculpture most prominent on last two whorls; peristome finely granulated, expanded (except around sinulus), not reflected; constriction or cervical swelling behind peristome both absent; aperture equipped with numerous (~ 12), long barriers originating deep inside aperture; parietal lamella long, curved; deeper in aperture (between angular and parietal lamellae) there can be a low additional lamella with reduced outer part closer to peristome and distant from inner part; angular lamella almost twice as long as parietal, consisting of inner, hooked part (damaged in holotype) pointing outside aperture and outer, slender part projecting towards peristome edge and reaching it; sometimes, hooked inner part is underdeveloped, seemingly blunt (although in some specimens could also be damaged); inner and outer parts of angular lamella connected by much thinner, blade-like interval; sinulus with two or three low and slender barriers; three palatal plicae present (upper, inter and lower), all with inner parts in form of elevated, sharp hooks pointing outside; outer parts of palatal plicae in form of low, slender, blade-like projections, getting thicker as getting closer to peristome; palatal plicae approximately equally developed; there can be a distinct furrow visible on last whorl behind aperture, corresponding to upper palatal plica; palatal tubercle (which can sometimes appear as double, as in holotype, see Fig. 8) distinct and located in front of upper palatal plica (and visible as slightly below it in standard view); basal plica of same morphology as palatals but lower and shorter, positioned closer to columellar lamella than to lower palatal plica; subcolumellar lamella low and blunt; columellar lamella high and blunt, not reaching expanding peristome; one or two supracolumellar lamellae present, first low (or absent), second almost as high as columellar; sinulus well-separated from rest of aperture; umbilicus not much expanded at last whorl and measuring ~ ^1^/_4_ of shell width, sometimes with shallow groove appearing at last whorl and terminating almost immediately behind the peristome.

##### Differential diagnosis.

This species can be separated from other congeners by the combination of slender shell and hooked apertural barriers (angular, basal and the palatal plicae). See under *A. cryptidentatus*.

##### Measurements (in mm, n = 5).

SH = 1.77–1.99; SW = 1.56–1.72; AH = 0.58–0.66; AW = 0.63–0.69.

##### Distribution.

This species is known from three sampling sites in southern Shan State, Myanmar.

##### Etymology.

This species is named after for altus = high in Latin, in reference to its high and narrow shell.

##### Remarks.

Last whorl can be more or less high (e.g., lower in the holotype, Fig. 8, than in the paratype, Fig. 9), which is regarded as intraspecific variability.

#### Acinolaemus atypicus

Taxon classification

Animalia

StylommatophoraHypselostomatidae

Gojšina, Tongkerd & Páll-Gergely sp. nov.

F11A7C15-D5BE-5CAF-9691-D07D18BFC219

https://zoobank.org/0744B5BC-D195-4A5B-B601-C1D1F51806E7

Figs 7B, 10, 11, 17

##### Type material.

***Holotype*. Thailand** • 1 shell; Lampang Province, limestone ridge, Ban Pang La; 18°35.1167'N, 99°50.5333'E; 400 m a.s.l., 14 May 1988; F. G. Thompson leg.; locality code FGT-4438; UF 591353. ***Paratypes*. Thailand** • 2 shells; Lampang Province, limestone dome at pass 11 km SSW of Ban Pang La; 18°27.9667'N, 99°48.3833'E; 610 m a.s.l.; 14 May 1988; F. G. Thompson leg.; sample code FGT-4441; UF 591358 • 3 shells; Lampang Province, limestone knoll 1 km NE of Ban Pang La; 18°33.25'N, 99°52.1833'E; 400 m a.s.l.; 14 May 1988; F. G. Thompson leg.; locality code FGT-4436; UF 591352 • 1 shell; Lampang Province, 6 km SW Mae Pak, limestone hill; 17°57.8833'N, 98°51.1167'E (incorrect coordinates, provided on the original label); 600 m a.s.l.; 13 Jun 1987; F. G. Thompson leg.; locality code FGT-4328; UF 593495.

**Figure 10.**
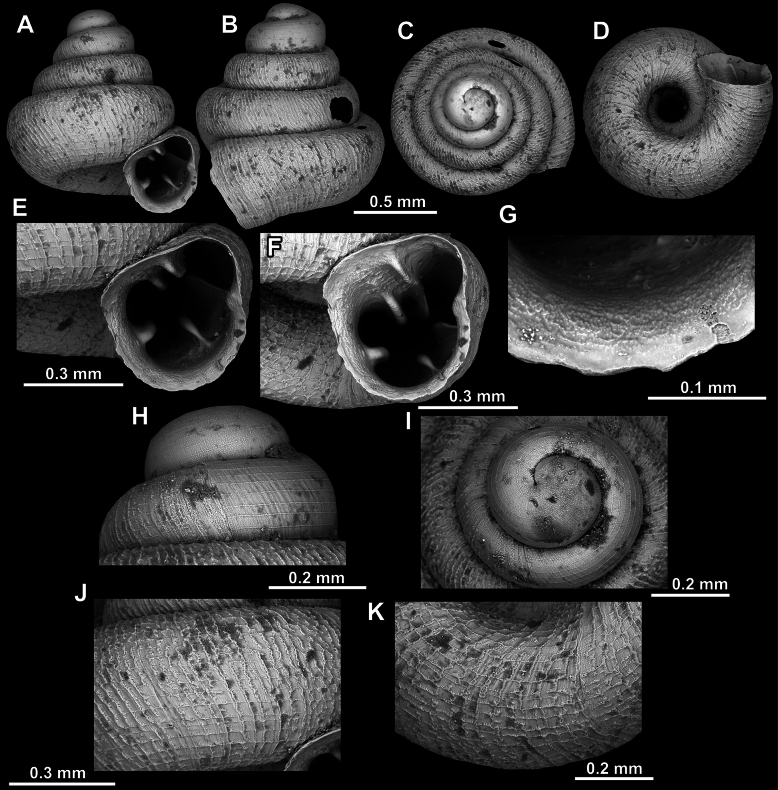
*Acinolaemus atypicus* sp. nov., holotype (UF 591353). **A–D**. Shell; **E, F**. Enlarged views of the aperture; **G**. Enlarged view of the peristome surface; **H, I**. Enlarged views of the protoconch; **J**. Enlarged view of the last whorl surface sculpture; **K**. Enlarged view of the last whorl surface sculpture near the umbilicus.

**Figure 11.**
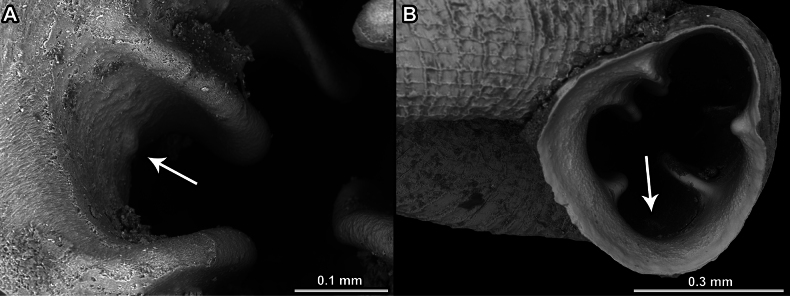
Apertural barriers variability in *A. atypicus* sp. nov. **A**. Presence of a weak infraparietal lamella (paratype, UF 593495; white arrow); **B**. Presence of a weak basal plica (paratype, UF 591358; white arrow).

##### Additional material examined.

• 1 shell (damaged, not paratype); same data as for holotype; UF 593494.

##### Diagnosis.

Shell concave-conical, colorless, with honeycomb-like surface pattern mixed with more regular rectangular fields. Two palatal plicae present, with a distinct palatal tubercle. Columellar lamella wide. Umbilicus measuring ~ ^1^/_4_ of the shell width.

##### Description.

Shell concave-conical, colorless and consisting of 4–4.25 regularly increasing, convex whorls; protoconch initially weakly spirally striated, terminally with ten stronger spiral striae; boundary between protoconch and teleoconch clearly visible due to abrupt change of surface sculpture (appearance of strong radial growth lines); last whorl adnate to penultimate, rounded to slightly shouldered, weakly descending immediately near aperture; surface sculpture of teleoconch honeycomb-like, also with some clearly rectangular fields; peristome finely granulated, expanded (especially in direction below from palatal tubercle, towards parietal wall), not reflected; cervical swelling and constriction behind peristome both absent; aperture equipped with 5–7 barriers (parietal, angular, upper palatal, lower palatal, basal, columellar, and infraparietal); parietal lamella moderately thick, not very high, situated deep and directed towards palatal side of aperture; angular lamella longer, higher and thinner than parietal, reaching expanding peristome; angular lamella consists of two parts (higher inner and lower outer) separated by thinner and less elevated, blunt interval; two palatal plicae (upper and lower) not very slender and of similar appearance (upper palatal being thinner than lower); upper palatal plica situated deeper in aperture than lower; relatively weak palatal tubercle sits in front of upper palatal plica and visible as slightly below it in standard view; basal plica barely visible or even absent, situated halfway between lower palatal plica and columellar lamella; columellar lamella usually strongest (widest) barrier in aperture, high and not very long; infraparietal lamella very weak (low and short), in form of a small tubercle-like swelling, sometimes absent; sinulus small, distinctly separated from rest of aperture; umbilicus not much expanded at last whorl and measuring between ^1^/_3_ and ^1^/_4_ of shell width.

##### Differential diagnosis.

This species has a combination of a honeycomb-like surface sculpture and a small number of barriers which makes it distinguishable from all *Acinolaemus* and *Clostophis* species. See under *A. simplex* sp. nov.

##### Measurements (n = 3, in mm).

SH = 1.16–1.18; SW = 1.17–1.2; AH = 0.49–0.5; AW = 0.45–0.47.

##### Distribution.

This species is known from Lampang Province, Thailand.

##### Etymology.

This species is named for its apertural barrier arrangement, which is generally atypical for *Acinolaemus*.

##### Remarks.

In some specimens (e.g., UF 591358, see Fig. 11B), the columellar lamella is weak so that the lower palatal plica is the strongest apertural barrier. In the same specimen, the infraparietal lamella was absent and the basal was exceptionally weak.

#### Acinolaemus cryptidentatus

Taxon classification

Animalia

StylommatophoraHypselostomatidae

Changlom, Chan-ard & Dumrongrojwattana, 2019

32596C4A-F33A-50E6-9B5E-7601DEB06686

Figs 7C, 12, 17

Acinolaemus cryptidentatus Changlom, Chan-ard & Dumrongrojwattana in Changlom et al., 2019: 158–159, fig. 2.
Acinolaemus cryptidentatus — Dumrongrojwattana and Wongkamhaeng 2024: 321, 323, 330, fig. 7, 13, table 3; Tongkerd et al. 2024: 164, fig. 2B; Tongkerd et al. 2025: 52–53, table 4.

##### Type locality.

“Tham Wua (Wua Cave), Mueang District, Mae Hong Son Province, 19°31.77'N, 98°04.92'E” (Thailand).

**Figure 12.**
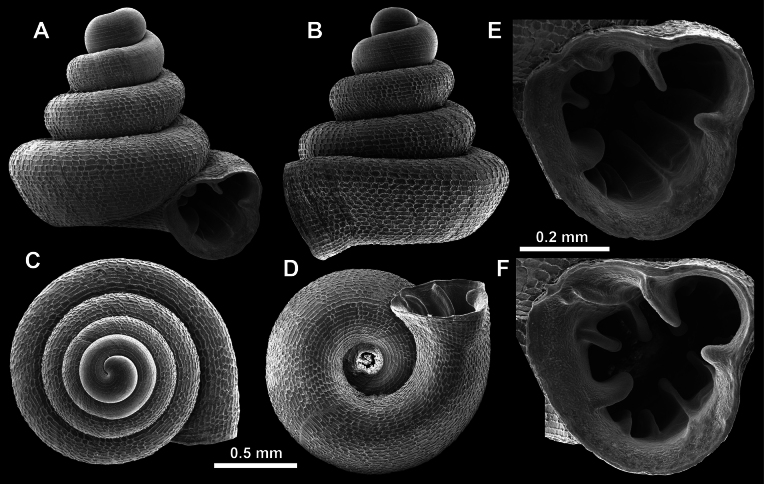
*Acinolaemus cryptidentatus* (from Tongkerd et al. 2025). **A–D**. Shell; **E, F**. Enlarged views of the aperture.

##### Material examined.

**Myanmar** • 12 shells; Shan State, 13.5 km from centre of Kalaw, Myinmati Taung; 20°35.4264'N, 96°36.7938'E; 1350 m a.s.l.; 03 Oct 2018; A. Hunyadi, K. Okubo & J. U. Otani leg.; coll. HA • 7 shells; Shan State, WSW Taunggyi, Montawa Cave; 20°45.282'N, 97°01.0572'E; 1260 m a.s.l.; 05 Oct 2018; A. Hunyadi, K. Okubo & J. U. Otani leg.; coll. HA • 22 shells; Shan State, ca 6 km from center of Hsihseng, right side of the road, 500 m on a side road, limestone rock; 20°08.0021'N, 97°18.0243'E; 1000 m a.s.l.; 07 Oct 2018; A. Hunyadi, K. Okubo & J. U. Otani leg.; coll. HA • 19 shells; Mandalay, Kyaukse, Kho Nan Shin Cave Pagoda; 21°44.153'N, 96°20.229'E; 110 m a.s.l.; 17 Oct 2018; A. Hunyadi leg.; coll. HA • 12 shells; Mandalay, Patheingyi, vicinity of Shin Bodh Mell Waterfall; 21°59.281'N, 96°12.909'E; 225 m a.s.l.; 16 Oct 2018; A. Hunyadi leg.; coll. HA • 15 shells; Mandalay, vicinity of Maha Nandamu Peik Chin Myaung Cave and waterfall; 22°05.762'N, 96°37.087'E; 740 m a.s.l.; 19 Oct 2018; A. Hunyadi leg.; coll. HA • 3 shells; Shan State, ca 6 km from center of Hsihseng, right side of the road, 400 m on a side road, limestone rock; 20°07.983'N, 97°18.1448'E; 1010 m a.s.l.; 07 Oct 2018; A. Hunyadi, K. Okubo & J. U. Otani leg.; coll. HA • 15 shells; Shan State, 7.4 km from center of Hopong towards Namsang, 5 km north on road #4, Parpant Cave; 20°50.9628'N, 97°14.2674'E; 1170 m a.s.l.; 06 Oct 2018; A. Hunyadi, K. Okubo & J. U. Otani leg.; coll. HA • 40 shells; Shan State, ca 16 km from center of Taunggyi towards Hopong, 1.5 km north on road #4, Shwe Pyi Aunchonda Monastery; 20°47.2634'N, 97°08.2394'E; 1110 m a.s.l.; 08 Oct 2018; A. Hunyadi, K. Okubo & J. U. Otani leg.; coll. HA • 3 shells; Shan State, ca 3.6 km NNE from center of Hsihseng, limestone rock; 20°11.2485'N, 97°16.1135'E; 1040 m a.s.l.; 07 Oct 2018; A. Hunyadi, K. Okubo & J. U. Otani leg.; coll. HA • 4 shells; Shan State, Hopong, Sam Phu, cave Ae-5 at ridge above village Ho Hwe; 20°41.103'N, 97°16.198'E; 12 Feb 2019; J. Grego leg.; coll. JG • 45 shells; Shan State, 5.8 km from the center of Hopong towards Namsang, left side of the road no. 4, Hopong Spring Cave; 20.81713°N, 97.22448°E; 1110 m a.s.l.; 06 Oct 2018; A. Hunyadi, K. Okubo, J. U. Otani leg.; coll. HA.

**Thailand** • 14 shells; Mae Hong Son Province, 34.0 km NW of Pai, Ban Nam Rin village; 19°27'N, 98°20'E; 940 m a.s.l.; 18 Mar 1988; K. Auffenberg leg.; locality code KA-0573; UF 593496 • 5 shells; Mae Hong Son Province, 35.1 km S of Mae Hong Son, 1.7 km E of Route 108; 19°05'N, 97°58'E; 850 m a.s.l.; 23 Mar 1988; K. Auffenberg leg.; locality code KA-0597; UF 591346 • 130 shells; Mae Hong Son Province, 13.9 km NW of Pai, along Road 1095; 19°23'N, 98°23'E; 780 m a.s.l.; 18 Mar 1988; K. Auffenberg leg.; sample code KA-0571; UF 591351 • 10 shells; Mae Hong Son Province, Ban Tham Lod village, 7 km N of Soppong on Road 1095; 19°34'N, 98°09'E; 790 m a.s.l.; 18 Mar 1988; K. Auffenberg leg.; locality code KA-0574; UF 345500 • 6 shells; Chiang Mai Province, NW side of Doi Pha Sam Sao, ravine with dipterocarp forest, leaf litter; 19°24.45'N, 99°02.93'E; 800 m a.s.l.; 20 May 1988; F. G. Thompson leg.; locality code FGT-4453; UF 593497 • 33 shells; Chiang Mai Province, 5 km NW of Chiang Dao, vicinity of Wat Tham Chiang Dao; 19°23.628'N, 98°55.711'E; 450 m a.s.l.; 07 Feb 2015; A. Hunyadi leg.; coll. HA.

##### Differential diagnosis.

This species differs from *A. altus* sp. nov. by the clearly less slender shell and absence of hooked apertural barriers. See also under *A. solitus* sp. nov., *A. corusticorus* and *A. mueangonensis*.

##### Distribution.

This species has a wider distribution from Mae Hong Son and Chiang Mai provinces in Thailand westwards to the Shan State in Myanmar.

##### Remarks.

Tongkerd et al. (2024) figured a specimen of *A. cryptidentatus* with a bit more slender apertural barriers that reach the peristome edge in the form of small pliculae. Even though this is not typical for *A. cryptidentatus*, no other differences could be observed which is why we agree that it most probably represents an unusual form of this species.

#### Acinolaemus humilis

Taxon classification

Animalia

StylommatophoraHypselostomatidae

Gojšina, Hunyadi & Páll-Gergely sp. nov.

418A31CF-EE17-599A-8C3A-5EECDDD92C02

https://zoobank.org/FD202DD2-39A7-44A9-A5FF-AD983C4D8FBA

Figs 7D, 13, 17

##### Type material.

***Holotype*. Myanmar** • 1 shell; Kayin State, 2.8 km southwest from Naung Lon towards – Zwegabin junction, right side of the road; 16°46.8146'N, 97°42.6965'E; 30 m a.s.l.; 12 Oct 2018; A. Hunyadi, K. Okubo, J. U. Otani leg.; CUMZ 15425. ***Paratypes*. Myanmar** • 6 shells; same data as for holotype; coll. HA.

**Figure 13.**
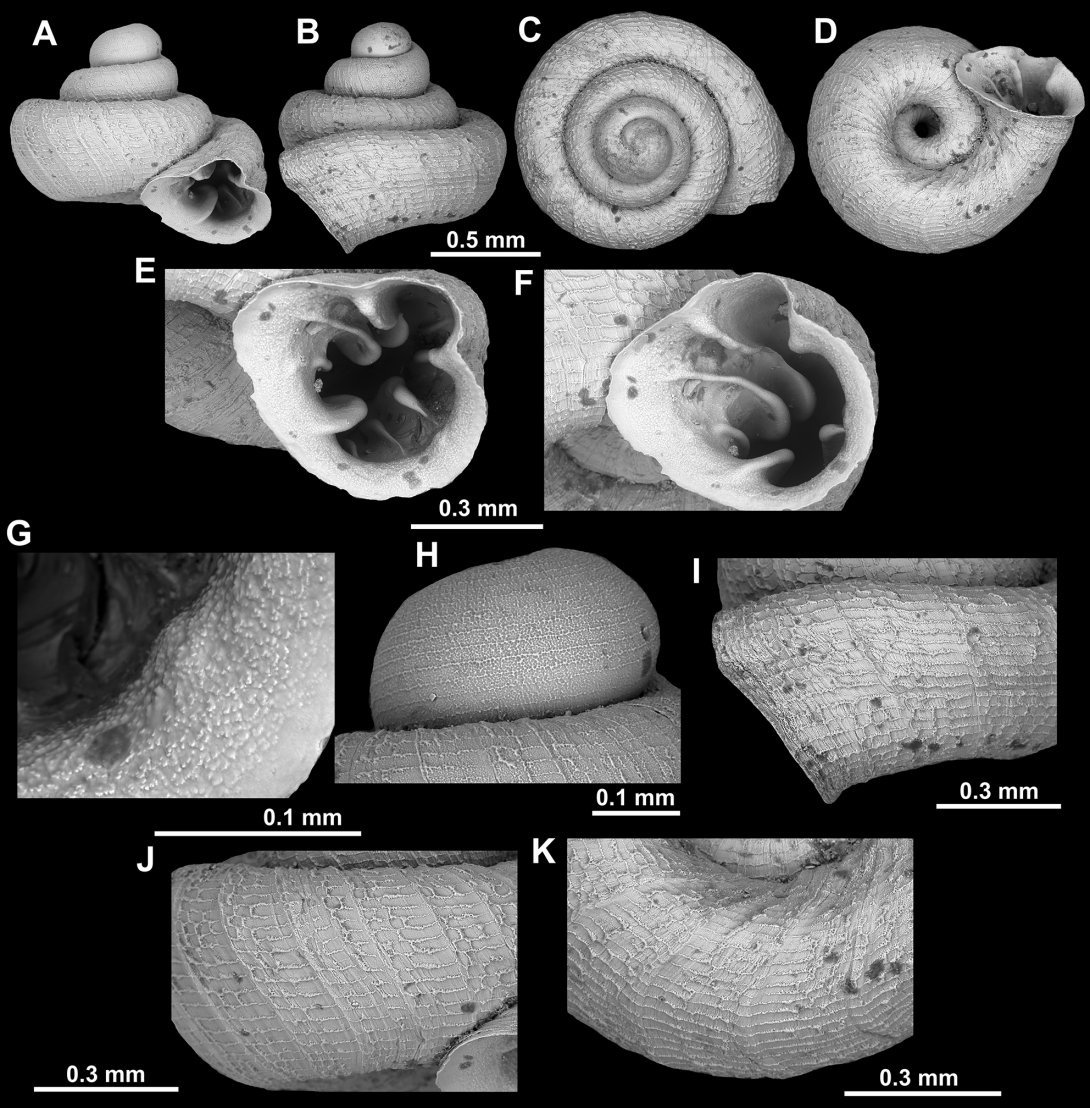
*Acinolaemus humilis* sp. nov., holotype (CUMZ 15425). **A–D**. Shell; **E, F**. Enlarged views of the aperture; **G**. Enlarged view of the peristome surface; **H**. Enlarged view of the protoconch; **I**. Enlarged view of the last whorl near the aperture; **J**. Enlarged view of the last whorl surface sculpture; **K**. Enlarged view of the last whorl surface sculpture near the umbilicus.

##### Diagnosis.

Shell depressed concave-conical, low, strongly spirally striated. Spiral striae crossed by equally strong radial growth lines. Nine strong apertural barriers. Palatal plicae and columellar lamella are especially strong. Umbilicus wide, measuring ~ ^1^/_3_ of the shell width.

##### Description.

Shell depressed concave-conical, slightly yellowish, 3–3.25 whorls separated by deep suture; protoconch of ~ 1.25 whorls, with ~ 15 spiral striae; boundary between protoconch and teleoconch discernible due to strong radial growth lines; last whorl strongly enlarged, shouldered, distinctly spirally striated, around 20 spiral striae visible in standard view; spiral striae crossed by rather randomly positioned radial growth lines which are equally strong as spiral striae and form rectangular to polygonal fields which produce honeycomb surface pattern; honeycomb-like fields appear in combination with distinct spiral striae making surface sculpture intermediary between two types; last whorl slightly descending near aperture; peristome finely granulated, strongly expanded, not reflected; slight indications of cervical swelling and constriction present behind peristome; aperture equipped with nine barriers (parietal, angular, suprapalatal, upper palatal, interpalatal, lower palatal, basal, columellar, and infraparietal); angular and parietal lamellae developed to same extent, both high, curved and long; angular lamella separated into shorter (outer) and longer (inner) parts by clear blade-like, thin interval; angular lamella reaching peristome edge, parietal almost reaching; main palatal plicae (upper, inter and lower) all long and high; inner portion of upper palatal plica high and lowering towards peristome; interpalatal plica stronger (broader and higher) than both upper and lower palatal, highest in middle portion; lower palatal plica of similar morphology as interpalatal but approximately as high and broad as upper palatal; one lower suprapalatal plica situated immediately above upper palatal; none of palatal plicae reach peristome edge; palatal tubercle strong, situated in front of upper palatal plica and appears slightly below it in standard view; basal plica low and short, situated approximately halfway between lower palatal plica and columellar lamella; latter is widest in aperture, oblique and almost reaching peristome edge; infraparietal lamella low and short, positioned halfway between columellar and parietal lamellae; sinulus small and well-separated from remainder of aperture; umbilicus wide, significantly wider towards last whorl and measuring ~ ^1^/_3_ of shell width; very shallow umbilical groove present, visible approximately at last quarter of last whorl.

##### Differential diagnosis.

See under *A. dayanus* and *A. zed* sp. nov.

##### Measurements (in mm, n = 5).

SH = 0.84–0.99; SW = 1.25–1.33; AH = 0.49–0.52; AW = 0.52–0.61.

##### Distribution.

This species is known only from the type locality.

##### Etymology.

This species’ epithet refers to its low, modest shell.

#### Acinolaemus simplex

Taxon classification

Animalia

StylommatophoraHypselostomatidae

Gojšina, Tongkerd & Páll-Gergely sp. nov.

3032F204-9E31-5C73-8F24-74294AEC2DC2

https://zoobank.org/AE768B3F-589E-4E7B-A87F-D457FADB138A

Figs 7E, 14, 17

##### Type material.

***Holotype*. Thailand** • 1 shell; Phrae Province, 11 km W of Phrae, on Road 1023, limestone knoll, leaf litter; 18°10.6833'N, 100°4.2333'E; 290 m a.s.l.; 16 May 1988; F. G. Thompson leg.; locality code FGT-4447; UF 591359. ***Paratypes*. Thailand** • 13 shells; same data as for holotype; UF 593498 • 1 shell; same data as for holotype; CUMZ 15426.

**Figure 14.**
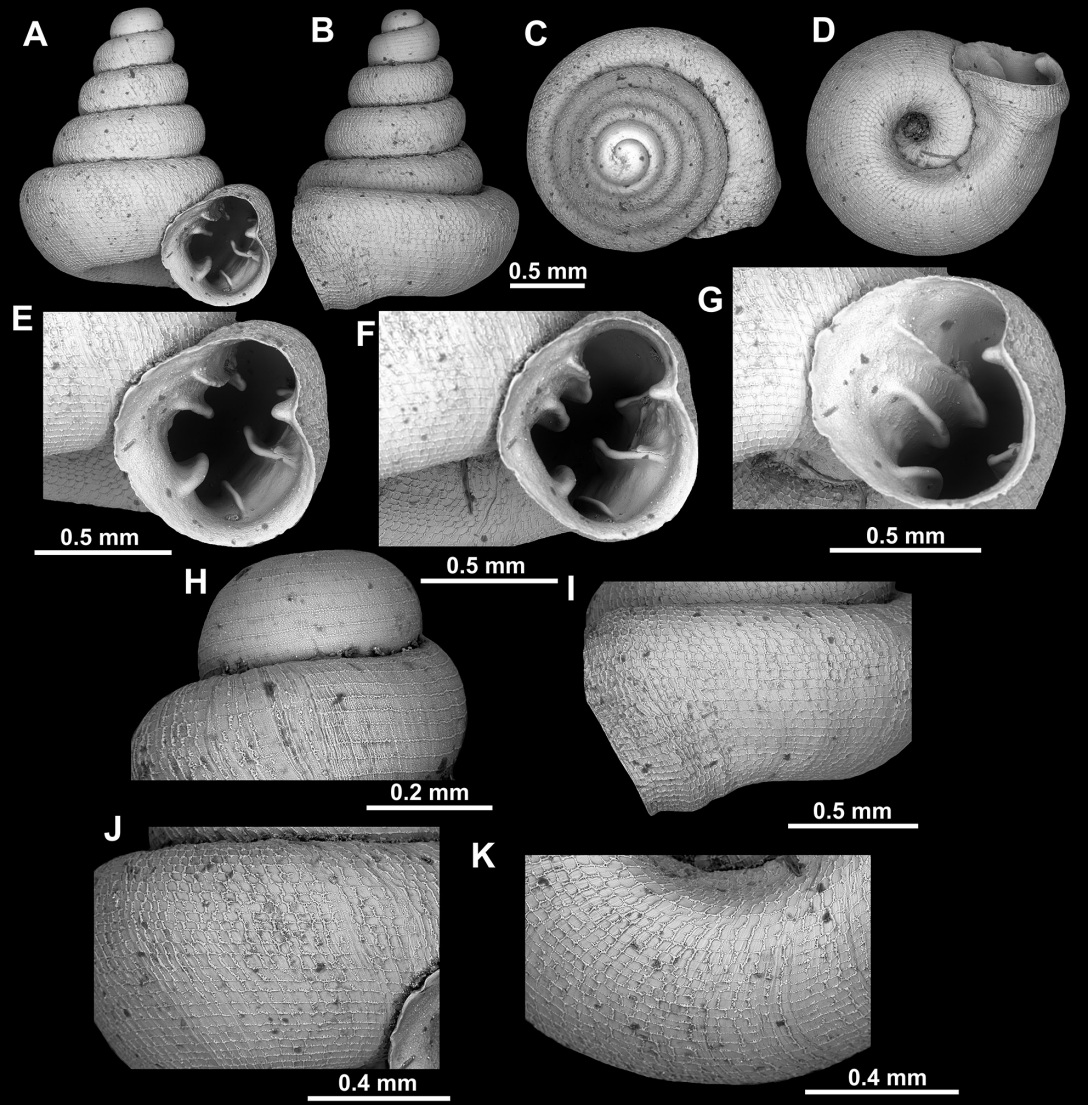
*Acinolaemus simplex* sp. nov., holotype (UF 591359). **A–D**. Shell; **E–G**. Enlarged views of the aperture; **H**. Enlarged view of the protoconch; **I**. Enlarged view of the last whorl near the aperture; **J**. Enlarged view of the last whorl surface sculpture; **K**. Enlarged view of the last whorl surface sculpture near the umbilicus.

##### Additional material examined.

**Thailand** • 1 shell (juvenile = not paratype); same data as for holotype; UF 593499.

##### Diagnosis.

Shell concave-conical, colorless, last whorl weakly shouldered. Shell surface sculpture honeycomb-like. Six apertural barriers (angular, parietal, upper palatal, lower palatal, basal and columellar).

##### Description.

Shell concave-conical, colorless and consisting of 5–5.25 regularly increasing, convex whorls; protoconch spirally striated (approximately 11 spiral striae, clearer terminally) and consisting of 1.5 whorls; boundary between protoconch and teleoconch clearly discernible due to abrupt change of surface sculpture in form of stronger radial growth lines; last whorl weakly shouldered, adnate to penultimate whorl, slightly descending near aperture; surface sculpture honeycomb-like; peristome finely granulated, expanded (except around sinulus), not reflected; cervical swelling distinct, situated immediately behind peristome, and with clear constriction behind it; aperture equipped with six barriers (parietal, angular, upper palatal, lower palatal, basal and columellar); parietal lamella not very high, not reaching peristome; angular lamella longer and thinner than parietal, almost reaching expanding peristome; angular lamella consists of two parts, inner (higher) and outer (lower); two palatal plicae (upper and lower) relatively high, not very slender and of similar appearance (upper palatal being slightly lower and thinner than lower palatal); weak to distinct palatal tubercle sits in front of upper palatal plica and appears slightly below it in standard view; basal plica slender, more than two times lower than lower palatal, situated halfway between lower palatal plica and columellar lamella; columellar lamella usually widest barrier in aperture, oblique, high and not very long, not reaching peristome; sinulus small, distinctly separated from remainder of aperture; umbilicus relatively narrow initially, expanding at last whorl and measuring between ^1^/_3_ and ^1^/_4_ of shell width and with shallow umbilical groove; indistinct periumbilical keel visible only near cervical constriction.

##### Differential diagnosis.

In comparison to *A. atypicus* sp. nov., *Acinolaemus simplex* sp. nov. has a slenderer shell with more whorls, strong basal plica, narrower umbilicus with a periumbilical keel as well as cervical swelling and a constriction. See also under *A. paucidentatus* sp. nov.

##### Measurements (n = 5, in mm).

SH = 1.86–1.99; SW = 1.63–1.77; AH = 0.71–0.82; AW = 0.68–0.75.

##### Distribution.

This species is known only from the type locality.

##### Etymology.

The specific epithet refers to the simple appearance of apertural barriers.

#### Acinolaemus solitus

Taxon classification

Animalia

StylommatophoraHypselostomatidae

Gojšina, Hunyadi & Páll-Gergely sp. nov.

BC74D010-8CC6-5D8B-BD8C-49C1EAF1A4CD

https://zoobank.org/BEED0692-FA29-4D3E-96B3-0F780EDF6A88

Figs 7F, 15–17

##### Type material.

***Holotype*. Thailand** • 1 shell; Tak Province, knoll 5.5 km NNW Tha Song Yang; 17°16.2167'N, 98°12.25'E; 180 m a.s.l.; 03 May 1988; F. G. Thompson leg.; locality code FGT-4407; UF 347039. ***Paratypes*. Thailand** • 2 shells; same data as for holotype; UF 593500 • 90 shells; Tak Province, 6 km NNW from Tha Song Yang, left side of road no. 105; 17°15.361'N, 98°12.654'E; 140 m a.s.l.; 15 Feb 2015; A. Hunyadi leg.; coll. HA.

**Figure 15.**
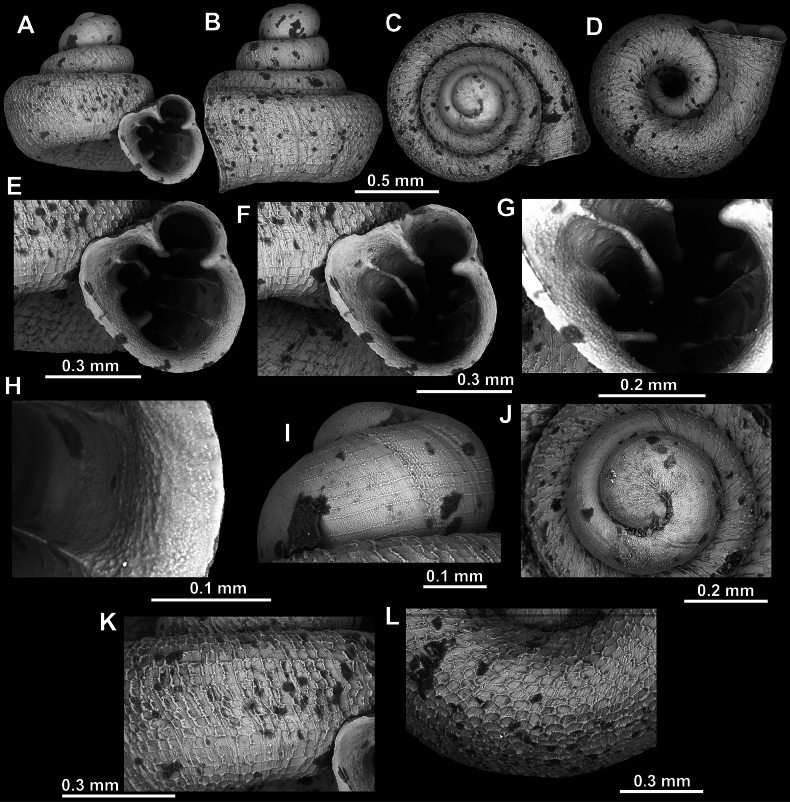
*Acinolaemus solitus* sp. nov., holotype (UF 347039). **A–D**. Shell; **E–G**. Enlarged views of the aperture; **H**. Enlarged view of the peristome surface; **I, J**. Enlarged views of the protoconch; **K**. Enlarged view of the last whorl surface sculpture; **L**. Enlarged view of the last whorl surface sculpture near the umbilicus.

**Figure 16.**
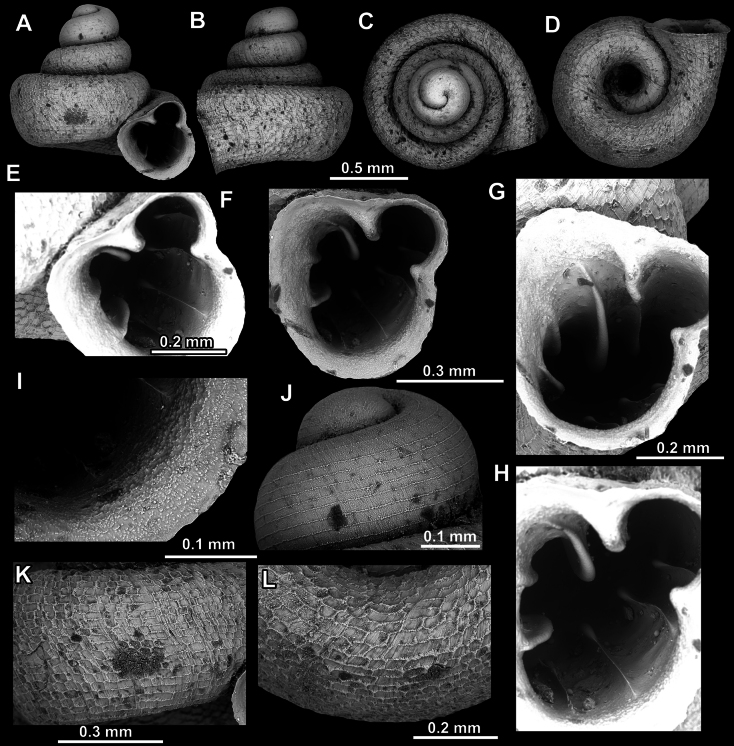
*Acinolaemus solitus* sp. nov. Tak Province, 6 km NNW from Tha Song Yang, paratype (coll. HA). **A–D**. Shell; **E–H**. Enlarged views of the aperture; **I**. Enlarged view of the peristome surface; **J**. Enlarged view of the protoconch; **K**. Enlarged view of the last whorl surface sculpture; **L**. Enlarged view of the last whorl surface sculpture near the umbilicus.

**Figure 17.**
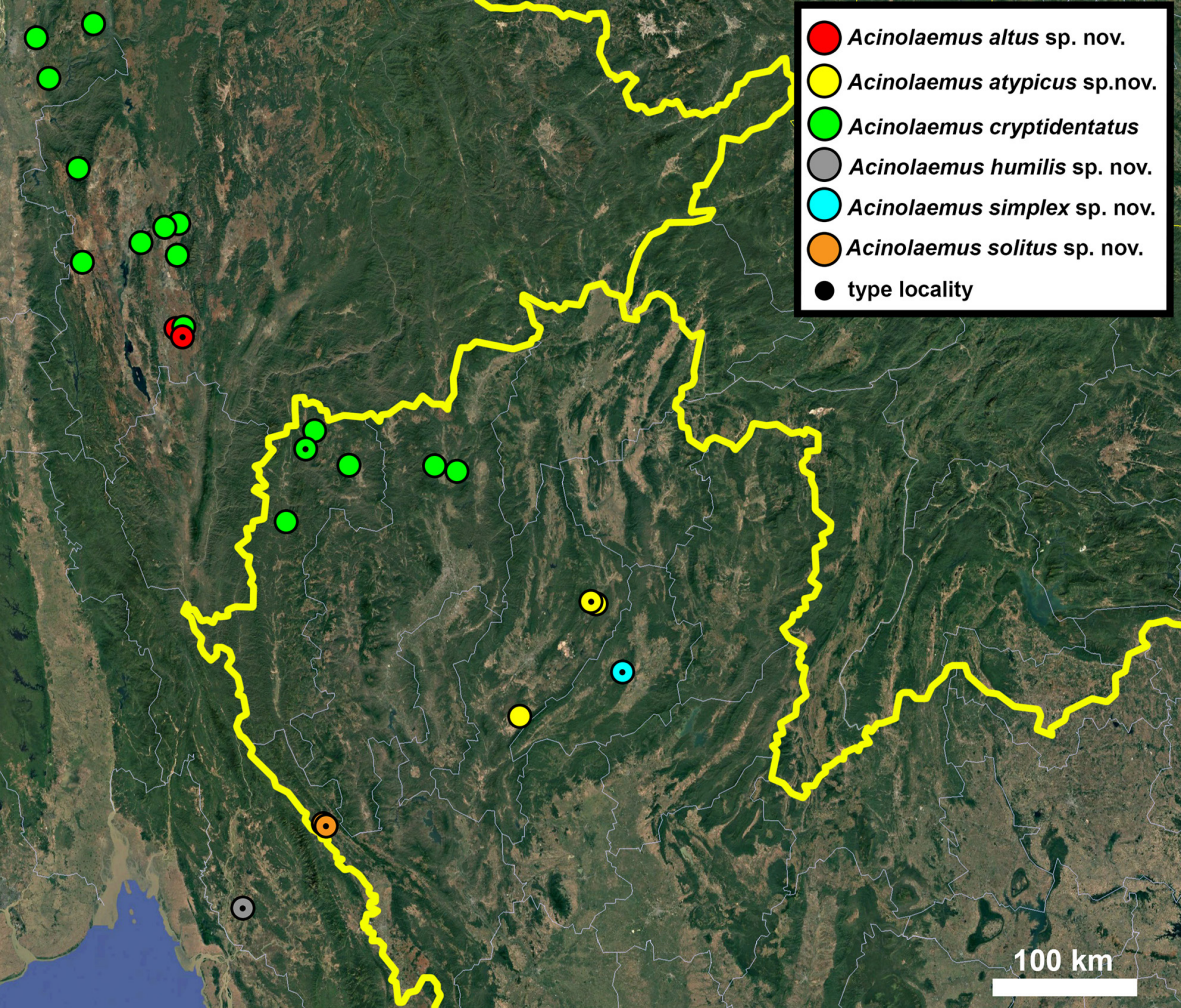
Distribution map of *Acinolaemus* species group with honeycomb-like pattern and no peristomal pliculae.

##### Additional material examined.

**Thailand** • 1 shell (broken = not paratype); same data as for holotype; UF 593501 • 20 shells (juveniles/broken = not paratypes); Tak Province, 6 km NNW from Tha Song Yang, left side of road no. 105; 17°15.361'N, 98°12.654'E; 140 m a.s.l.; 15 Feb 2015; A. Hunyadi leg.; coll. HA.

##### Diagnosis.

Shell depressed concave-conical. Last whorl shouldered, surface sculpture honeycomb-like. Aperture equipped with numerous elongated barriers. Umbilicus wide, measuring ~ ^1^/_3_ of the shell width.

##### Description.

Shell depressed concave-conical, light brownish, consisting of 3.5–3.75 convex whorls separated by deep suture; protoconch consisting of ~ 1.5 whorls and with ~ 10 clear spiral striae; boundary between protoconch and teleoconch clearly visible due to change of surface sculpture (honeycomb-like on teleoconch); last whorl shouldered, flattened below shoulder, adnate to penultimate whorl and almost parallel to shell axis in lateral view; surface sculpture honeycomb-like; peristome finely granulated, expanded (except around sinulus) but not reflected; cervical swelling and constriction both absent; aperture equipped with numerous barriers; parietal lamella long, high, slowly elevating towards its middle, not reaching peristome edge; angular lamella lower, more slender, surpassing profile of parietal lamella and reaching peristome edge; angular lamella consists of inner and outer, blunt-topped portions separated by blade-like (sharp), less elevated interval; three palatal plicae (upper, inter and lower), equally strong, higher and blunt at their inner portions and sloping towards peristome in form of slender, blade-like projections; palatal tubercle distinct, sitting in front of upper palatal plica, appearing slightly below it in standard view; basal plica of same morphology but slightly lower than palatals; columellar lamella strong (high and long), oblique (curved towards basal lip) and almost reaching peristome; infraparietal lamella moderate, two times lower than columellar, situated halfway between columellar and parietal lamella; sinulus small, distinctly separated from remainder of aperture; umbilicus wide, ~ ^1^/_3_ of shell width.

##### Differential diagnosis.

*Acinolaemus cryptidentatus* is larger, less depressed, has a narrower umbilicus and more apertural barriers (including additional small barriers inside the sinulus). See under *A. dayanus* and *A. corusticorus*.

##### Measurements (n = 3, in mm).

SH = 1.03–1.12; SW = 1.18–1.26; AH = 0.49–0.53; AW = 0.49–0.51.

##### Distribution.

This species is known from two closely situated localities in Tak Province.

##### Etymology.

The Latin-derived specific epithet solitus, meaning usual, refers to the fact that there are no peculiar, striking, or unique characters that separate this species from its similar congeners (i.e., it is unremarkable).

###### II. *Acinolaemus dayanus* group – species with honeycomb-like pattern and peristomal pliculae

**Remarks**. This species group is characterized by the honeycomb-like pattern in combination with slender apertural barriers reaching the peristome edge in the form of small pliculae. Six species belong to this group: *A. asper* sp. nov., *A. dayanus*, *A. microcubus* sp. nov., *A. mueangonensis*, *A. corusticorus*, and *A. zed* sp. nov.

#### Acinolaemus asper

Taxon classification

Animalia

StylommatophoraHypselostomatidae

Gojšina, Tongkerd & Páll-Gergely sp. nov.

1ECEA283-794B-5B23-BCB7-EFA2F72E0EF8

https://zoobank.org/80DBBBE4-63B2-49AE-BCFB-B5D0B2BD8BA4

Figs 7G, 18, 19, 30

##### Type material.

***Holotype*. Thailand** • 1 shell; Tak province, 5 km ENE Ban Huei Hin Fong; 15°46.6167'N, 98°39.8333'E; 570 m a.s.l.; 26 Apr 1988; F. G. Thompson leg.; locality code FGT-4390; UF 347324. ***Paratypes*. Thailand** • 2 shells; same data as for holotype; UF 593502 • 6 shells; Tak Province, 9.3 km ENE Ban Huei Hin Fong; 15°48.1333'N, 98°41.9'E; 660 m a.s.l.; 26 Apr 1988; F. G. Thompson leg.; locality code FGT-4387; UF 347311.

**Figure 18.**
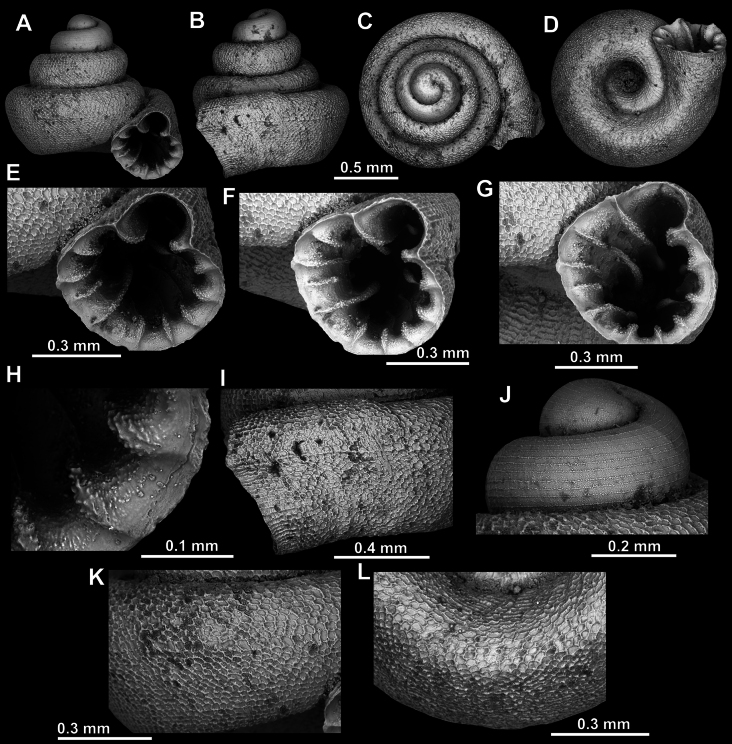
*Acinolaemus asper* sp. nov., holotype (UF 347324). **A–D**. Shell; **E–G**. Enlarged views of the aperture; **H**. Enlarged view of the peristome surface; **I**. Enlarged view of the last whorl near the aperture; **J**. Enlarged view of the protoconch; **K**. Enlarged view of the last whorl surface sculpture; **L**. Enlarged view of the last whorl surface sculpture near the umbilicus.

**Figure 19.**
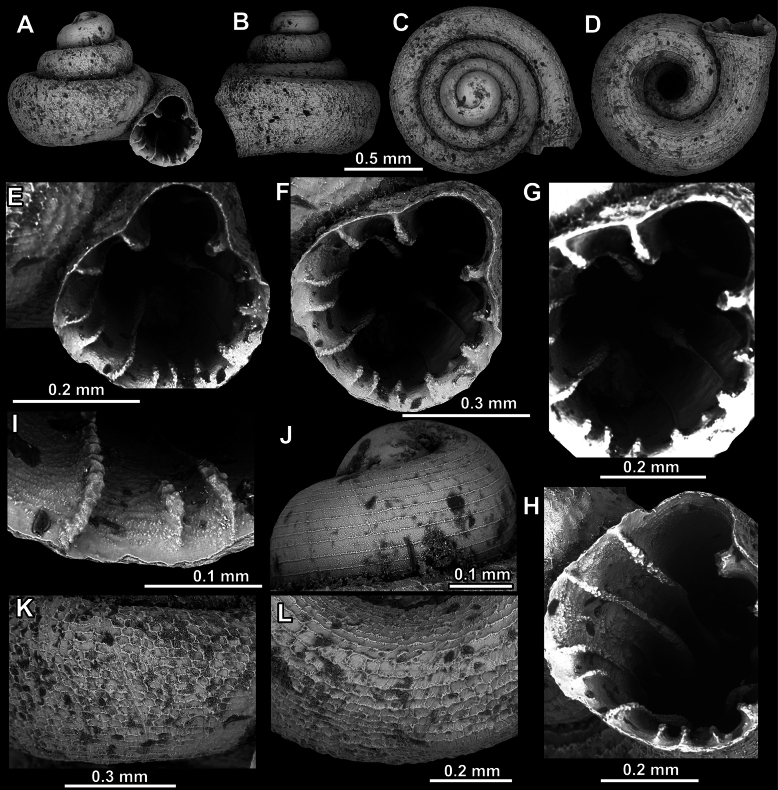
*Acinolaemus asper* sp. nov., paratype (UF 347311). **A–D**. Shell; **E–H**. Enlarged views of the aperture; **I**. Enlarged view of the peristome surface; **J**. Enlarged view of the protoconch; **K**. Enlarged view of the last whorl surface sculpture; **L**. Enlarged view of the last whorl surface sculpture near the umbilicus.

##### Additional material examined.

**Thailand** • 3 shells (damaged/juveniles = not paratypes); Tak Province, 9.3 km ENE Ban Huei Hin Fong; 15°48.1333'N, 98°41.9'E; 660 m a.s.l.; 26 Apr 1988; F. G. Thompson leg.; locality code FGT-4387; UF 593503.

##### Diagnosis.

Shell depressed, concave-conical, with honeycomb-like sculpture. Numerous apertural barriers terminating in small peristomal pliculae. All apertural barriers strongly tuberculate. Umbilicus wide, measuring ~ ^1^/_3_ of the shell width.

##### Description.

Shell depressed, concave-conical, light yellowish, consisting of 3.5–4 convex whorls separated by deep suture; protoconch consisting of ~ 1.5 whorls, spirally striated (11 distinct, equidistant spiral striae terminally); boundary between protoconch and teleoconch clearly visible due to change of surface sculpture (becoming honeycomb-like on teleoconch); last whorl weakly shouldered, adnate to penultimate and slightly descending near aperture; shell surface honeycomb-like, equally strong on whole shell; peristome expanded (except around sinulus), not reflected; cervical swelling and constriction immediately behind peristome both present but weak; aperture equipped with numerous barriers, all terminating in small peristomal pliculae; parietal lamella long, moderately high, reaching peristome; angular lamella slender, higher and blunter at its inner portion, reaching peristome; blunt topped inner and outer portions of angular lamella separated by short, blade-like and less elevated interval; all palatal plicae blunt and elevated at their inner portions, sloping and getting blade-like at outer portions; upper palatal plica higher than interpalatal; lower palatal plica higher and broader than both inter and upper palatals; distinct palatal tubercle sits in front of upper palatal, visible as slightly below it in standard view; infrapalatal plica two times weaker (lower and narrower) than lower palatal; basal plica approximately of same appearance as infrapalatal; one or two subcolumellar lamellae present, nearly as strong as infrapalatal plica; columellar lamella not blade-like, oblique, reaching peristome where it becomes quite low and slender; supracolumellar lamella present but very low, equally developed infraparietal lamella present above it, both without blade-like intervals; surface of all barriers very rough, with strong, raised tubercles; sinulus small, distinctly separated from the rest of aperture; umbilicus wide, measuring ~ ^1^/_3_ of shell width.

##### Differential diagnosis.

This species can be separated from all other congeners most clearly by the strongly tuberculate apertural barriers.

##### Measurements (n = 5, in mm).

SH = 1.03–1.29; SW = 1.34–1.46; AH = 0.48–0.56; AW = 0.42–0.54.

##### Distribution.

This species is known only from the Tak Province.

##### Etymology.

The specific epithet if from the Latin asper, meaning rough, referring to the peculiar surface of apertural barriers.

#### Acinolaemus corusticorus

Taxon classification

Animalia

StylommatophoraHypselostomatidae

Tongkerd & Panha, 2025

0E4D7EAE-D5CD-51D3-A759-593A36383CE9

Figs 7H, 20, 30

Acinolaemus corusticorus Tongkerd & Panha in Tongkerd et al., 2025: 59–62, figs 13D, E, 14, 15, table 4.

##### Type locality.

“Phra Phutthabat Doi Khao Nam, Ban Na subdistrict, Sam Ngao District, Tak Province; 17°14'56.4"N, 98°56'16.3"E” (Thailand).

**Figure 20.**
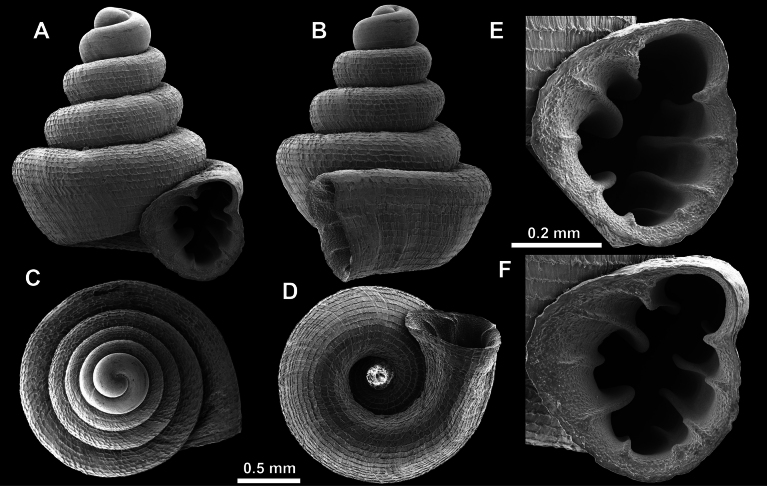
*Acinolaemus corusticorus*, holotype (CUMZ 15363.1) (from Tongkerd et al. 2025). **A–D**. Shell; **E, F**. Enlarged views of the aperture.

##### Material examined.

**Thailand** • holotype; CUMZ 15363.1.

##### Differential diagnosis.

*Acinolaemus cryptidentatus* normally has no peristomal pliculae and has more apertural barriers (including several small barriers in the sinulus). Also, the last whorl is not so strongly shouldered in *A. cryptidentatus* as it is in *A. corusticorus*. *Acinolaemus solitus* sp. nov. is smaller, has blade-like barriers and has no peristomal pliculae. See also under *A. mueangonensis*.

##### Distribution.

This species is only known from the type locality (Tongkerd et al. 2025).

#### Acinolaemus dayanus

Taxon classification

Animalia

StylommatophoraHypselostomatidae

(Stoliczka, 1871)

1F872EEF-BDEB-5917-8F68-787217883512

Figs 7I, 21, 30

Hypselostoma dayanum Stoliczka, 1871: 172, 173, pl. 7, fig. 2.
Hypselostoma dayanum — Hanley and Theobald 1876: 59, pl. 147, fig. 10; Pfeiffer 1876: 488; Pfeiffer 1880: 344.
Boysidia (?) dayana — Pilsbry 1917: 205, 206, pl. 34, figs 5, 6.
Pupa (Hypselostoma) dayana — Nevill 1878: 193.
Pupa (Hypselostoma) dayanum — Gude 1914: 300, 301.
Hypselostoma dayana — Thompson and Upatham 1997: 225.
Acinolaemus dayanum — Tongkerd et al. 2024: 162, figs 2A, 13A; Tongkerd et al. 2025: 53, table 4.

##### Type locality.

“Damotha, prope Moulmein” [Kayon Hill, Dhammasa Village, Mawlamyine District, Mon State, Myanmar].

**Figure 21.**
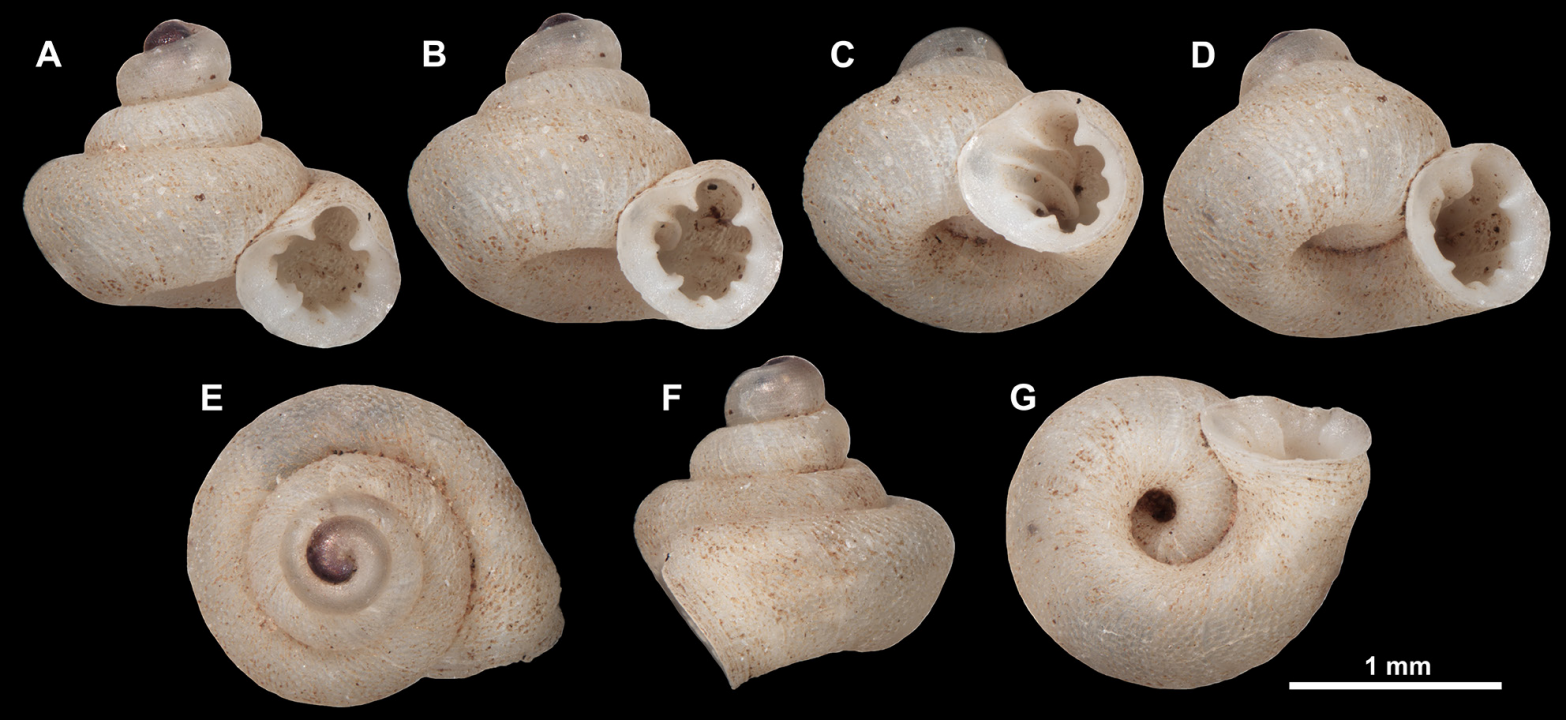
*Acinolaemus dayanus* from Mon State, Myanmar (photos **A**, **E**, **F** and **G** are from Tongkerd et al. 2024).

##### Material examined.

**Myanmar** • 11 shells; Mon State, Mawlamyine center NEE ca 26 km, Dhammasa Cave; 16°30.4029'N, 97°48.6458'E; 8 m a.s.l.; 11 Oct 2018; A. Hunyadi, K. Okubo, J. U. Otani leg.; coll. HA.

##### Differential diagnosis.

*Acinolaemus asper* sp. nov. has a wider umbilicus and more peristomal pliculae than *A. dayanus* as well as the very rough surface of the barriers. *Acinolaemus solitus* sp. nov. has a wider umbilicus, less slender apertural barriers and lacks the peristomal pliculae. This species differs from *A. humilis* sp. nov. by the more slender palatal plicae which are situated deeper in the aperture and reach the peristome edge in the form of small pliculae. Furthermore, the shell of *A. humilis* sp. nov. is lower and apertural barriers are stronger than those of *A. dayanus*. See also under *A. mueangonensis*, *A. microcubus* sp. nov., and *A. zed* sp. nov.

##### Distribution.

This species is known only from the type locality.

#### Acinolaemus microcubus

Taxon classification

Animalia

StylommatophoraHypselostomatidae

Gojšina, Hunyadi & Páll-Gergely sp. nov.

A9E42EAB-CFB3-52A1-BE3D-8AEAFD6E56C4

https://zoobank.org/68AAA091-91AF-46AA-8E1A-AF9EC23AD024

Figs 7J, 22, 30

##### Type material.

***Holotype*. Myanmar** • 1 shell; Kayin State, ca 51 km east-southeast from centre of Mudon, northeast from Htimahto, northeastern end of Kwooprai Hill; 16°2.3959'N, 97°58.2001'E; 25 m a.s.l.; 07 Oct 2018; A. Hunyadi, K. Okubo, J. U. Otani leg.; CUMZ 15427. ***Paratypes*. Myanmar** • 7 shells; same data as for holotype; coll. HA.

**Figure 22.**
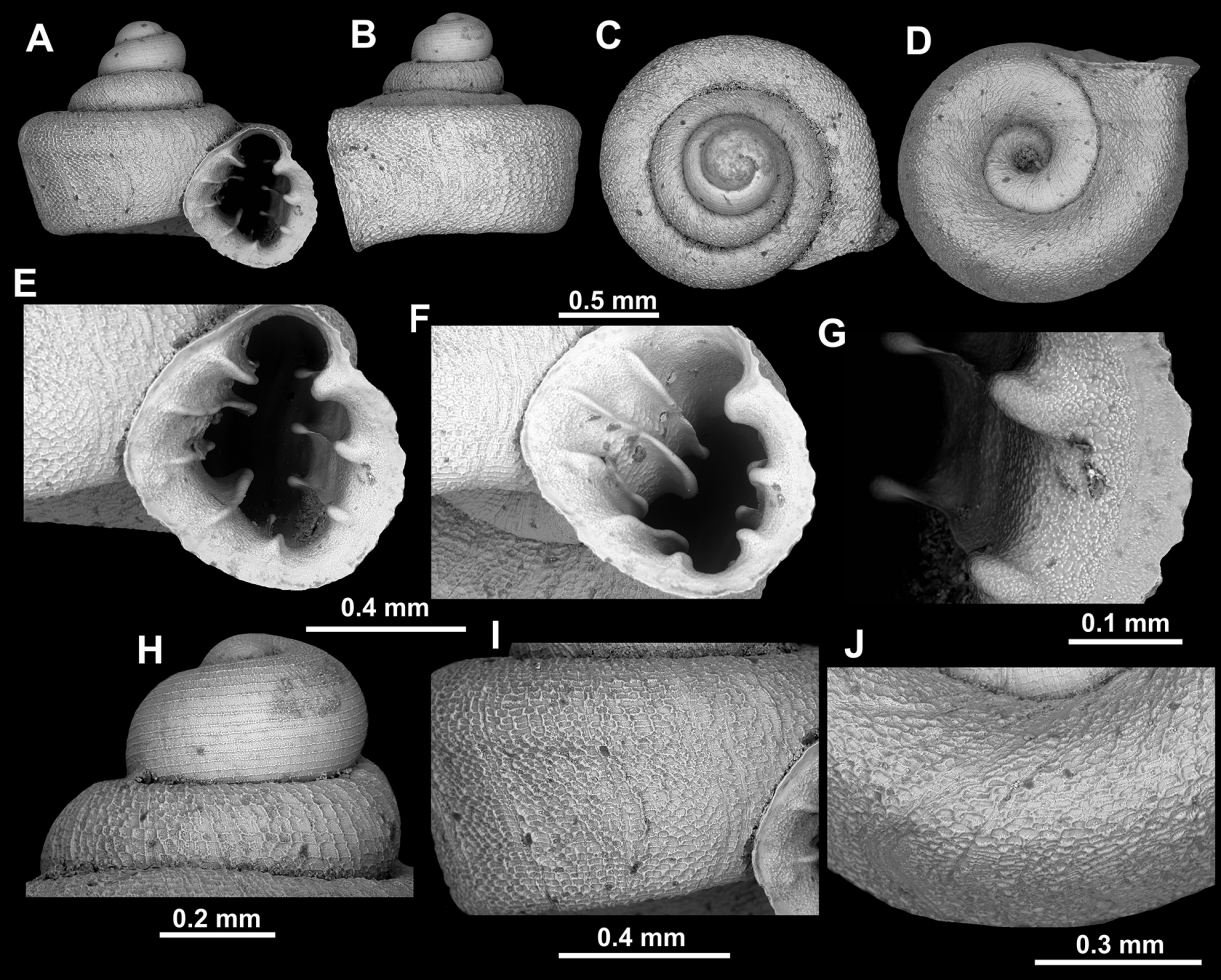
*Acinolaemus microcubus* sp. nov., holotype (CUMZ 15427). **A–D**. Shell; **E, F**. Enlarged views of the aperture; **G**. Enlarged view of the peristome surface; **H**. Enlarged view of the protoconch; **I**. Enlarged view of the last whorl surface sculpture; **J**. Enlarged view of the last whorl surface sculpture near the umbilicus.

##### Diagnosis.

Shell depressed conical, yellowish, with honeycomb-like surface sculpture. Last whorl strongly shouldered, umbilicus very wide. Aperture equipped with eight low, elongated barriers and a distinct palatal tubercle.

##### Description.

Shell depressed conical, light yellowish to brownish, consisting of 3.5–4 whorls separated by deep suture; protoconch spirally striated (approximately 15 equidistant spiral striae), consisting of ~ 1.5–1.75 whorls; boundary between protoconch and teleoconch distinct due to abrupt change of surface sculpture (distinct spiral striae on protoconch vs honeycomb-like on teleoconch); protoconch and all teleoconch whorls, except last one, are rounded, weakly convex; last whorl enlarged, distinctly shouldered; surface sculpture equally prominent on whole teleoconch; last whorl very weakly descending near aperture but apertural profile almost parallel to shell axis; peristome finely granulated, expanded (except around sinulus), not reflected: no constriction or cervical swelling behind peristome; aperture equipped with eight barriers (parietal, angular, upper palatal, interpalatal, lower palatal, basal, columellar and infraparietal); angular and parietal lamellae developed approximately to same extent, both long, parietal lamella bit higher, both almost reaching expanding peristome; parietal lamella not blade-like; angular lamella consists of two parts (inner (higher) and outer (lower)) separated by blade-like, less elevated interval; three palatal plicae (upper, inter and lower) all equally wide and long, higher and blunt at their inner portions and abruptly sloping as blade-like towards peristome; palatal tubercle distinct, situated in front of upper palatal plica and appears slightly below it in standard view; interpalatal and lower palatal plicae projecting all the way to expanding peristome and terminating in moderate, blunt pliculae below strong palatal tubercle; basal plica low, positioned closer to columellar than to lower palatal, reaching peristome similarly as described for interpalatal and lower palatal plicae, and of same morphology; columellar lamella long, blunt along entire length, oblique, almost fully reaching peristome edge; infraparietal lamella low but long, blunt, nearly reaching peristome edge; sinulus small and distinctly separated from remainder of aperture; umbilicus wide, between ^1^/_2_ and ^1^/_3_ of shell width, widening near last whorl; blunt periumbilical keel present.

##### Differential diagnosis.

This species is similar to *A. dayanus* in general shell shape and apertural barrier arrangement. However, the last whorl is much more strongly shouldered in the new species and there is neither a cervical swelling nor the constriction behind the peristome (present in *A. dayanus*). The umbilicus is also narrower in *A. dayanus* and there is no periumbilical keel. See also under *A. corusticorus* and *A. zed* sp. nov.

##### Measurements (in mm, n = 5).

SH = 1.16–1.28; SW = 1.41–1.42; AH = 0.61–0.71; AW = 0.59–0.65.

##### Distribution.

This species is known only from the type locality.

##### Etymology.

The specific epithet refers to the combination of a small and cube-shaped shell (due to the strongly shouldered last whorl).

#### Acinolaemus mueangonensis

Taxon classification

Animalia

StylommatophoraHypselostomatidae

Changlom, Chan-ard & Dumrongrojwattana, 2019

984272E2-37DF-5CC1-B8CC-C05EC1BE929C

Figs 7K, 23K–26, 30

Acinolaemus mueangonensis Changlom, Chan-ard & Dumrongrojwattana, 2019: 159–161, fig. 3A–G.
Acinolaemus *mueangon* [sic] — Dumrongrojwattana and Wongkamhaeng 2024: 321, 323, 330, fig. 7, 13, table 3.
Acinolaemus mueangonensis — Tongkerd et al. 2025: 55–59, table 4.

##### Type locality.

“Tham Mueang on [Mueang On Cave], Mae On District, Chiang Mai Province, 18°47.39'N, 99°14.43'E” (Thailand).

##### Material examined.

**Thailand** • 3 shells; Mae Hong Son Province, Mae Hong Son, road 1095; 19°25'N, 97°58'E; 390 m a.s.l.; 22 Mar 1988; K. Auffenberg leg.; locality code KA-0595B; UF 345719 • 31 shells; Mae Hong Son Province, 40 km N of Mae Hong Son; 19°27'N, 97°59'E; 550 m a.s.l.; 21 Mar 1988; K. Auffenberg leg.; sample code KA-0588; UF 345640 • 24 shells; Loei Province, Mueang Loei district, rock wall above Wat Tham Piya Thammarangsi; 17°27.8958'N, 101°51.5778'E; 390 m a.s.l.; 27 Feb 2023; A. Hunyadi, J.U. Otani leg.; coll. HA • 1 shell; Loei Province, Nong Hin district, Puan Phu subdistrict, 20.3 km southwest from the centre of Nong Hin, Pha Wai, left side of the road no. 3029; 17°2.4708'N, 101°43.6548'E; 705 m a.s.l.; 28 Feb 2023; A. Hunyadi, J. U. Otani leg.; coll. HA.

##### Differential diagnosis.

This species differs from *A. cryptidentatus* by the much more slender, blade-like apertural barriers which reach the peristome edge in small pliculae as well as the brownish coloration of the shell. This species differs from *A. corusticorus* by the brown shell as well as longer and blade-like apertural barriers.

##### Distribution.

This species is known from Chiang Mai, Mae Hong Son, Loei, and Tak provinces in Thailand.

##### Remarks.

Specimens from Tak Province reported by Tongkerd et al. (2025) share the same apertural barrier arrangement, surface sculpture, and coloration with the typical *A. mueangonensis* but have more slender shells. Since the slenderness of the shell is found to be variable within several species, the population from Tak Province is identified as *A. mueangonensis*. Some specimens from Mae Hong Son Province (UF 345640) (Fig. 25) shared the same shell shape, apertural barrier arrangement and umbilicus width with typical *A. mueangonensis* but had a less pronounced honeycomb-like surface, which is distinct only on isolated parts of the shell. Since other specimens from the same sample had a surface more similar to typical *A. mueangonensis*, we also treat the population from Mae Hong Son as *A. mueangonensis*. Specimens from Loei Province had a more depressed shell with a wider umbilicus (Fig. 26) and are therefore identified as *Acinolaemus cf. mueangonensis*.

**Figure 23.**
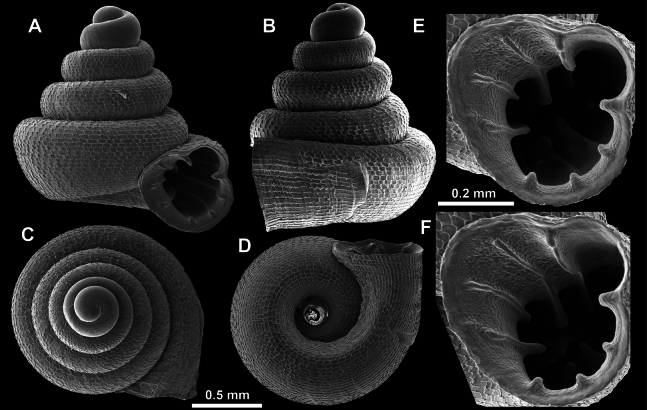
*Acinolaemus mueangonensis* (from Tongkerd et al. 2025). **A–D**. Shell; **E, F**. Enlarged views of the aperture.

**Figure 24.**
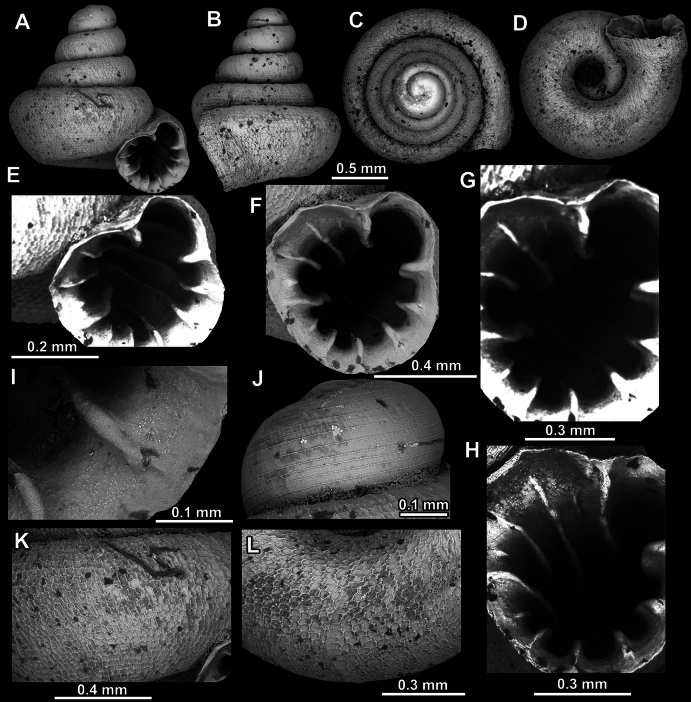
*Acinolaemus mueangonensis* from Mae Hong Son Province (UF 345719). **A–D**. Shell; **E–H**. Enlarged views of the aperture; **I**. Enlarged view of the peristome surface; **J**. Enlarged view of the protoconch; **K**. Enlarged view of the last whorl surface sculpture; **L**. Enlarged view of the last whorl surface sculpture near the umbilicus.

**Figure 25.**
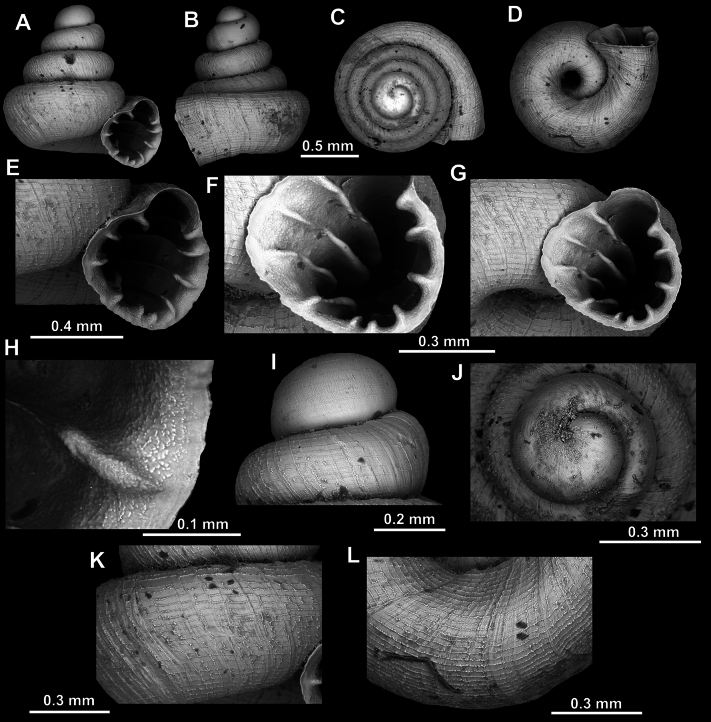
*Acinolaemus mueangonensis* from Mae Hong Son Province (UF 345640). **A–D**. Shell; **E–G**. Enlarged views of the aperture; **H**. Enlarged view of the peristome surface; **I, J**. Enlarged views of the protoconch; **K**. Enlarged view of the last whorl surface sculpture; **L**. Enlarged view of the last whorl surface sculpture near the umbilicus.

**Figure 26.**
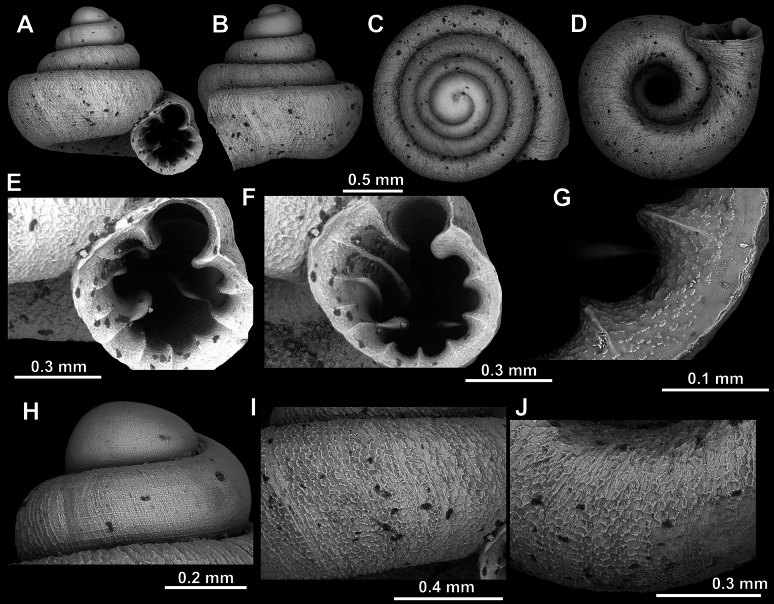
*Acinolaemus cf. mueangonensis* from Loei Province, Mueang Loei district (coll. HA). **A–D**. Shell; **E, F**. Enlarged views of the aperture; **G**. Enlarged view of the peristome surface; **H**. Enlarged view of the protoconch; **I**. Enlarged view of the last whorl surface sculpture; **J**. Enlarged view of the last whorl surface sculpture near the umbilicus.

#### Acinolaemus rhamphodontis

Taxon classification

Animalia

StylommatophoraHypselostomatidae

Tongkerd & Panha, 2025

68E100D0-DC4A-5B11-A90C-A4156EB8846B

Figs 7L, 27, 30

Acinolaemus rhamphodontis Tongkerd & Panha in Tongkerd et al., 2025: 63–66, figs 16, 17, table 4.

##### Type locality.

“Phra Phutthabat Doi Khao Nam, Ban Na subdistrict, Sam Ngao District, Tak Province; 17°14'56.4"N, 98°56'16.3"E” (Thailand).

**Figure 27.**
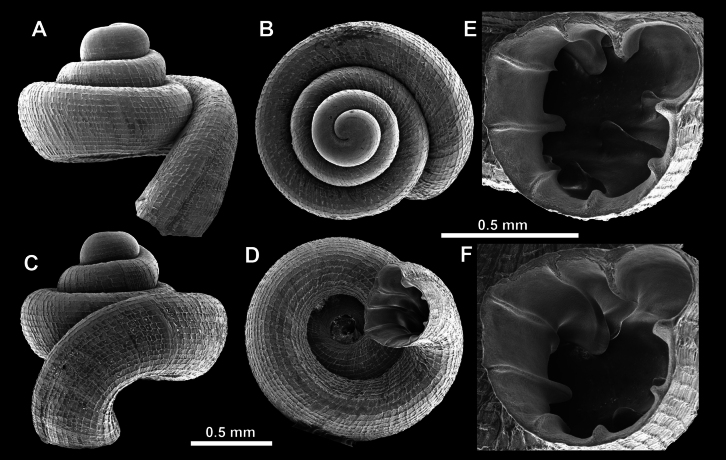
*Acinolaemus rhamphodontis*, holotype (CUMZ 14449) (from Tongkerd et al. 2025). **A–D**. Shell; **E, F**. Enlarged views of the aperture.

##### Material examined.

**Thailand** • holotype; CUMZ 14449.

##### Differential diagnosis.

This species differs from all other congeners by the strongly detached last whorl, which is also descending near the aperture. From *Clostophis* species with similar shell morphology (*C. sankeyi* W. H. Benson, 1860, *C. yoga* Páll-Gergely & Hunyadi, 2022, *C. udayaditinus* Sutcharit & Panha, 2025, *C. rhynchotes* Tongkerd & Panha, 2025), this species differs by the presence of numerous, elongated apertural barriers reaching the peristome edge in small pliculae.

##### Distribution.

This species is only known from the type locality (Tongkerd et al. 2025).

#### Acinolaemus zed

Taxon classification

Animalia

StylommatophoraHypselostomatidae

Gojšina, Hunyadi & Páll-Gergely sp. nov.

3D7FC593-97DE-5471-B2FA-74C7D7583D97

https://zoobank.org/2F9593A9-DE57-487A-97F1-FEEA94F5802E

Figs 7M, 28, 30

##### Type material.

***Holotype*. Myanmar** • 1 shell; Mon State, ca 14 km northeast from the center of Mawlamyine, Kalagon, Kha Yone cave; 16°31.9856'N, 97°42.9102'E; 11 Oct 2018; A. Hunyadi, K. Okubo, J. U. Otani leg.; CUMZ 15456. ***Paratypes*. Myanmar** • 37 shells; same data as for holotype; coll. HA.

**Figure 28.**
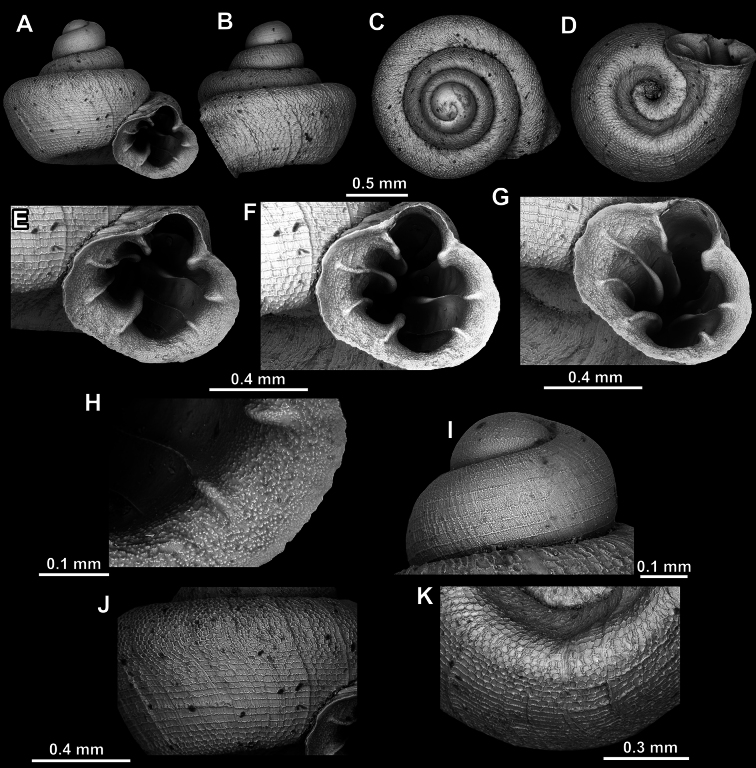
*Acinolaemus zed* sp. nov., holotype (CUMZ 15456). **A–D**. Shell; **E–G**. Enlarged views of the aperture; **H**. Enlarged view of the peristome surface; **I**. Enlarged view of the protoconch; **J**. Enlarged view of the last whorl surface sculpture; **K**. Enlarged view of the last whorl surface sculpture near the umbilicus.

##### Additional material examined.

**Myanmar** • 3 shells (damaged/juveniles = not paratypes); same data as for holotype; coll. HA.

##### Diagnosis.

Shell colorless, depressed concave-conical. Last whorl shouldered, surface sculpture honeycomb-like. Apertural barriers numerous, slender. Umbilicus wide, with both a strong periumbilical keel and a deep groove.

##### Description.

Shell depressed, concave-conical, light yellowish, consisting of 3.75–4 whorls separated by deep suture; protoconch with ~ 10 spiral striae (clearer terminally), consisting of ~ 1.25 whorls; boundary between protoconch and teleoconch distinct due to change of surface sculpture (rougher, with both radial and spiral growth elements); teleoconch surface sculpture honeycomb-like; lower portion of the body whorl sometimes with more distinct spiral striae while upper portion has sculpture of honeycomb-like pattern; in some specimens, last whorl is entirely sculptured with honeycomb-like pattern; last whorl shouldered and slightly descending near aperture; peristome finely granulated, expanded (especially at palatal and columellar sides, not around sinulus), not reflected; no distinct cervical swelling or constriction present; aperture equipped with numerous slender apertural barriers; parietal lamella high, curved, blunt, almost fully reaching peristome edge; angular lamella of similar appearance, reaching peristome edge, consisting of inner (higher and broader) and outer (lower and narrower) blunt-topped portions separated by short, less elevated, blade-like interval; outer portion of angular lamella sigmoid; three main palatal plicae present; upper palatal plica higher and thicker at inner portion, while lower and more slender (blade-like) towards peristome; low and slender suprapalatal plica present above upper palatal; interpalatal plica widest among all palatals but becomes significantly narrower and blade-like towards peristome, reaching it in form of blunt plicula; palatal tubercle strong, sitting in front of upper palatal plica and appearing slightly below it in standard view; lower palatal plica of same morphology as upper palatal, slightly thinner and lower, reaching the peristome in form of blunt plicula; infrapalatal plica slightly lower than lower palatal; inner portion of infrapalatal plica blunt, sloping as blade-like, not reaching peristome; columellar lamella wide, oblique, blunt and almost fully reaching peristome edge; infraparietal lamella lower than both parietal and columellar, situated halfway between them, blunt; umbilicus wide, ~ ^1^/_3_ of shell width; strong periumbilical keel running along umbilicus; deep groove present inside the umbilicus.

##### Differential diagnosis.

This species differs from *A. microcubus* sp. nov. by the stronger periumbilical keel and the presence of a deep groove inside the umbilicus as well as the much less strongly shouldered last whorl. *Acinolaemus microcubus* sp. nov. is also a bit smaller than *A. zed* sp. nov. *Acinolaemus dayanus* has a narrower umbilicus, lacks both the periumbilical keel and the umbilical groove, has a less distinctly shouldered last whorl and has overall weaker (lower) apertural barriers. *Acinolaemus humilis* sp. nov. is similar in apertural barrier arrangement and strength but the shell is smaller, has a less shouldered last whorl, and lacks the periumbilical keel as well as the umbilical groove. Also, peristomal pliculae are lacking in *A. humilis* sp. nov.

##### Measurements (in mm, n = 5).

SH = 1.21–1.34; SW = 1.56–1.68, AH = 0.57–0.67; AW = 0.65–0.71.

##### Distribution.

This species is known only from the type locality.

##### Etymology.

The specific epithet zed is derived from the Latin *zeta* referring to the fact that this is the last species to be described herein in this species group of *Acinolaemus*.

##### Remarks.

The difference between the sculpture of the upper portion of the last whorl (typically honeycomb-like) and the lower portion (with distinct spiral striae) was observed in 14 paratypes. The pronounced periumbilical keel and a deep umbilical groove seem to be characteristic for this species as these characters were consistent in all paratypes.

#### Acinolaemus

Taxon classification

Animalia

StylommatophoraHypselostomatidae

sp. 1

AFD997A9-2C31-5092-8450-E79AF73095B7

Figs 7N, 29, 30

##### Material examined.

**Thailand** • 1 whole shell and 3 fragments; Tak Province, ravine 16 km NNW Mae Ramat; 17°3.5'N, 98°26.6833'E; 220 m a.s.l.; 03 May 1988; F. G. Thompson leg.; locality code FGT-4405; UF 347020.

**Figure 29.**
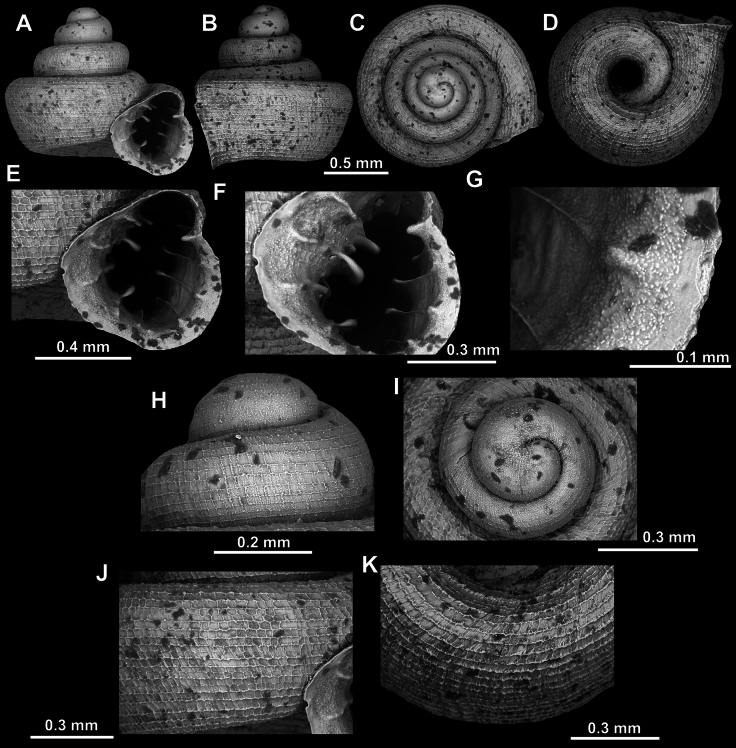
*Acinolaemus* sp. 1 from Tak Province, Thailand (UF 347020). **A–D**. Shell; **E, F**. Enlarged views of the aperture; **G**. Enlarged view of the peristome surface; **H, I**. Enlarged view of the protoconch; **J**. Enlarged view of the last whorl surface sculpture; **K**. Enlarged view of the last whorl surface sculpture near the umbilicus.

**Figure 30.**
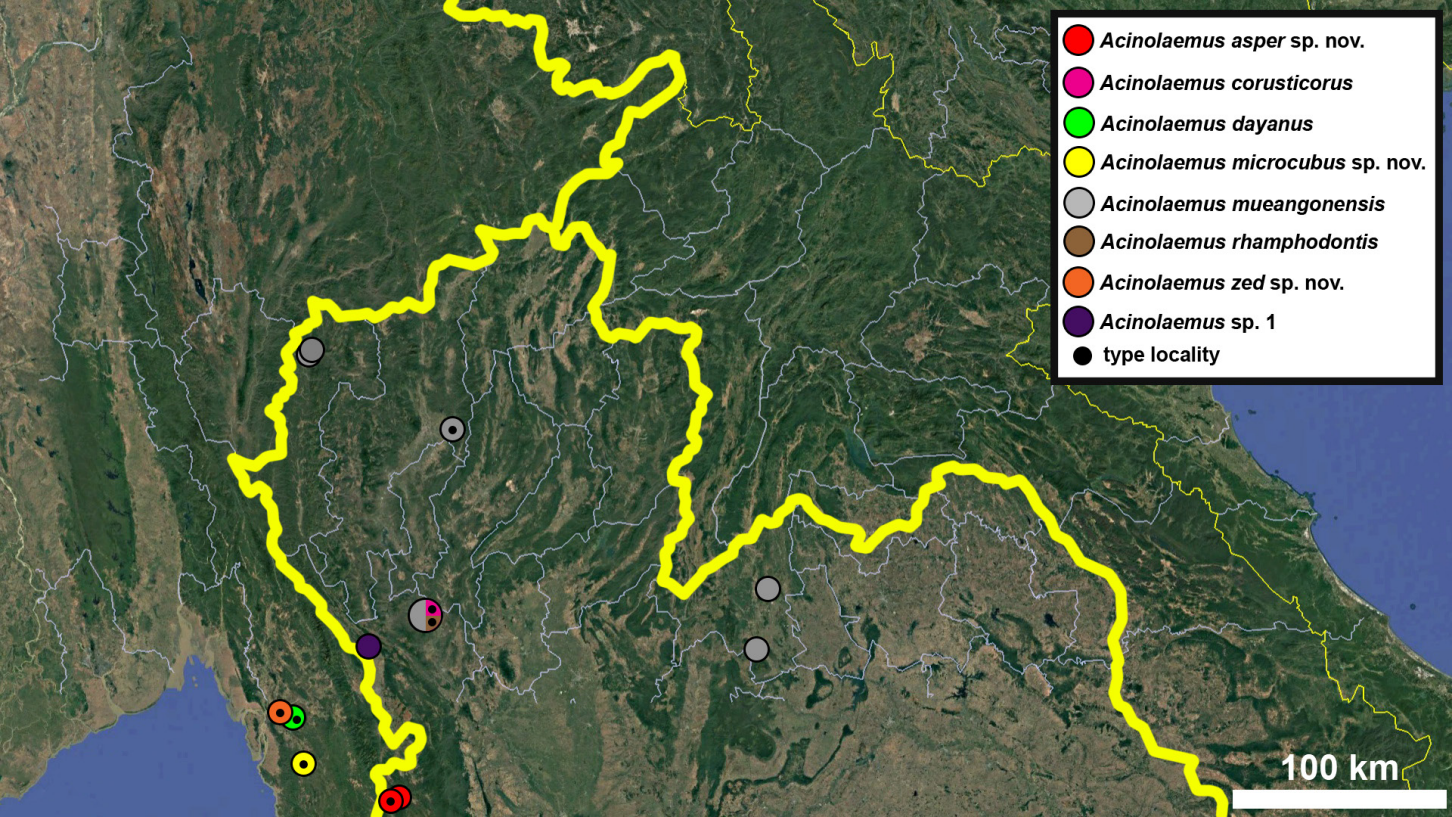
Distribution map of *Acinolaemus* species group with shell sculpture of honeycomb-like pattern and peristomal pliculae.

##### Remarks.

This is most likely an undescribed species. The single complete specimen is by the apertural barrier arrangement and shell shape most similar to *A. corusticorus*. However, the plicae in the undescribed species are much thinner (slender, blade-like) and there are also barriers inside the sinulus (absent in *A. corusticorus*). However, as we have just a single complete specimen, we choose not to describe it here due to the striking similarity with *A. corusticorus*.

###### III. *Acinolaemus ferox* group – species with strong spiral striae, weak radial lines, and peristomal pliculae

**Remarks**. This species group is characterized by distinctly stronger and more numerous spiral striae than radial lines as well as apertural barriers reaching the peristome edge as small pliculae. Two species are assigned to this group: *A. ferox* sp. nov. and *A. profundus* sp. nov.

#### Acinolaemus ferox

Taxon classification

Animalia

StylommatophoraHypselostomatidae

Gojšina, Tongkerd & Páll-Gergely sp. nov.

EB6C4B37-8F56-5C90-A00E-50F6CD185AF1

https://zoobank.org/501F3B6E-7324-4655-B5B3-29CC80699DCB

Figs 7O, 31, 33

##### Type material.

***Holotype*. Thailand** • 1 shell; Chiang Rai Province, limestone knoll, 4 km NE of Ban Pa Ngae limestone knoll, base of limestone ledge; 19°34.3167'N, 99°59.2333'E; 410 m a.s.l.; 12 May 1988; F. G. Thompson leg.; locality code FGT-4429; UF 591354.

**Figure 31.**
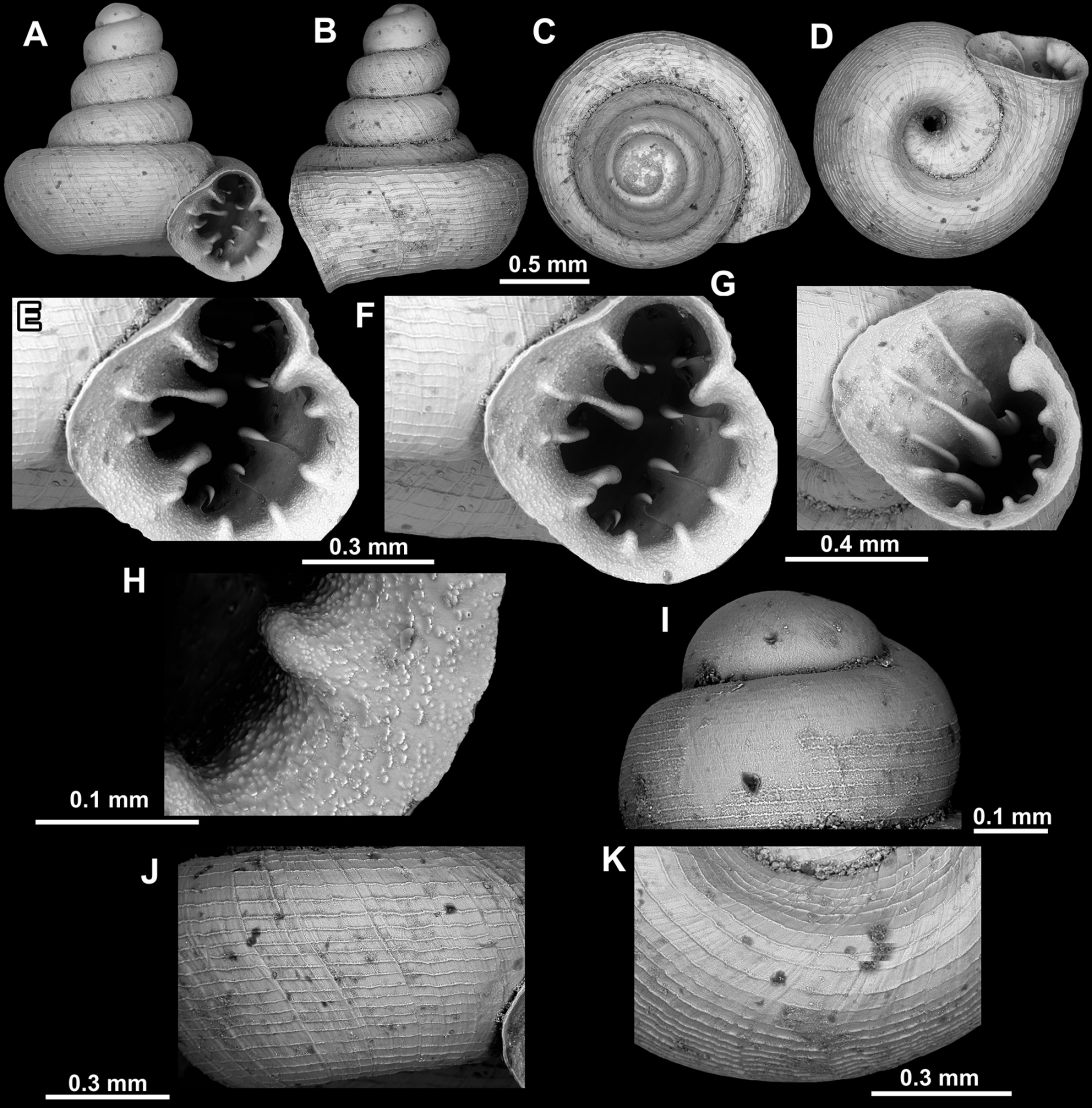
*Acinolaemus ferox* sp. nov., holotype (UF 591354). **A–D**. Shell; **E–G**. Enlarged views of the aperture; **H**. Enlarged view of the peristome surface; **I**. Enlarged view of the protoconch; **J**. Enlarged view of the last whorl surface sculpture; **K**. Enlarged view of the last whorl surface sculpture near the umbilicus.

##### Diagnosis.

Shell concave-conical, colorless. Last whorl weakly shouldered, teleoconch strongly spirally straited. All palatal plicae and angular lamella with hooked inner portions and slender outer portions which reach the peristome edge as small pliculae. Umbilicus wide, ~ ^1^/_2_ of the shell width with a blunt periumbilical keel.

##### Description.

Shell concave-conical, probably light yellowish (but not well visible due to slightly corroded specimen), consisting of almost 5 convex whorls separated by deep suture; protoconch consisting of ~ 1.5 whorls and ~ 15 dense, equidistant spiral striae, clearer terminally; boundary between protoconch and teleoconch distinct due to change of surface sculpture (becoming more prominent on teleoconch and with stronger radial growth lines); last whorl weakly shouldered, adnate to penultimate and slightly descending near aperture; surface sculpture of shell consisting of raised spiral striae crossed by weaker and much sparser radial growth lines; peristome finely granulated, expanded (except around sinulus), but not reflected; no distinct cervical swelling or constriction present; aperture equipped with nine barriers; parietal lamella strongly oblique, high and very thick, directed towards palatal wall, almost fully reaching peristome where it becomes quite low; angular lamella bipartite, consisting of inner, hooked part directed outside aperture and outer, slender portion which reaches peristome edge; two portions are blunt-topped, separated by blade-like, less elevated, short interval; all palatal plicae and basal plica with inner, hooked portions pointing outside, and much lower, slender and blade-like outer portions that reach expanding peristome in form of blunt pliculae; upper palatal plica slightly thinner than interpalatal and lower palatal; interpalatal and lower palatal plicae equally strong; infrapalatal and basal plicae approximately as same as upper palatal; columellar lamella broad, blunt and approximately as high as parietal, oblique and almost reaching peristome; columellar lamella getting lower towards peristome; infraparietal lamella blunt, half as high as columellar and parietal, situated halfway between them; sinulus small, distinctly separated from remainder of aperture; umbilicus wide, ~ ^1^/_3_ of shell width; blunt periumbilical keel present behind peristome and terminating at ~ ^1^/_2_ of last whorl.

##### Differential diagnosis.

See under *A. ptychochilus*.

##### Measurements (n = 1, in mm).

SH = 1.5; SW = 1.5; AH = 0.62; AW = 0.65.

##### Distribution.

This species is known only from the type locality.

##### Etymology.

The specific epithet is the Latin ferox meaning fierce or harsh, referring to numerous hooked apertural barriers.

#### Acinolaemus profundus

Taxon classification

Animalia

StylommatophoraHypselostomatidae

Gojšina, Auffenberg & Páll-Gergely sp. nov.

6608C685-3FD9-5A4A-8BCD-D63F93201B16

https://zoobank.org/00BF65D4-BBC8-4C64-87A5-38382E36FF27

Figs 7P, 32, 33

##### Type material.

***Holotype*. Thailand** • 1 shell; Mae Hong Son Province, 34.0 km NW Pai, Ban Nam Rin village; 19°27'N, 98°20'E; 940 m a.s.l.; 18 Mar 1988; K. Auffenberg leg.; locality code KA-0573; UF 345958. ***Paratypes*. Thailand** • 130 shells; same data as for holotype; UF 593505 • 2 shells; same data as for holotype; CUMZ 15428 • 7 shells; same data as for holotype; locality code KA-0572; UF 345951 • 1 shell; Mae Hong Son Province, 35.1 km S Mae Hong Son, 1.7 km E route 108; 19°5'N, 97°58'E; 850 m a.s.l.; 23 Mar 1988; K. Auffenberg leg.; locality code KA-0597; UF 591367 • 6 shells; Mae Hong Son Province, Mae Hong Son, road 1095; 19°25'N, 97°58'E; 390 m a.s.l.; 22 Mar 1988; K. Auffenberg leg.; locality code KA-0595B; UF 593507 • 151 shells; Mae Hong Son Province, 42 km NW of Pai, road 1095; 19°32'N, 98°13'E; 750 m a.s.l.; 20 Mar 1988; K. Auffenberg leg.; locality code KA-0584; UF 345583 • 6 shells; Mae Hong Son Province, Tham Nam Lod 800 m – Buddhist temple; 19°33.928'N, 98°16.794'E; 650 m a.s.l.; 09 Feb 2015; A. Hunyadi leg.; coll. HA.

**Figure 32.**
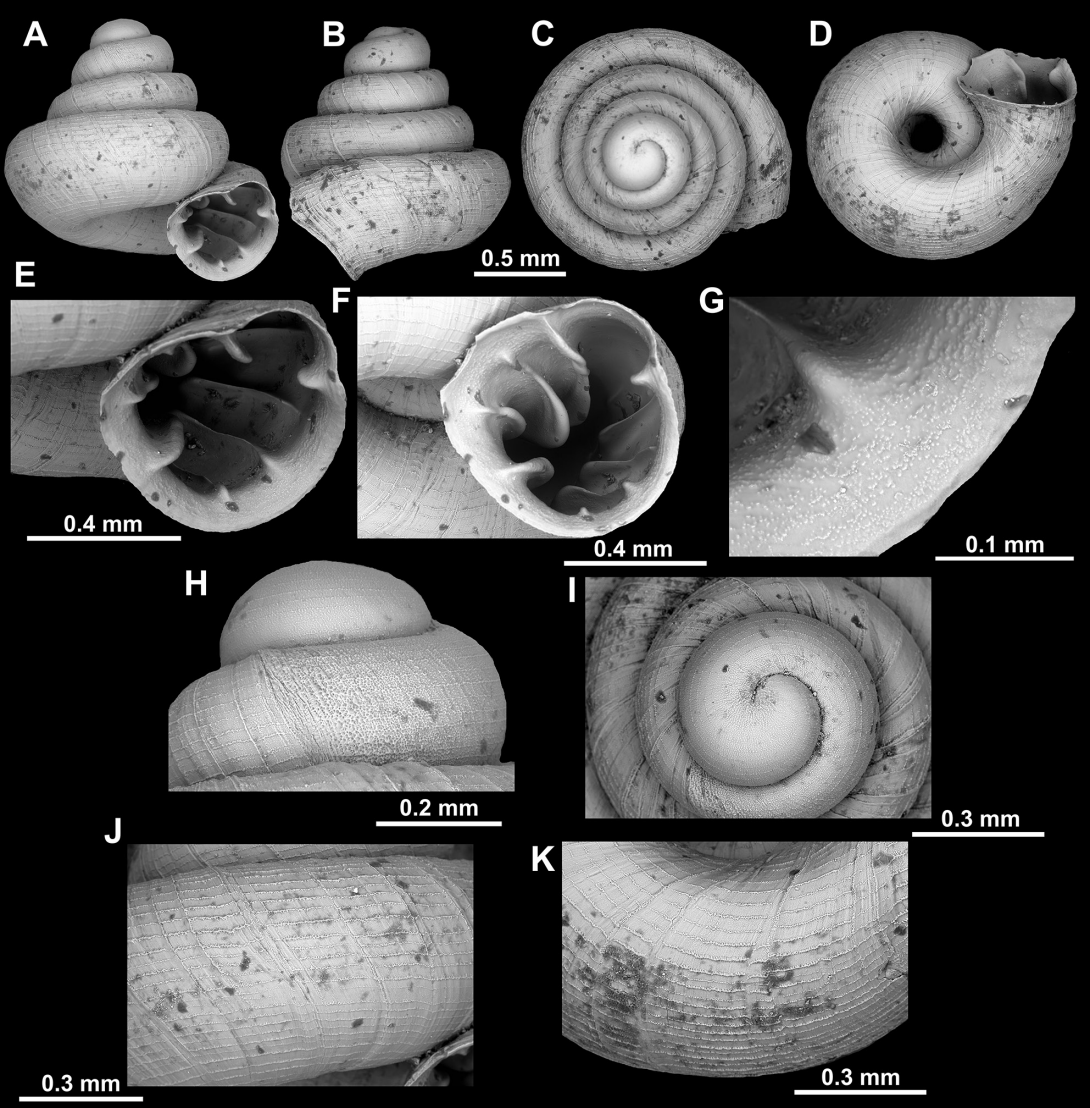
*Acinolaemus profundus* sp. nov., holotype (UF 345958). **A–D**. Shell; **E, F**. Enlarged views of the aperture; **G**. Enlarged view of the peristome surface; **H, I**. Enlarged view of the protoconch; **J**. Enlarged view of the last whorl surface sculpture; **K**. Enlarged view of the last whorl surface sculpture near the umbilicus.

**Figure 33.**
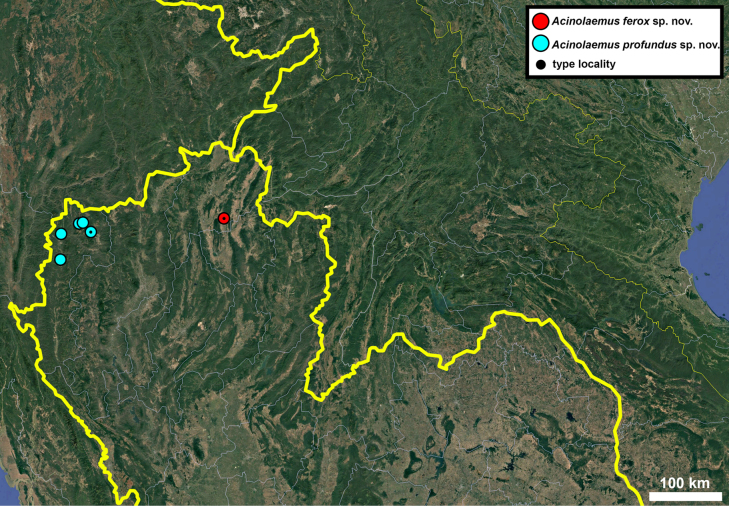
Distribution map of *Acinolaemus* species group with strong spiral striae, weak radial lines and peristomal pliculae.

##### Additional material examined.

**Thailand** • 10 shells (damaged/juveniles = not paratypes); same data as for holotype; UF 593506 • 8 shells (damaged/ juveniles = not paratypes); same data as for holotype; locality code KA-0572; UF 593508 • 1 shell (juvenile = not paratype); Mae Hong Son Province, Mae Hong Son, road 1095; 19°25'N, 97°58'E; 390 m a.s.l.; 22 Mar 1988; K. Auffenberg leg.; locality code KA-0595B; UF 593509 • 12 shells (damaged/ juveniles = not paratypes); Mae Hong Son Province, 42 km NW of Pai, road 1095; 19°32'N, 98°13'E; 750 m a.s.l.; 20 Mar 1988; K. Auffenberg leg.; locality code KA-0584; UF 591348 • 2 shells (damaged and juvenile = not paratypes); Mae Hong Son Province, Tham Nam Lod 800 m – Buddhist temple; 19°33.928'N, 98°16.794'E; 650 m a.s.l.; 09 Feb 2015; A. Hunyadi leg.; coll. HA.

##### Diagnosis.

Shell concave-conical, whorls very convex, suture very deep. Aperture equipped with nine or ten elongated barriers and a palatal tubercle. Umbilicus wide, ~ ^1^/_4_ of the shell width.

##### Description.

Shell concave-conical, colorless, consisting of 3.75–4 convex whorls separated by very deep suture; protoconch spirally striated, with ~ 13 terminally clearer spiral striae, consisting of ~ 1.25 whorls; boundary between protoconch and teleoconch distinct due to change in surface sculpture (much stronger radial and spiral growth elements on teleoconch); last whorl convex, rounded, descending near aperture; teleoconch sculpture consisting of raised spiral striae crossed by nearly equally strong, but irregular radial growth lines; peristome finely granulated, weakly expanded (except around sinulus), not reflected; no cervical swelling or constriction visible behind peristome; aperture equipped with nine or ten barriers (parietal, angular, suprapalatal (sometimes absent), upper palatal, interpalatal, lower palatal, basal, subcolumellar (sometimes absent), columellar, infraparietal); parietal lamella high, blunt, long, higher in middle portions and sloping towards peristome and almost reaching its edge; angular lamella of similar appearance as parietal, slightly lower, thinner, more curved, but longer, and reaching peristome; all palatal plicae and basal plica blunt and higher at their inner portions, sloping as less elevated, blade-like; upper palatal plica low and slender, thinnest among main palatals; suprapalatal plica very short, low and indistinct, occasionally absent; interpalatal plica slightly higher than upper palatal but slightly lower than lower palatal (both of similar morphology as upper palatal); distinct palatal tubercle (sometimes very weak, rarely absent) situated in front of upper palatal plica and as appears slightly below it in standard view; basal plica low and slender, situated approximately halfway between lower palatal and columellar; subcolumellar lamella, if present, low and shorts, columellar lamella oblique, nearly as broad as parietal; infraparietal lamella moderate, situated halfway between columellar and parietal lamellae; two weak peristomal pliculae present, corresponding to lower palatal plica and basal plica; sinulus small and distinctly separated from remainder of aperture; umbilicus wide, ~ ^1^/_3_–^1^/_4_ of shell width.

##### Differential diagnosis.

*Acinolaemus cryptidentatus* has a honeycomb-like surface, less elongated barriers, less rounded whorls, and a more slender shell. *Acinolaemus mueangonensis* has a honeycomb-like surface and slenderer shell.

##### Measurements (n = 5, in mm).

SH = 1.19–1.37; SW = 1.39–1.47; AH = 0.52–0.61; AW = 0.55–0.58.

##### Distribution.

This species is known from several localities in Mae Hong Son Province, Thailand.

##### Etymology.

The species name is from the Latin profundus, meaning deep, in reference to its deep suture.

###### IV. *Acinolaemus ptychochilus* group – species with reticulated surface pattern and peristomal pliculae

**Remarks**. This species group is characterized by the spiral lines crossed by equally developed radial growth lines, thus presenting a reticulated pattern as well as long, slender apertural barriers reaching the peristome edge as small pliculae. Two species are assigned to this group: *A. paucidentatus* sp. nov. and *A. ptychochilus*.

#### Acinolaemus paucidentatus

Taxon classification

Animalia

StylommatophoraHypselostomatidae

Gojšina, Tongkerd & Páll-Gergely sp. nov.

9C6ECD1C-0DD2-5DD5-A428-05F9A00327AE

https://zoobank.org/912679AC-F2C3-420F-8E30-173792F5DDE6

Figs 7Q, 34, 35, 39

##### Type material.

***Holotype*. Thailand** • 1 shell; Chiang Mai Province, 20 k SSW of Ban Mae Khi 700 m limestone leaf litter; 19°34.68'N, 99°4.86'E; 20 Jun 1987; F. G. Thompson leg.; locality code FGT-4334; UF 529566.

**Figure 34.**
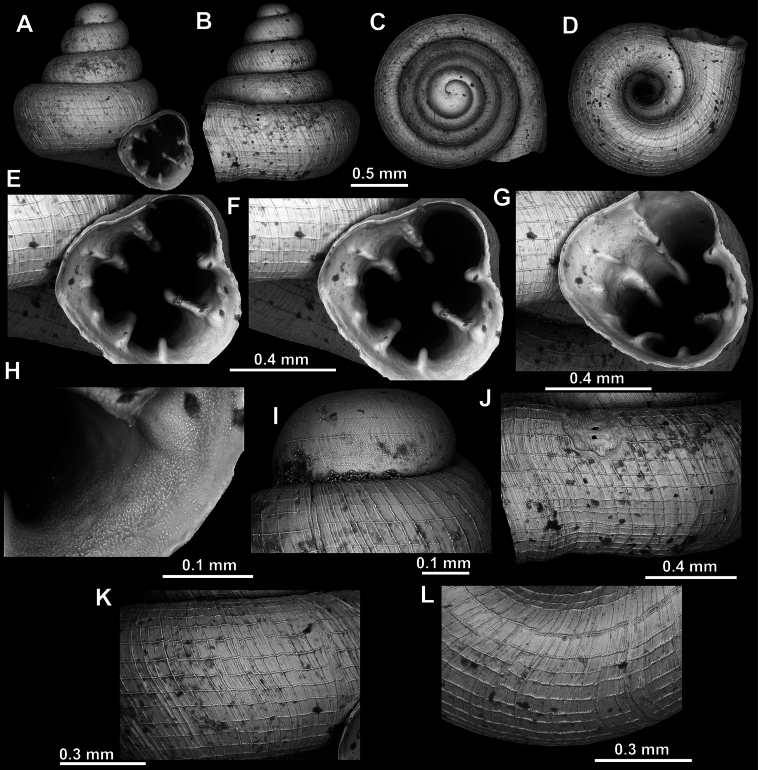
*Acinolaemus paucidentatus* sp. nov., holotype (UF 529566). **A–D**. Shell; **E–G**. Enlarged views of the aperture; **H**. Enlarged view of the peristome surface; **I**. Enlarged view of the protoconch; **J**. Enlarged view of the last whorl near the aperture; **K**. Enlarged view of the last whorl surface sculpture; **L**. Enlarged view of the last whorl surface sculpture near the umbilicus.

**Figure 35.**
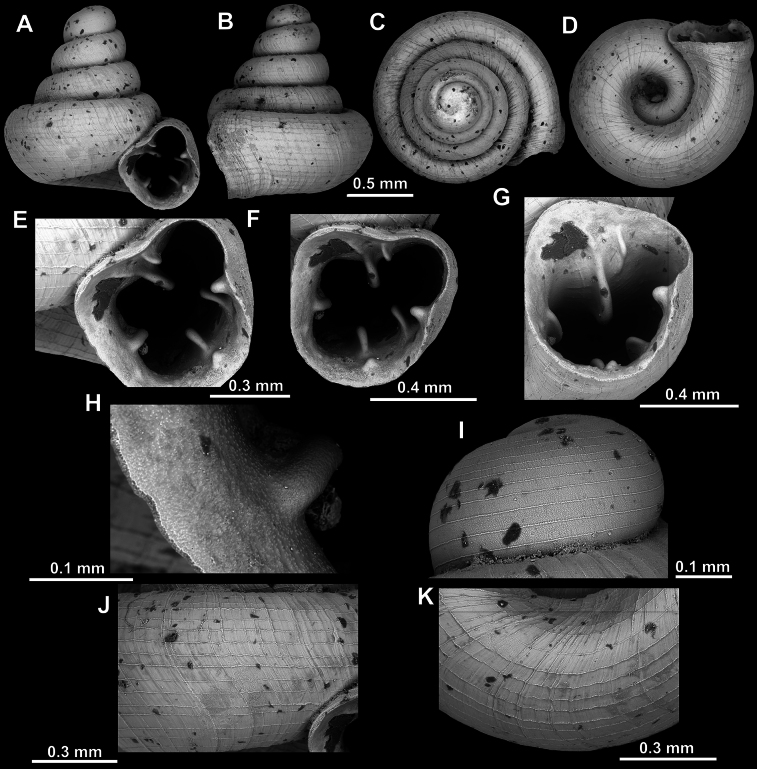
*Acinolaemus paucidentatus* sp. nov. from Chiang Rai Province, not paratype (coll. HA). **A–D**. Shell; **E–G**. Enlarged views of the aperture; **H**. Enlarged view of the peristome surface; **I**. Enlarged view of the protoconch; **J**. Enlarged view of the last whorl surface sculpture; **K**. Enlarged view of the last whorl surface sculpture near the umbilicus.

##### Additional material examined.

**Thailand** • 10 shells (not paratypes); Chiang Rai Province, 7+2 km south-southwest from Mae Sai, vicinity of Wat Tham Pla; 20°19.723'N, 99°51.817'E; 400 m a.s.l.; 12 Feb 2015; A. Hunyadi leg.; coll. HA. • 1 shell (damaged = not paratype); same data as for holotype; UF 593510.

##### Diagnosis.

A colorless *Acinolaemus* with six main blunt and short apertural barriers. Shell sculpture reticulated (i.e., raised spiral striae crossed by equally strong radial growth lines). Umbilicus wide, ~ ^1^/_3_ of the shell width.

##### Description.

Shell concave-conical, colorless, consisting of 4–4.25 convex, rounded whorls; protoconch very finely, coarsely spirally striated and consisting of ~ 1.5 whorls; boundary between protoconch and teleoconch distinct because sculpture becomes stronger on initial teleoconch whorls (including presence of radial growth lines); last whorl very weakly shouldered, almost parallel to shell axis near aperture; surface sculpture of teleoconch densely spirally striated; spiral striae crossed by equally strong, dense radial growth lines forming reticulated pattern; peristome finely granulated, expanded (except around sinulus), not reflected; cervical swelling and constriction behind peristome present, both vary from weak to conspicuous; aperture equipped with six barriers (parietal, angular, upper palatal, lower palatal, basal, and columellar); parietal lamella moderately high, not reaching peristome; angular lamella longer and more slender than parietal, reaching peristome edge, bipartite with inner (higher) and outer (lower) portions separated by less elevated but also blunt interval; two palatal plicae (upper and lower) relatively high, not very slender and of similar appearance (upper palatal being slightly lower than lower palatal); weak tubercle present in front of lower palatal plica, probably representing a reduced peristomal plicula; distinct palatal tubercle situated in front of upper palatal plica; basal plica low and short (more than two times lower than lower palatal), situated halfway between lower palatal plica and columellar lamella; columellar lamella broad, oblique, not reaching peristome; two tubercle-like swellings present above columellar lamella, one probably corresponding to supracolumellar lamella and other to infraparietal; sinulus small, distinctly separated from remainder of aperture; umbilicus wide, ~ ^1^/_3_ of shell width.

##### Differential diagnosis.

*Acinolaemus paucidentatus* sp. nov. has a reticulated shell surface sculpture (not honeycomb-like) and a wider umbilicus than *A. simplex* sp. nov.

##### Measurements (n = 1, in mm).

SH = 1.64; SW = 1.54; AH = 0.67; AW = 0.66.

##### Distribution.

This species is known from Chiang Mai and Chiang Rai Provinces in Thailand.

##### Etymology.

The specific epithet is a combination of the Latin *pauci* (few) and *dentatus* (teeth) referring to a relatively few apertural barriers when compared to the majority of congeners.

##### Remarks.

In specimens from Chiang Rai Province, we have noticed that they have slightly more reduced apertural barriers. All other characters (surface sculpture type, general barrier morphology and arrangement and umbilicus width) are shared with specimens from Chiang Mai Province (type locality) which is why we treat them conspecific. However, specimens from Chiang Rai Province are not designated as paratypes.

#### Acinolaemus ptychochilus

Taxon classification

Animalia

StylommatophoraHypselostomatidae

F. G. Thompson & Upatham, 1997

D226A057-C184-5734-85E9-781EC412F0D2

Figs 7R, 36–39

Acinolaemus ptychochilus F. G. Thompson & Upatham, 1997: 225–226, figs 7–11.
Acinolaemus ptychochilus — Changlom et al. 2019: 156; Panha and Burch 2008: 41–43, fig. 40; Schileyko 2011: 4; Dumrongrowjattana and Wongkamhaeng 2024: 321, 323, 330, fig. 7, 13, table 3; Tongkerd et al. 2025: 50–52, figs 8, 9, table 4.

##### Type locality.

“Thailand, Chiang Mae Province, Doi Pha San Sao (Mountain), 3 km west of Ban Prang Ma-O; 500 m altitude (19°26.0'N, 99°03.5'E)”.

**Figure 36.**
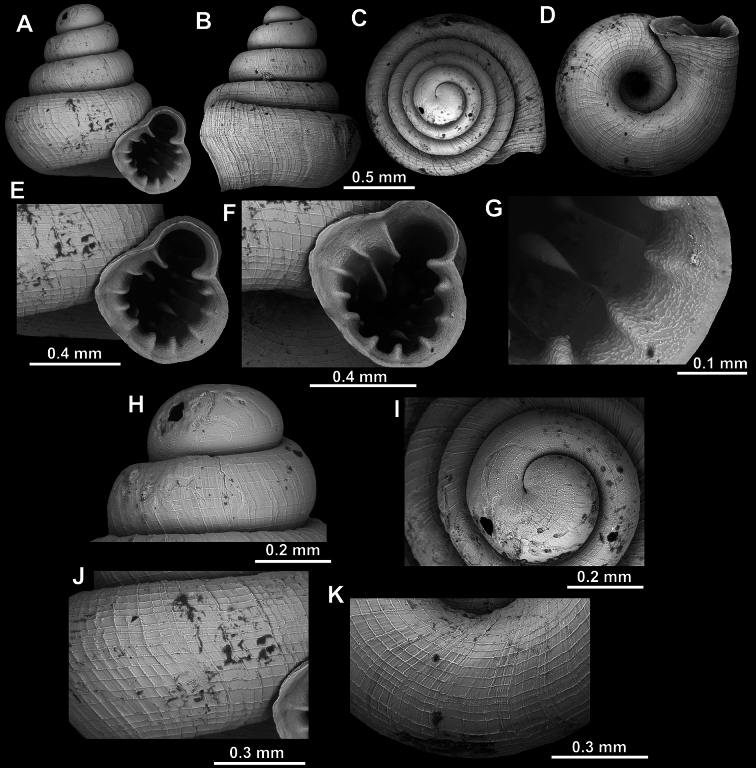
*Acinolaemus ptychochilus*, holotype (UF 113502). **A–D**. Shell; **E, F**. Enlarged views of the aperture; **G**. Enlarged view of the peristome surface; **H, I**. Enlarged views of the protoconch; **J**. Enlarged view of the last whorl surface sculpture; **L**. Enlarged view of the last whorl surface sculpture near the umbilicus.

**Figure 37.**
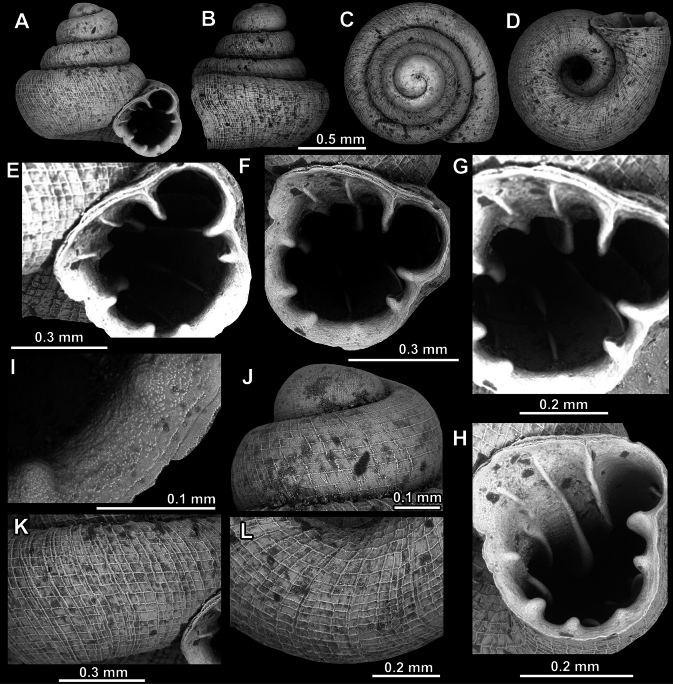
*Acinolaemus ptychochilus* from Nan Province (UF 347336). **A–D**. Shell; **E–H**. Enlarged views of the aperture; **I**. Enlarged view of the peristome surface; **J**. Enlarged view of the protoconch; **K**. Enlarged view of the last whorl surface sculpture; **L**. Enlarged view of the last whorl surface sculpture near the umbilicus.

**Figure 38.**
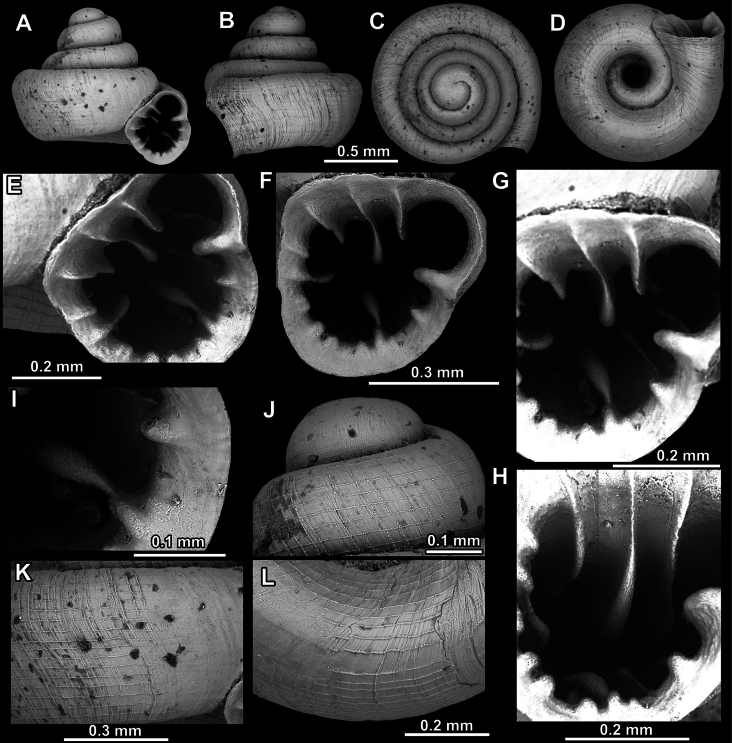
*Acinolaemus ptychochilus* from Chiang Rai Province (UF 593511). **A–D**. Shell; **E–H**. Enlarged views of the aperture; **I**. Enlarged view of the peristome surface; **J**. Enlarged view of the protoconch; **K**. Enlarged view of the last whorl surface sculpture; **L**. Enlarged view of the last whorl surface sculpture near the umbilicus.

**Figure 39.**
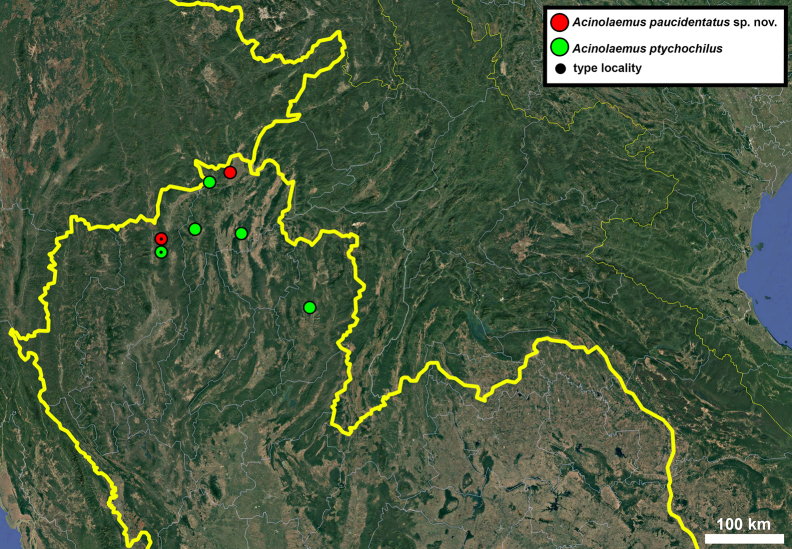
Distribution map of *Acinolaemus* species group with reticulated surface pattern and peristomal pliculae.

##### Type material examined.

**Thailand** • holotype; UF 113502 • 2 paratypes from the type locality; UF 113504 • 4 paratypes from the type locality; UF 113503.

##### Additional material examined.

**Thailand** • 4 shells; Nan Province, Ban Pha Tun, 11 km N of Nan; 18°50.95'N, 100°44.15'E; 260 m a.s.l.; 28 Apr 1988; F. G. Thompson leg.; sample code FGT-4392; UF 347336 • 5 shells; Chiang Rai Province, limestone knoll, Ban Mae Song Nai, 4 km N, 6 km NW of Mae Chan, limestone domes, shaded ledges; 20°11.6167'N, 99°33.1'E; 520 m a.s.l.; 10 May 1988; F. G. Thompson leg.; locality code FGT-4425; UF 593511 • 1 shell; Chiang Rai Province, limestone knoll, 4 km NE of Ban Pa Ngae, base of limestone ledge; 19°34.3167'N, 99°59.2333'E; 410 m a.s.l.; 12 May 1988; F. G. Thompson leg.; locality code FGT-4429; UF 593504 • 3 shells; Chiang Rai Province, limestone mountain, 6 km by road W of Ban Mae Suai; 19°38.9167'N, 99°26.6833'E; 650 m a.s.l.; 7 May 1988; F. G. Thompson leg.; locality code FGT-4415; UF 347100.

##### Differential diagnosis.

This species is similar to *A. ferox* sp. nov. which however has all palatal plicae hooked, as well as the inner portions of the angular lamella (*A. ptychochilus* has only the lower palatal hooked). The apertural barrier arrangement in *A. mueangonensis* is similar to *A. ptychochilus* but the latter has a different shell surface sculpture (distinctly spirally striated, not honeycomb-like). Also, no apertural barriers are hooked in *A. mueangonensis*. See also under *A. singularis* sp. nov.

##### Distribution.

This species is known from Chiang Mai, Chiang Rai and Nan provinces, Thailand.

##### Remarks.

One of the three paratypes of *A. ptychochilus* in UF 113504 represents *A. cryptidentatus*. Specimens from Nan Province (UF 347336) showed the same shell morphology as that of the type series, only with a slightly denser surface sculpture which we regard as intraspecific variability. Specimens from Chiang Rai Province (UF 593511) have non-hooked lower palatal plica but since this is the only difference observed, we treat them as *A. ptychochilus*.

###### V. *Acinolaemus singularis* group – species with reticulated surface pattern and no peristomal pliculae

**Remarks**. This species group is characterized by the spiral lines crossed by equally developed radial growth lines, thus presenting a reticulated pattern as well as the absence of peristomal pliculae. One species belongs to this group, *A. singularis* Gojšina, Tongkerd & Páll-Gergely, sp. nov.

#### Acinolaemus singularis

Taxon classification

Animalia

StylommatophoraHypselostomatidae

Gojšina, Tongkerd & Páll-Gergely sp. nov.

AF27F75C-325A-589F-8B9E-30D5D0EF5E81

https://zoobank.org/FDF04B66-5B14-414F-A274-BE3E8CF95856

Figs 7S, 40, 42

##### Type material.

***Holotype*. Thailand** • 1 shell; Chiang Rai Province, limestone knoll, Ban Mae Song Nai, 4 km N, 6 km NW Mae Chan, limestone domes, shaded ledges; 20°11.6167'N, 99°33.1'E; 520 m a.s.l.; 10 May 1988; F. G. Thompson leg.; locality code FGT-4425; UF 583728. ***Paratypes*. Thailand** • 3 shells; same data as for holotype; UF 591349.

**Figure 40.**
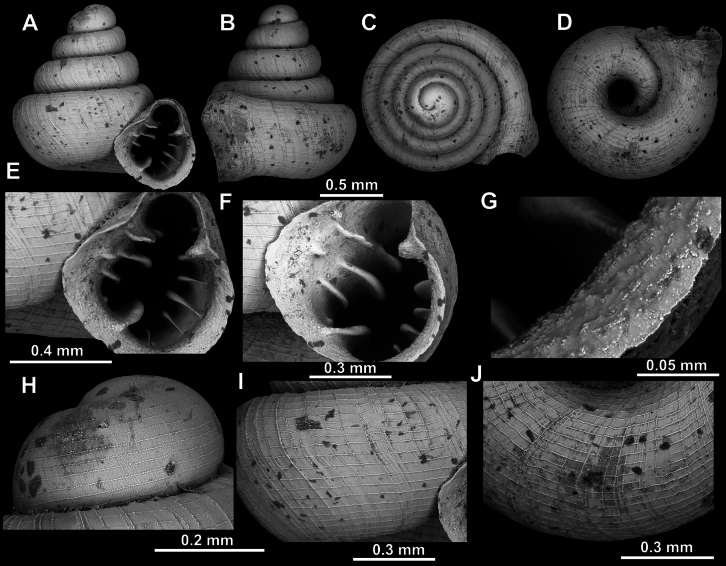
*Acinolaemus singularis* sp. nov., holotype (UF 583728). **A–D**. Shell; **E, F**. Enlarged views of the aperture; **G**. Enlarged view of the peristome surface; **H**. Enlarged view of the protoconch; **I**. Enlarged view of the last whorl surface sculpture; **J**. Enlarged view of the last whorl surface sculpture near the umbilicus.

##### Diagnosis.

*Acinolaemus* species with reticulated surface sculpture and no peristomal pliculae. There are 11 apertural barriers. Umbilicus wide, ~ ^1^/_3_ of the shell width.

##### Description.

Shell weakly concave-conical, light brownish, consisting of 4.5–5 convex whorls separated by deep suture; protoconch spirally striated and consisting of ~ 1.5 whorls with ~ 11 spiral striae; boundary between protoconch and teleoconch distinct because sculpture becomes stronger on initial teleoconch whorls (including presence of radial growth lines); last whorl shouldered, almost parallel to shell axis near aperture; surface sculpture of teleoconch spirally striated; spiral striae crossed by equally strong, radial growth lines forming reticulated pattern; peristome finely granulated, expanded, not reflected; moderate swelling present behind peristome, followed by distinct constriction; aperture equipped with 11 barriers (parietal, angular, upper palatal, two interpalatals, lower palatal, infrapalatal, basal, columellar, supracolumellar, and infraparietal); no peristomal pliculae present; parietal lamella high, not reaching peristome and sometimes with small additional portion anterior to it; angular lamella more slender than parietal, almost reaching peristome edge, bipartite with inner (higher) and outer (lower) portions separated by less elevated, short and blunt interval; upper palatal plica weaker than two interpalatal plicae below it; lower palatal plica widest and highest among all palatals; infrapalatal plica approximately same as interpalatals; palatal tubercle distinct; basal plica slightly weaker than infrapalatal, situated approximately halfway between lower palatal and columellar; columellar lamella strong, thick, slightly oblique, almost reaching peristome; supracolumellar lamella weak, tubercle-like; infraparietal lamella situated closer to parietal than to columellar, approximately as strong as interpalatal plicae; sinulus small, distinctly separated from remainder of aperture; umbilicus wide, ~ ^1^/_3_ of shell width, expanding at last whorl.

##### Differential diagnosis.

This is the only *Acinolaemus* species that has a combination of reticulated surface sculpture and no peristomal pliculae. Otherwise, it is most similar to *A. ptychochilus*. Apart from the absence of peristomal pliculae, the new species lacks the hooked apertural barriers which can separate it from *A. ptychochilus*.

##### Measurements (in mm, n = 4).

SH = 1.48–1.64; SW = 1.46–1.51; AH = 0.69–0.74; AW = 0.62–0.65.

##### Distribution.

This species is known only from the type locality.

##### Etymology.

The specific epithet is derived from the Latin singularis, meaning singular, the only one, which is a reference to the fact that this species is the only currently known representative of *Acinolaemus* with reticulated surface pattern and no peristomal pliculae.

###### VI. *Acinolaemus rugolabialis* group – species with unique characters

**Remarks**. Only *A. rugolabialis* sp. nov. belongs to this group. This species could not be assigned to any of the previous five groups due to the presence of very weak spiral striae on the last whorl, crossed by few and even weaker radial growth lines.

#### Acinolaemus rugolabialis

Taxon classification

Animalia

StylommatophoraHypselostomatidae

Gojšina, Hunyadi & Páll-Gergely sp. nov.

D02523F5-845C-5C54-A9F0-04882478ECC6

https://zoobank.org/7C6832FB-B225-4509-94E8-658778BDF1CB

Figs 7T, 41, 42

##### Type material.

***Holotype*. Myanmar** • 1 shell; Shan State, Hopong ca 22 km from the center of Hopong towards Namsang, Hkoche, Htem Sann Cave; 20°49.0836'N, 97°20.1192'E; 1240 m a.s.l.; 06 Oct 2018; A. Hunyadi, K. Okubo, J. U. Otani leg.; CUMZ 15457. ***Paratypes*. Myanmar** • 42 shells; same data as for holotype; coll. HA • 40 shells; Shan State, Kalaw, north-northeast, Osei Mountain Pagoda, northeast 150 m; 20°39.320'N, 96°34.927'E; 1565 m a.s.l.; 03 Oct 2018; A. Hunyadi leg.; coll. HA • 17 shells; Shan State, Kalaw, Shwe Oo Min Paya; 20°37.2616'N, 96°33.4735'E; 1340 m a.s.l.; 02 Oct 2018; A. Hunyadi leg.; coll. HA • 96 shells; Shan State, 13.5 km east-southeast from the center of Kalaw, Myinmati Taung; 20°35.4264'N, 96°36.7938'E; 1350 m a.s.l.; 03 Oct 2018; A. Hunyadi, K. Okubo & J. U. Otani leg.; coll. HA • 63 shells; Shan State, Taunggyi, west-southwest, Montawa Cave; 20°45.282'N, 97°01.0572'E; 1260 m a.s.l.; 05 Oct 2018; A. Hunyadi, K. Okubo & J. U. Otani leg.; coll. HA • 9 shells; Shan State, Taunggyi, hillside above Aye Say Tee, Dragon Cave; 20°47.4886'N, 97°03.0363'E; 1380 m a.s.l.; 08 Oct 2018; A. Hunyadi, K. Okubo & J. U. Otani leg.; coll. HA • 2 paratypes; Shan State, Hopong, Sam Phu, Cave Ae-5 at ridge above village Ho Hwe; 20°41.103'N, 97°16.198'E; 12 Feb 2019; J. Grego leg.; coll. JG.

**Figure 41.**
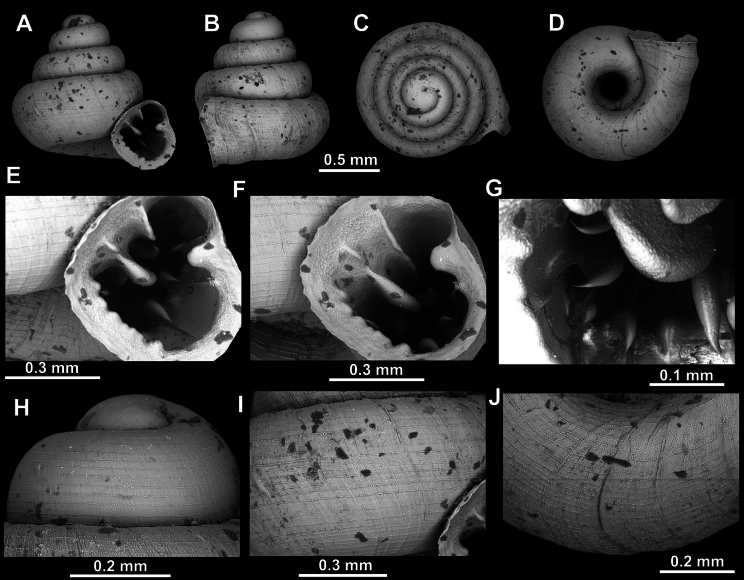
*Acinolaemus rugolabialis* sp. nov., holotype (CUMZ 15457). **A–D**. Shell; **E, F**. Enlarged views of the aperture; **G**. Enlarged view of the columellar lamella; **H**. Enlarged view of the protoconch; **I**. Enlarged view of the last whorl surface sculpture; **J**. Enlarged view of the last whorl surface sculpture near the umbilicus.

**Figure 42.**
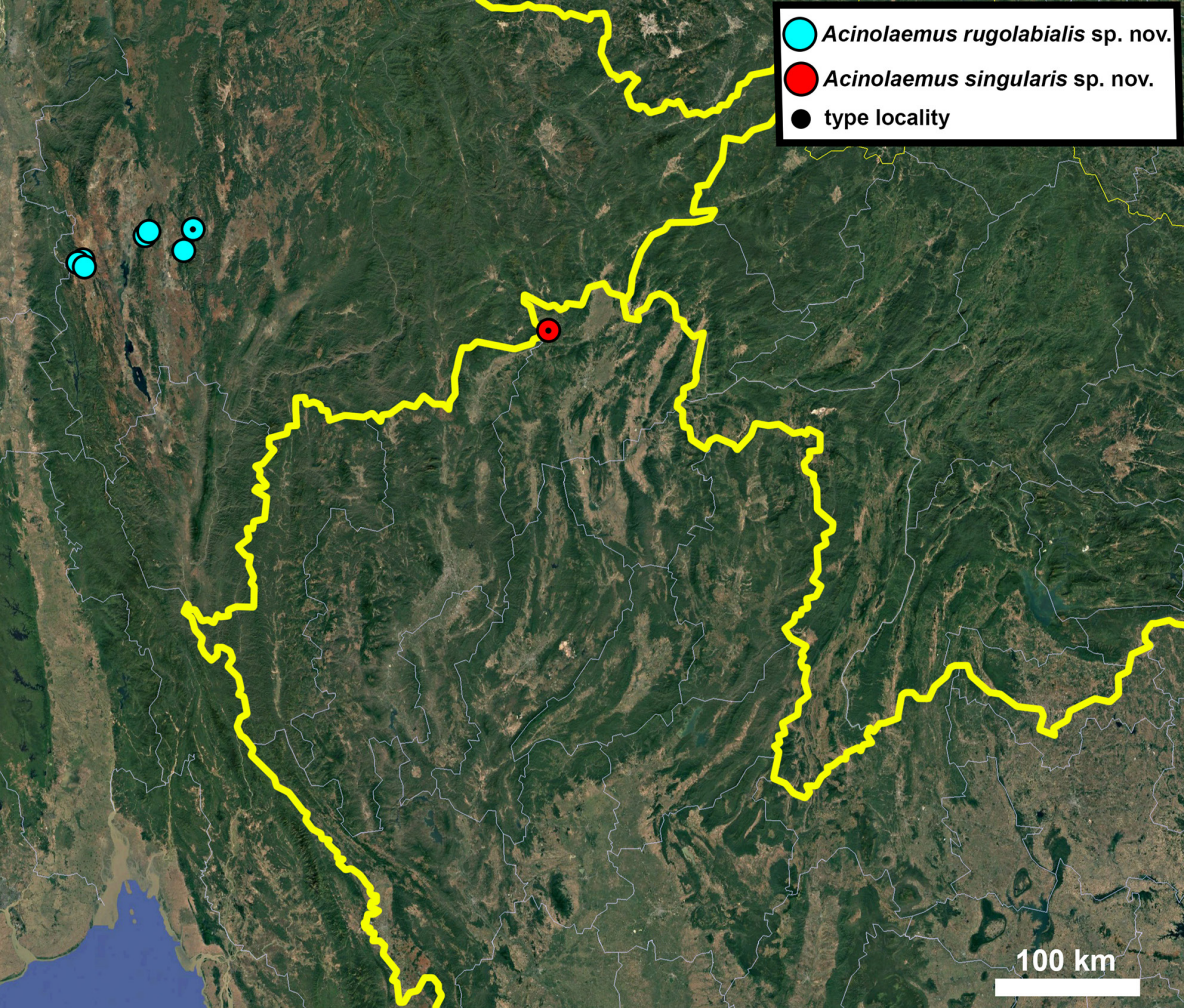
Distribution map of *A. singularis* sp. nov. and *A. rugolabialis* sp. nov.

##### Additional material examined.

**Myanmar** • 15 shells (juveniles = not paratypes); same data as for holotype; coll. HA • 13 shells (juvenile = not paratypes); Shan State, 13.5 km east-southeast from the center of Kalaw, Myinmati Taung; 20°35.4264'N, 96°36.7938'E; 1350 m a.s.l.; 03 Oct 2018; A. Hunyadi, K. Okubo, J. U. Otani leg.; coll. HA.

##### Diagnosis.

Shell conical, weakly spirally striated. Apertural barriers numerous, all main barriers except the parietal are hooked. Peristome wrinkled on the columellar side. Umbilicus wide, ~ ^1^/_3_ of the shell width.

##### Description.

Shell conical, colorless, consisting of 3.75–4 regularly increasing whorls separated by deep suture; protoconch consisting of ~ 1.25 whorls, initially almost smooth, terminally finely spirally striated (with ~ 12 spiral striae); boundary between protoconch and teleoconch distinct due to rougher surface sculpture of teleoconch; last whorl rounded, adnate to penultimate and almost parallel to shell axis near aperture; surface sculpture of shell consisting of fine spiral striae crossed by even finer, irregularly spaced radial growth lines; surface sculpture most prominent on last whorl, particularly around umbilicus; peristome strong, thick, finely granulated, expanded (except around sinulus) but not reflected; weak cervical swelling present behind peristome (slightly stronger than one found in e.g., *A. dayanus*, see Tongkerd et al. 2024: fig. 2A), followed by slight constriction immediately behind it; aperture equipped with eight main barriers (parietal, angular, upper palatal, interpalatal, lower palatal, basal, columellar, and infraparietal) and occasionally additional swelling-like barriers; parietal lamella highest and broadest in aperture, heart shaped in lateral view due to medial, blade-like interval separating blunter, higher and thicker inner and outer portions; outer portion of parietal lamella almost reaches peristome edge as very low, slender projection; angular lamella bipartite: inner portion high, in form of sharp hook pointing outside aperture and outer portion low, not hooked and reaching, or almost reaching peristome edge; two portions of angular lamella connected by less elevated, blade-like interval; palatal plicae (upper, inter, and lower) basal and those on columellar side are all of same morphology: with high inner, hooked portions and low, slender, long, and blade-like portions which do not reach peristome edge; upper palatal plica smallest among palatals, and lower palatal largest; two rounded swellings present on palatal wall: one between upper palatal and interpalatal plica and one between interpalatal and lower palatal plica; these swellings do not develop into proper barriers; palatal tubercle distinct, situated in front of upper palatal plica appears slightly below it in standard view; basal plica small, hooked, situated closer to lower palatal than to columellar; there may be small, blunt swelling between basal plica and lower palatal plica which most probably represents infrapalatal plica; subcolumellar lamella may be present with same form as infrapalatal plica; columellar lamella hooked and not very broad; peristome always distinctly wrinkled in front of columellar lamella; infraparietal lamella low, hooked, and positioned between columellar and parietal lamellae (but closer to parietal); sinulus small, well-separated from remainder of aperture and usually with two or three low plicae inside; umbilicus wide, ~ ^1^/_3_ of shell width.

##### Differential diagnosis.

This species is unique with the combination of a low, depressed shell, presence of numerous apertural barriers, all of which except the parietal are hooked (including the inner portion of the angular lamella) and a wide umbilicus. It is also readily recognized by the wrinkled peristome on the columellar side.

##### Measurements (in mm, n = 5).

SH = 1.19–1.31; SW = 1.28–1.41; AH = 0.49–0.57; AW = 0.5–0.58.

##### Distribution.

This species is known from seven localities in Shan State, Myanmar.

##### Etymology.

The specific epithet is derived from the Latin *rugosa* (wrinkled) and *labia* (lip), referring to the wrinkled columellar side of the peristome.

##### Remarks.

Shells may appear more or less depressed. Specimens from Dragon Cave showed a much stronger lower palatal plica.

#### Genus Clostophis

Taxon classification

Animalia

StylommatophoraHypselostomatidae

W. H. Benson, 1860

F7EBC9B2-BDA9-56B4-8BB2-8321304992DB

Clostophis Benson, 1860: 95.
Montapiculus Panha & Burch, 1999: 148.

##### Type species.

*Clostophis sankeyi* Benson, 1860, by monotypy.

##### Remarks.

The following species are transferred from *Acinolaemus* to *Clostophis*:

#### Clostophis carcharodon

Taxon classification

Animalia

StylommatophoraHypselostomatidae

(Vermeulen, Phung & Truong, 2007) comb. nov.

2EFF43A1-8C0B-5AE4-A56C-E0F3126DC122

Acinolaemus carcharodon Vermeulen, Phung & Truong, 2007: 87, fig. 4.
Acinolaemus carcharodon — Changlom et al. 2019: 156; Schileyko 2011: 4; Sutcharit et al. 2023: 1289–1290, fig. 1, table 1; Tongkerd et al. 2025: 53, table 4.

##### Type locality.

“Vietnam, Kien Giang Prov., Kien Luong, Hon Chong Hill”.

##### Differential diagnosis.

When compared to *C. carcharodon*, *C. udayaditinus* Sutcharit & Panha, 2025 has a less depressed shell, more strongly detached last whorl from the penultimate, and has a columellar lamella consisting of two hooked portions directed towards each other. See under *C. pyramidalis*.

##### Distribution.

This species is known from the Kien Giang Province, Vietnam and the Kampot Province, Cambodia.

##### Remarks.

This species is transferred to *Clostophis* due to the similar apertural barrier arrangement and morphology as in *C. lacrima* (Páll-Gergely & Hunyadi, 2015), *C. obliquus* Páll-Gergely & Hunyadi, 2022 and *C. socialis* (Páll-Gergely & Hunyadi, 2015) (i.e., strong and moderately long barriers, including two strong palatal plicae). Furthermore, *C. carcharodon* lacks the spiral sculpture on the protoconch, the peculiar honeycomb-like surface sculpture of the shell (present in many *Acinolaemus* species) and peristomal pliculae (present in *A. ptychochilus*, the type species of *Acinolaemus*).

#### Clostophis colpodon

Taxon classification

Animalia

StylommatophoraHypselostomatidae

(F. G. Thompson & Upatham, 1997) comb. nov.

D27D8405-0030-5DB7-A73F-210ED4B7B8EF

Fig. 43

Acinolaemus colpodon F. G. Thompson & Upatham, 1997: 229–230, figs 3, 26–31.
Acinolaemus colphodon [sic] — Changlom et al. 2019: 156.
Acinolaemus colpodon — Boonngam et al. 2008: 258, table 1; Panha and Burch 2008: 40–41, fig. 29; Dumrongrowjattana and Wongkamhaeng 2024: 321, 323, 330, fig. 7, 13, table 3; Tongkerd et al. 2025: 53, table 4.

##### Type locality.

“Thailand, Rayong Province, Khao Bot, ca 6 km north and 2 km west of Ban Syaek Batan; 150 m altitude (13°02.8'N, 101°38.5'E)”.

**Figure 43.**
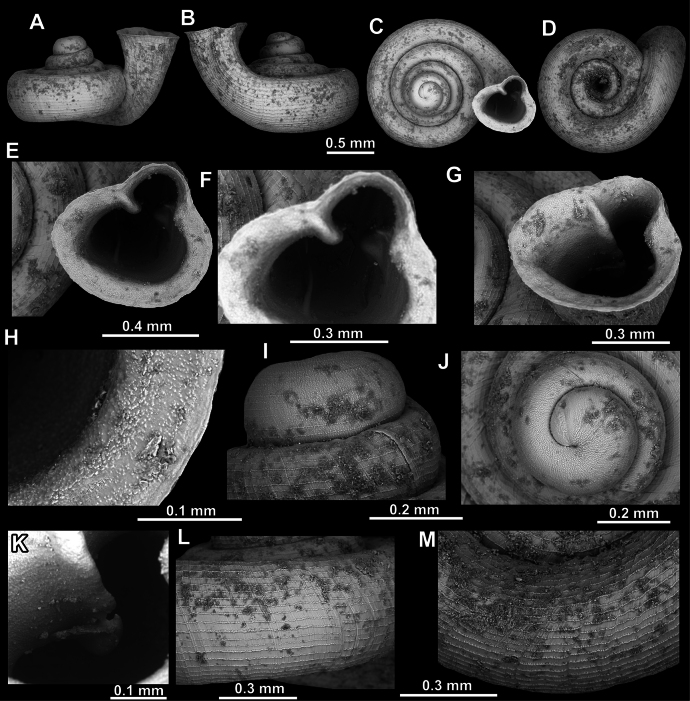
*Clostophis colpodon*, holotype (UF 113528). **A–D**. Shell; **E–G**. Enlarged views of the aperture; **H**. Enlarged view of the peristome surface; **I, J**. Enlarged views of the protoconch; **K**. Enlarged view of the angulo-parietal lamella; **L**. Enlarged view of the last whorl surface sculpture; **M**. Enlarged view of the last whorl surface sculpture near the umbilicus.

##### Type material examined.

**Thailand** • holotype; UF 113528 • 5 paratypes (UF 113529).

##### Additional material examined.

**Thailand** • 386 shells; Rayong Province, Khao Chamao district, Wat Tham Khao Pratun, gorge above the temple; 13°04.439'N, 101°35.850'E; 115 m a.s.l.; 09 Mar 2023; A. Hunyadi leg.; coll. HA. **Cambodia** • Banteay Meanchey Province, Sisophon N, Pongro W 1500 m, Prasat Phnom Korngva; 13.63241°N, 102.94427°E; 50 m a.s.l.; 28 Oct 2023; A. Hunyadi leg.; coll. HA.

##### Differential diagnosis.

This species is readily distinguished from other *Clostophis* and *Acinolaemus* species by the strikingly upwardly directed aperture (making it virtually parallel to the shell axis). This shape makes it superficially more similar to *Hypselostoma erawan* Panha & J. B. Burch, 2003 and *H. populare* Gojšina, Hunyadi & Páll-Gergely, 2025. *Hypselostoma erawan* has more apertural barriers (especially on the palatal side), and its angular and columellar lamellae are not hooked. *Hypselostoma populare* has none of its barriers hooked. Furthermore, both *H. erawan* and *H. populare* are much larger than *C. colpodon*, both are brownish (*C. colpodon* is colorless) and both have more elevated shells.

##### Distribution.

This species is known from Rayong and Chanthaburi Provinces, Thailand, as well as from Banteay Meanchey Province, Cambodia.

##### Remarks.

This species is transferred to *Clostophis* because it has only a single lamella on the parietal side (two in all *Acinolaemus* species) and it lacks the typical reticulated surface sculpture (as found in the type species of *Acinolaemus*) or characteristic honeycomb-like surface (as found in a number of *Acinolaemus* species). Furthermore, the apertural barrier arrangement is of the same type as that of *C. udayaditinus*, i.e., there is a strong palatal plica, hooked angular lamella and bipartite columellar lamella consisting of hooked inner and outer parts. See Dumrongrojwattana and Wongkamhaeng (2024) for additional sampling sites in Chanthaburi and Rayong provinces.

#### Clostophis pyramidalis

Taxon classification

Animalia

StylommatophoraHypselostomatidae

(Vermeulen, Phung & Truong, 2007) comb. nov.

B5608DC2-829E-5843-AB31-07713F3584B7

Montapiculus pyramidalis Vermeulen, Phung & Truong, 2007: 91, fig. 5.
Montapiculus pyramidalis — Schileyko 2011: 4.
Acinolaemus pyramidalis — Vermeulen et al. 2019: 32–34, fig. 52; Sutcharit et al. 2023: 1289–1290, fig. 1, table 1; Tongkerd et al. 2025: 53, table 4.

##### Type locality.

“Vietnam, Kien Giang Prov., Kien Luong, Hon Chong Hill”.

##### Differential diagnosis.

This species, in comparison to *C. carcharodon*, has no hooked barriers and it possesses a less depressed shell. See also under *C. rectus*.

##### Distribution.

This species is known from Kien Giang Province in Vietnam and Kampot Province in Cambodia.

##### Remarks.

This species is transferred to *Clostophis* due to the similar apertural barrier arrangement and morphology as in *C. lacrima*, *C. obliquus* and *C. socialis* (i.e., strong and moderately long barriers, including two strong palatal plicae). Furthermore, *C. pyramidalis* lacks the peculiar honeycomb-like surface sculpture of the shell (present in many *Acinolaemus* species) and peristomal pliculae (present in *A. ptychochilus*, the type species of *Acinolaemus*).

#### Clostophis rectus

Taxon classification

Animalia

StylommatophoraHypselostomatidae

(Vermeulen, Luu, Theary & Anker, 2019) comb. nov.

E4887286-0B3F-5D74-9EC2-2EA502111CC8

Fig. 44

Acinolaemus rectus Vermeulen, Luu, Theary & Anker, 2019: 34–35, 52, 54–56.
Acinolaemus rectus — Sutcharit et al. 2023: 1289–1290, fig. 1, table 1; Tongkerd et al. 2025: 53, table 4.

##### Type locality.

“Cambodia: Kampot Province, Kampong Trach area: Phnom Kampong Trach”.

**Figure 44.**
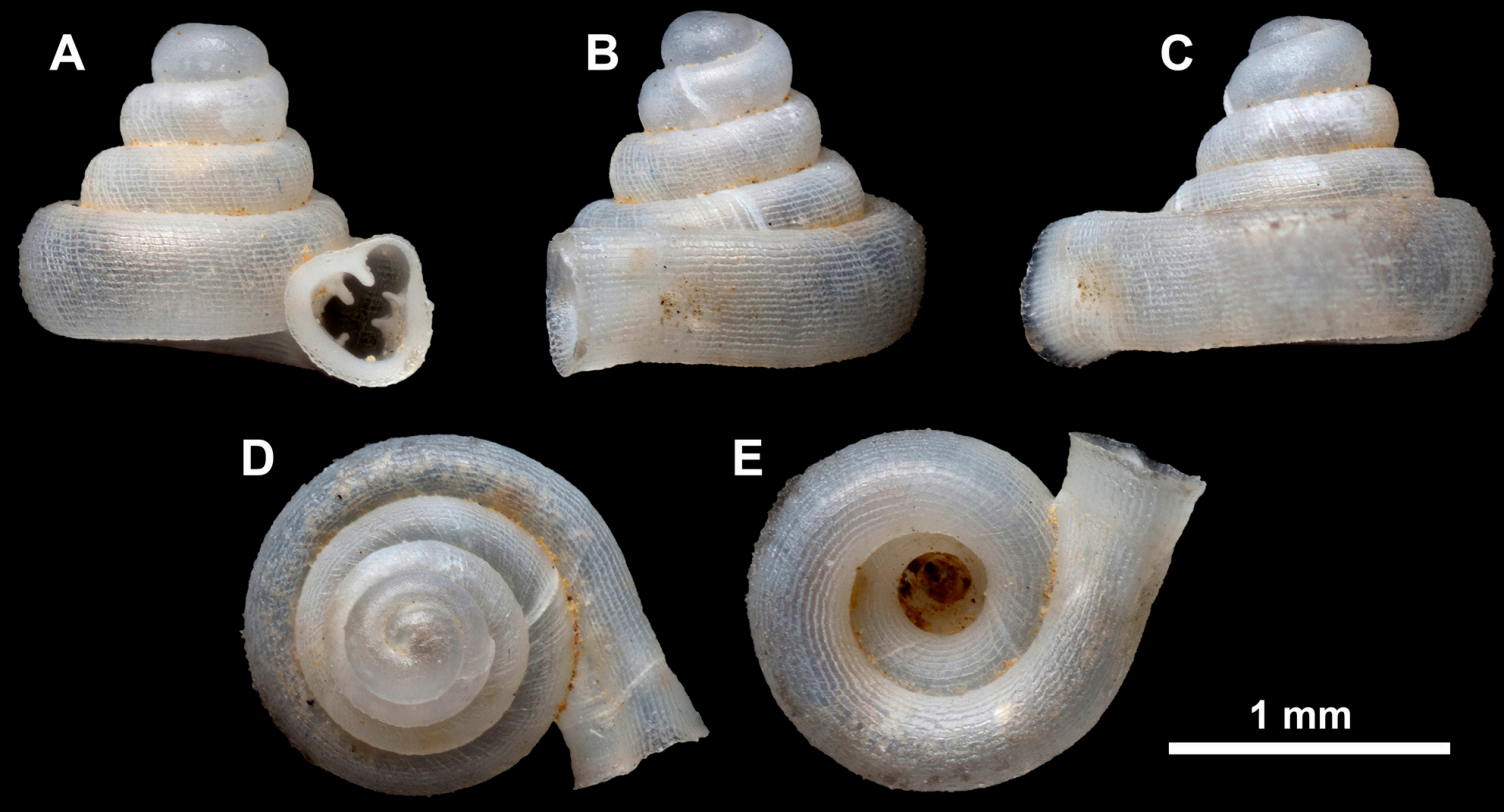
*Clostophis rectus*, holotype (NHMUK 20180572). **A–E**. Shell.

##### Type material examined.

**Cambodia** • holotype; NHMUK 20180572.

##### Differential diagnosis.

This species differs from *A. pyramidalis* by the slightly upturning aperture near the last whorl (down-turning in *A. pyramidalis*). See also under *C. rhamphodon* and *C. sphinctinion*.

##### Distribution.

This species is known only from Kampot Province in Cambodia.

##### Remarks.

This species is transferred to *Clostophis* due to the similar apertural barrier arrangement and morphology as in *C. lacrima*, *C. obliquus* and *C. socialis* (i.e., strong and moderately long barriers, including two strong palatal plicae). Furthermore, *C. pyramidalis* lacks the peculiar honeycomb-like shell surface sculpture (present in many *Acinolaemus* species) and peristomal pliculae (present in *A. ptychochilus*, the type species of *Acinolaemus*).

#### Clostophis rhamphodon

Taxon classification

Animalia

StylommatophoraHypselostomatidae

(F. G. Thompson & Upatham, 1997) comb. nov.

CF163693-EEDE-50A0-8A3C-32F980B1CD25

Fig. 45

Acinolaemus rhamphodon F. G. Thompson & Upatham, 1997: 227–228, figs 4, 20–25.
Acinolaemus *ramphodon* [sic] — Changlom et al. 2019: 156.
Acinolaemus rhamphodon — Panha and Burch 2008: 43–44, fig. 41; Dumrongrowjattana and Wongkamhaeng 2024: 321, 323, 330, 331, fig. 7, 13, table 3, 5; Tongkerd et al. 2025: 53, table 4.

##### Type locality.

“Thailand, Chachaengsao Province, Khao Tam Raet, 5 km ENE Ban Nan Khok, 100 m altitude (13°26.1'N, 101°44.2'E)”.

**Figure 45.**
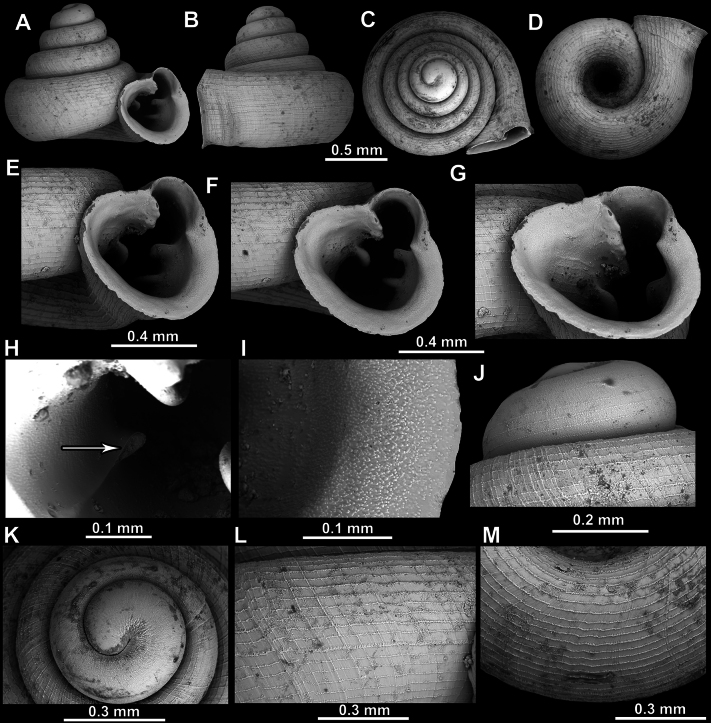
*Clostophis rhamphodon*, holotype (UF 113505). **A–D**. Shell; **E–G**. Enlarged views of the aperture; **H**. Enlarged view of the columellar lamella (hook broken off, indicated by white arrow); **I**. Enlarged view of the peristome surface; **J, K**. Enlarged views of the protoconch; **L**. Enlarged view of the last whorl surface sculpture; **M**. Enlarged view of the last whorl surface sculpture near the umbilicus.

##### Type material examined.

**Thailand** • holotype; UF 113505 • 1 paratype; UF 113506.

##### Additional material examined.

**Thailand** • 4 shells; Rayong Province, Khao Chamao District, rock wall behind Wat Tham Khao Bot; 13°02.273'N, 101°38.120'E; 70 m a.s.l.; 09 Mar 2023; A. Hunyadi leg.; coll. HA • 21 shells; Chonburi Province, Bo Thong District, rock wall behind Wat Khao Ha Yot; 13°09.775'N, 101°35.858'E; 150 m a.s.l.; 09 Mar 2023; A. Hunyadi leg.; coll. HA • 52 shells; Rayong Province, Khao Chamao District, Wat Tham Khao Pratun, gorge above the temple; 13°04.439'N, 101°35.850'E; 115 m a.s.l.; 09 Mar 2023; A. Hunyadi leg.; coll. HA.

##### Differential diagnosis.

*Clostophis rectus* has a wider umbilicus, narrower (lower) last whorl and lacks the hooked columellar lamella. See under *C. sphinctinion* and *C. stenopus*.

##### Distribution.

This species is known from Chachoengsao, Rayong, Chanthaburi and Chonburi provinces, Thailand.

##### Remarks.

This species is transferred to *Clostophis* due to the similar apertural barrier arrangement and morphology as in *C. lacrima*, *C. obliquus*, and *C. socialis* (i.e., strong and moderately long barriers, including two strong palatal plicae). Furthermore, *C. rhamphodon* lacks both the peculiar honeycomb-like shell surface sculpture (present in many *Acinolaemus* species) and peristomal pliculae (present in *A. ptychochilus*, the type species of *Acinolaemus*). Specimens from Wat Tham Khao Bot (Rayong Province) have a blunt (not hooked) columellar lamella. Since this was the only significant difference from typical *A. rhamphodon* we could find, we treat this as intraspecific variability. See Dumrongrojwattana and Wongkamhaeng (2024) for additional sampling sites.

#### Clostophis sphinctinion

Taxon classification

Animalia

StylommatophoraHypselostomatidae

(F. G. Thompson & Upatham, 1997) comb. nov.

F9B1E4B1-CD23-5DC7-8C0C-BB755E351CEE

Fig. 46

Acinolaemus sphinctinion F. G. Thompson & Upatham, 1997: 226–227, figs 16–19.
Acinolaemus sphinctinion — Panha and Burch 2008: 44–45, fig. 42; Changlom et al. 2019: 156; Dumrongrowjattana and Wongkamhaeng 2024: 321, 323, 330, fig. 7, 13, table 3; Tongkerd et al. 2025: 53, table 4.

##### Type locality.

“Thailand, Prachuap Khiri Khan Province, Khao Sam Roi Yoi National Park, limestone knoll on east side of brackish marsh 3 km south of park entrance; (12°12.5'N, 99°59.6'E)”.

**Figure 46.**
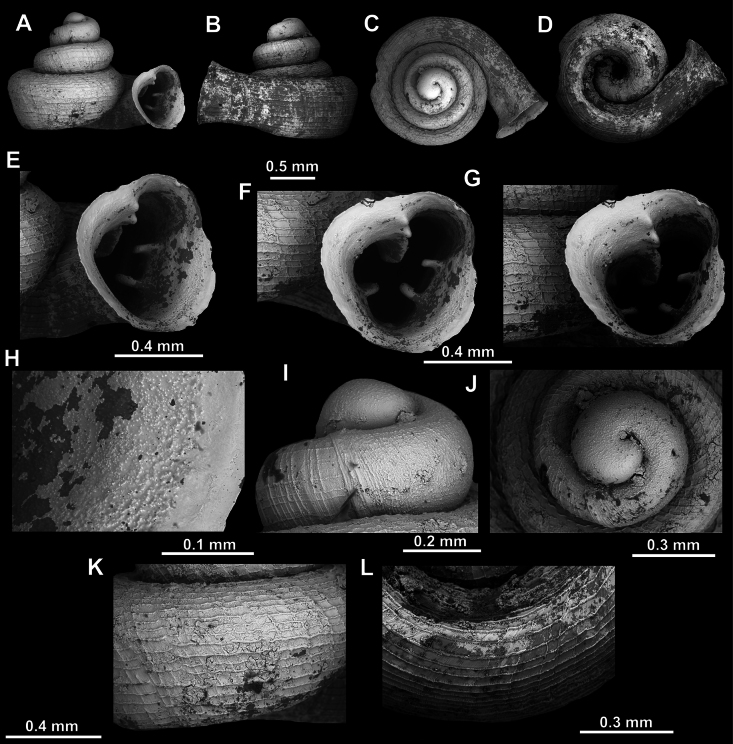
*Clostophis sphinctinion*, holotype (UF 113507). **A–D**. Shell; **E–G**. Enlarged views of the aperture; **H**. Enlarged view of the peristome surface; **I, J**. Enlarged views of the protoconch; **K**. Enlarged view of the last whorl surface sculpture; **L**. Enlarged view of the last whorl surface sculpture near the umbilicus.

##### Type material examined.

**Thailand** • holotype; UF 113507.

##### Differential diagnosis.

This species differs from *C. rectus* and *C. rhamphodon* by the merged angular and parietal lamellae into the angulo-parietal. It also has more angled whorls and a narrower (lower) last whorl with a distinct constriction behind the aperture.

##### Distribution.

This species is known only from the type locality.

##### Remarks.

This species is provisionally assigned to *Clostophis* due to similar apertural barrier arrangement as in *C. lacrima*, *C. obliquus* and *C. socialis*. It does not have a honeycomb-like shell surface, or peristomal pliculae, and its protoconch is not spirally striated which is why it is excluded from *Acinolaemus*. Lastly, it has a merged angulo-parietal lamella (separated in all *Acinolaemus* species). It is possible that this species might warrant its own genus, introducing a new name herein would be premature due to the unclear generic boundaries in the entire family.

#### Clostophis stenopus

Taxon classification

Animalia

StylommatophoraHypselostomatidae

(F. G. Thompson & Upatham, 1997) comb. nov.

BF1879DF-1061-5F9E-8FDA-2FE4255B0BF2

Fig. 47

Acinolaemus stenopus F. G. Thompson & Upatham, 1997: 228–229, figs 2, 12–15.
Acinolaemus stenopus — Changlom et al. 2019: 156; Panha and Burch 2008: 45–46, fig. 43; Dumrongrowjattana and Wongkamhaeng 2024: 321, 323, 330, 331, fig. 7, 13, table 3, 5; Tongkerd et al. 2025: 53, table 4.

##### Type locality.

“Thailand, Chanthaburi Province, limestone ridge 3 km w N Yai Ham; 25 m altitude (12°44.3'N, 101°52.9'E)”.

**Figure 47.**
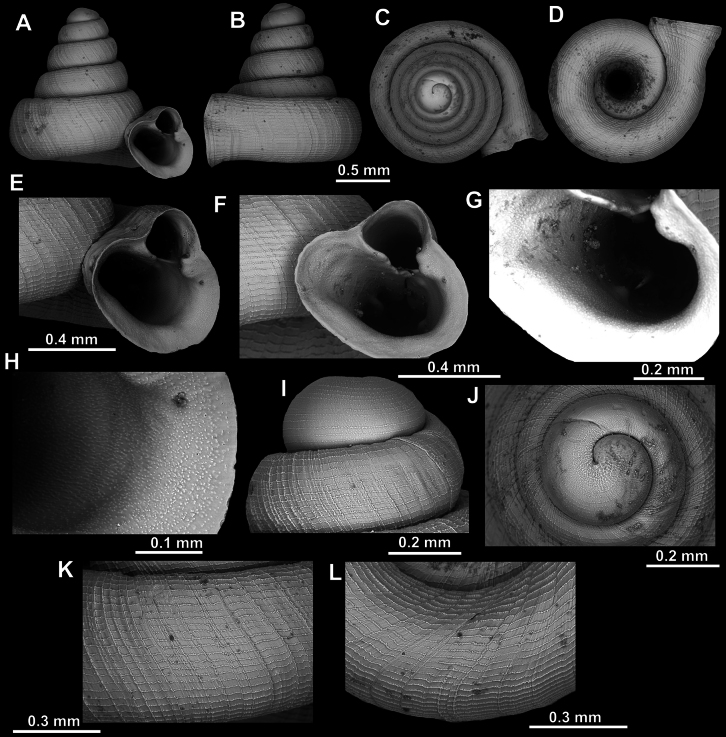
*Clostophis stenopus*, holotype (UF 113530). **A–D**. Shell; **E, F**. Enlarged views of the aperture; **G**. Enlarged view of the columellar lamella; **H**. Enlarged view of the peristome surface; **I, J**. Enlarged views of the protoconch; **K**. Enlarged view of the last whorl surface sculpture; **L**. Enlarged view of the last whorl surface sculpture near the umbilicus.

##### Type material examined.

**Thailand** • holotype; UF 113530 • 5 paratypes; UF 113531.

##### Differential diagnosis.

This species differs from *C. rhamphodon* most clearly by the presence of a strong and curved angular lamella which is directed towards the upper palatal plica and almost merging with it (leaving only a very narrow passage) and enclosing the sinulus.

##### Distribution.

This species is known from Chanthaburi and Rayong provinces, Thailand (Dumrongrojwattana and Wongkamhaeng 2024).

##### Remarks.

This species is excluded from *Acinolaemus* due to the apertural barrier arrangement which is strikingly different from *A. ptychochilus* and more similar to some *Clostophis* species, namely *C. lacrima*, *C. obliquus* and *C. socialis*. The trend of the curved angular lamella almost merging with the upper palatal plica in *C. stenopus* is also apparent in cases of the angulo-parietal lamella in mentioned *Clostophis* species.

## Discussion

Even when *Acinolaemus* was erected by Thompson and Upatham (1997), several species showed different shell morphology from the provided diagnosis. The stronger angular lamella could not be applied to *Acinolaemus colpodon*, as this species has only a single lamella on the parietal side. The radial growth lines are also much weaker than the spiral striae in this species. In *A. sphinctinion*, there is also only a single, angulo-parietal lamella, on the parietal side, which is why the first diagnostic feature is also not applicable. In addition, the protoconch in this species does not have spiral striae. Furthermore, although *A. rhamphodon* and *A. stenopus* fulfil all four diagnostic features, they have a completely different morphology of the apertural barriers from the type species, *A. ptychochilus*, which is why their assignment to the genus is also questionable. Thus, *A. ptychochilus* is the only originally described *Acinolaemus* species that matches the combination of diagnostic characters given for the genus. The other previously described species, *A. cryptidentatus*, *A. mueangonensis*, and *A. dayanus*, all match the diagnostic characters given by Thompson and Upatham (1997), so we consider them to be typical representatives of the genus. On the other hand, *A. rectus*, *A. pyramidalis* and *A. carcharodon* have a completely different aperture barrier morphology from that of the type species, and we suspect that they do not belong to *Acinolaemus*, but most likely to *Clostophis*.

As with other hypselostomatid genera (Gojšina et al. 2025), *Acinolaemus* cannot be diagnosed with a single genus-typical feature. The enlarged angular lamella as the strongest barrier in the aperture (Thompson and Upatham 1997) is not applicable, as it can be approximately equally developed (e.g., *A. mueangonensis*, *A. humilis* sp. nov., *A. paucidentatus* sp. nov., *A. profundus* sp. nov.) or even weaker than the parietal (e.g., *A. dayanus*). A well-distinct sinulus formed by the close arrangement of the parietal lamella and upper palatal plica/palatal tubercle is a feature often shared with some species of *Boysidia* (e.g., *B. hupeana* (Gredler, 1901)) or even *Clostophis* (e.g., *C. socialis*). The teleoconch surface sculpture of equally strong spiral striae and radial lines does not apply to all *Acinolaemus* species, as many of them have a honeycomb-like surface or much weaker radial growth lines. Finally, the presence of spiral striae on the protoconch also applies to other genera such as *Clostophis* (Páll-Gergely and Hunyadi 2022), *Angustopila* (Páll-Gergely et al. 2023), *Anauchen*, *Bensonella* and *Hypselostoma* (Gojšina et al. 2025). For this reason, a combination of characters, rather than a single character should be used to distinguish *Acinolaemus* from other genera. *Acinolaemus* differs from *Angustopila*, in having a much more complex surface sculpture, more apertural barriers and a larger size. *Clostophis* is probably the most similar genus. In contrast to *Acinolaemus*, *Clostophis* never has the honeycomb-like surface and many *Acinolaemus* have much more apertural barriers which are also much more slender and sometimes reach the peristome as small pliculae. Of all *Acinolaemus* species, only *A. paucidentatus* sp. nov., *A. simplex* sp. nov., and *A. atypicus* sp. nov. have a small number of barriers with morphology comparable to *Clostophis*. However, *A. simplex* sp. nov. and *A. atypicus* sp. nov. both have much more pronounced surface sculpture and *A. paucidentatus* sp. nov. has peristomal pliculae as well as surface sculpture strikingly similar to the type species, *A. ptychochilus*. Lastly, shell shape of *Acinolaemus* is, with only a few exceptions (e.g., *A. altus* sp. nov., *A. simplex* sp. nov.), always more “box”-shaped than *Clostophis* due to the shouldered last whorl and low shell height to width ratio. *Acinolaemus* can also be similar to *Bensonella* species, as it shares the typical arrangement of three apertural barriers on the parietal side (angular, parietal, infraparietal) and a palatal tubercle. In contrast to *Bensonella*, *Acinolaemus* species are usually colorless or very light yellowish/brownish with a more strongly expanded last whorl (developing a stronger concave-conical shape, and consequently, a wider umbilicus), a more distinctly separated sinulus, and a more prominent surface sculpture.

Gojšina et al. (2025) have shown that simple variations in the number of minor apertural barriers should not be taken into account when differentiating hypselostomatid species. This also applies to *Acinolaemus*. The mere presence or absence of minor apertural barriers (e.g., subcolumellar, supracolumellar, suprapalatal, etc.) varies both inter- and intra-populationally and should not be used as a feature to distinguish species. The morphology of the apertural barriers is much more important. In *A. mueangonensis*, the apertural barriers are very slender and extend deep into the aperture projecting towards the peristome. In *A. corusticorus*, a species with a very similar arrangement of apertural barriers, the barriers are much shorter and do not extend as deeply into the aperture, nor do they project as slenderly towards the peristome, which is a feature that clearly distinguishes it from *A. mueangonensis* (Tongkerd et al. 2025). The morphology of the barriers (hooked or non-hooked) is also important for species identification, but individual variations are still treated as intraspecific variability. Normally, *A. ptychochilus* has a hooked lower palatal plica (this applies to the type series as well as to specimens from Nan Province, see Figs 34, 35). In the specimens from Chiang Rai Province (Fig. 38), the lower palatal plica was not hooked, but as this was the only difference observed, we treat this population as *A. ptychochilus*.

Different types of surface sculpture were considered crucial in distinguishing the species. On the other hand, variations in sculpture density within one type were not considered important if they were not coupled with other morphological differences. The specimens of *A. ptychochilus* from the type locality had less dense sculpture than those from Nan Province, but this was the only difference noted.

We have found that shell slenderness can vary between different populations of a species or even within a population. The specimens of *A. mueangonensis* from Tak Province reported by Tongkerd et al. (2025) were morphologically virtually identical to those from the type locality in Chiang Mai Province, except for the clearly much more slender shell. On the other hand, the holotype of *A. corusticorus* is more slender than the illustrated paratypes.

This project increases the total number of *Acinolaemus* species to 19. The distribution of the genus has also been altered, now being absent from Vietnam and Cambodia and with a much greater diversity in both Thailand and Myanmar. The genus’ westernmost localities are in Shan State, Myanmar and the easternmost in Loei Province, Thailand. The northernmost localities are in Chiang Rai Province and the southernmost are in Tak Province, both in Thailand.

## Supplementary Material

XML Treatment for Acinolaemus

XML Treatment for Acinolaemus altus

XML Treatment for Acinolaemus atypicus

XML Treatment for Acinolaemus cryptidentatus

XML Treatment for Acinolaemus humilis

XML Treatment for Acinolaemus simplex

XML Treatment for Acinolaemus solitus

XML Treatment for Acinolaemus asper

XML Treatment for Acinolaemus corusticorus

XML Treatment for Acinolaemus dayanus

XML Treatment for Acinolaemus microcubus

XML Treatment for Acinolaemus mueangonensis

XML Treatment for Acinolaemus rhamphodontis

XML Treatment for Acinolaemus zed

XML Treatment for Acinolaemus

XML Treatment for Acinolaemus ferox

XML Treatment for Acinolaemus profundus

XML Treatment for Acinolaemus paucidentatus

XML Treatment for Acinolaemus ptychochilus

XML Treatment for Acinolaemus singularis

XML Treatment for Acinolaemus rugolabialis

XML Treatment for Clostophis

XML Treatment for Clostophis carcharodon

XML Treatment for Clostophis colpodon

XML Treatment for Clostophis pyramidalis

XML Treatment for Clostophis rectus

XML Treatment for Clostophis rhamphodon

XML Treatment for Clostophis sphinctinion

XML Treatment for Clostophis stenopus

## Additional information

### Artificial Intelligence (AI) use

The authors accept full responsibility for the content of the manuscript, including the disclosure of any use of AI.

No AI tools were used in the preparation of this manuscript.

### Author ORCIDs

V. Gojšina https://orcid.org/0000-0002-0413-9304

A. Hunyadi https://orcid.org/0000-0003-3488-2817

C. Sutcharit https://orcid.org/0000-0001-7670-9540

P. Tongkerd https://orcid.org/0000-0001-9221-9293

K. Auffenberg https://orcid.org/0000-0001-9660-6776

J. Grego https://orcid.org/0000-0002-4977-0415

B. Páll-Gergely https://orcid.org/0000-0002-6167-7221

### Data availability

All of the data that support the findings of this study are available in the main text.
